# Characterization of the Streptomyces coelicolor Glycoproteome Reveals Glycoproteins Important for Cell Wall Biogenesis

**DOI:** 10.1128/mBio.01092-19

**Published:** 2019-06-25

**Authors:** Tessa Keenan, Adam Dowle, Rachel Bates, Margaret C. M. Smith

**Affiliations:** a Department of Biology, University of York, York, United Kingdom; b Bioscience Technology Facility, University of York, York, United Kingdom; University of British Columbia

**Keywords:** *Actinobacteria*, antibiotic resistance, cell wall biogenesis, glycopeptides, mass spectrometry, protein O-glycosylation, protein O-mannosyltransferase

## Abstract

The physiological role of protein O-glycosylation in prokaryotes is poorly understood due to our limited knowledge of the extent of their glycoproteomes. In *Actinobacteria*, defects in protein O-mannosyl transferase (Pmt)-mediated protein O-glycosylation have been shown to significantly retard growth (Mycobacterium tuberculosis and Corynebacterium glutamicum) or result in increased sensitivities to cell wall-targeting antibiotics (Streptomyces coelicolor), suggesting that protein O-glycosylation has an important role in cell physiology. Only a single glycoprotein (SCO4142, or PstS) has been identified to date in S. coelicolor. Combining biochemical and mass spectrometry-based approaches, we have isolated and characterized the membrane glycoproteome in S. coelicolor. A total of ninety-five high-confidence glycopeptides were identified which mapped to thirty-seven new S. coelicolor glycoproteins and a deeper understanding of glycosylation sites in PstS. Glycosylation sites were found to be modified with up to three hexose residues, consistent with what has been observed previously in other *Actinobacteria*. S. coelicolor glycoproteins have diverse roles and functions, including solute binding, polysaccharide hydrolases, ABC transporters, and cell wall biosynthesis, the latter being of potential relevance to the antibiotic-sensitive phenotype of *pmt* mutants. Null mutants in genes encoding a putative d-Ala-d-Ala carboxypeptidase (SCO4847) and an l,d-transpeptidase (SCO4934) were hypersensitive to cell wall-targeting antibiotics. Additionally, the *sco4847* mutants displayed an increased susceptibility to lysozyme treatment. These findings strongly suggest that both glycoproteins are required for maintaining cell wall integrity and that glycosylation could be affecting enzyme function.

## Abstract (precis)

In prokaryotes, the role of protein glycosylation is poorly understood due to our limited understanding of their glycoproteomes. In some *Actinobacteria*, defects in protein O-glycosylation have been shown to retard growth and result in hypersensitivity to cell wall-targeting antibiotics, suggesting that this modification is important for maintaining cell wall structure. Here, we have characterized the glycoproteome in Streptomyces coelicolor and shown that glycoproteins have diverse roles, including those related to solute binding, ABC transporters, and cell wall biosynthesis. We have generated mutants encoding two putative cell wall-active glycoproteins and shown them to be hypersensitive to cell wall-targeting antibiotics. These findings strongly suggest that both glycoproteins are required for maintaining cell wall integrity and that glycosylation affects enzyme function.

## INTRODUCTION

Protein modification by glycosylation is a process that occurs in all domains of life (1, 2). Glycan moieties, which can be extremely diverse in structure and composition, are most commonly attached to either asparagine (N-glycosylation) or to serine/threonine (O-glycosylation) in the peptide chain. The presence of the glycan changes the physicochemical properties of the protein and has been shown to have effects on cellular localization, ligand binding, and stability (1). The enzymes mediating N- and O-glycosylation are conserved between kingdoms, but studies on protein glycosylation in prokaryotes lags behind that of eukaryotes. Consequently, with few exceptions (3–5), the extent and functions of the glycoproteome in most prokaryotes are unclear.

Recent reports have described the phenotypes of bacteria lacking a protein-O-mannosyl transferase (Pmt), and they are either strongly retarded in growth (Mycobacterium tuberculosis and Corynebacterium glutamicum) or have increased sensitivities to several antibiotics that target the cell wall, including vancomycin and β-lactams (Streptomyces coelicolor) (6–8). These three bacterial species are all within the *Actinobacteria*, where the occurrence of Pmt is prevalent. In the case of *Streptomyces*, the *pmt* mutants have also become resistant to infection by the phage φC31, implying that the glycans perform a role in ligand recognition (9). The O-glycoproteome from Mycobacterium tuberculosis has been extensively explored; in particular, the culture filtrate consists of more than forty glycoproteins, including potential cell wall-active glycoproteins, such as a putative glycosyl hydrolase (Rv1096) and the β-lactamase BlaC (Rv2068c) (10–14). In contrast, only a single glycoprotein (SCO4142; PstS) has been identified to date in S. coelicolor (15). Given that the Pmt-mediated O-glycosylation system is a general glycosylation system in other bacteria and fungi, we hypothesize the presence of a glycoproteome in S. coelicolor and that one of its roles is in cell wall biogenesis.

Pmt is a predicted integral membrane protein, and in M. tuberculosis it has been shown to mannosylate unfolded proteins as they are secreted through the Sec system (16). The sugar donor for Pmt is polyprenol phosphate mannose (PPM), which is made intracytoplasmically via the transfer of mannose from GDP-mannose to polyprenol phosphate by polyprenol phosphate mannose synthase (Ppm1) (15, 17). PPM is then thought to be flipped in the membrane so that the mannose moiety can be presented to Pmt for transfer to the target proteins. S. coelicolor *ppm1* mutants, and mutants (*manB* and *manC*) with depleted enzymes that supply GDP-mannose to Ppm1, all have phenotypes that resemble that of the *pmt* mutants but display more extreme antibiotic sensitivities (6, 18).

The phenotypes of the *pmt* mutants imply that glycosylation has an important role in cell physiology. The increased sensitivity of the S. coelicolor *pmt* mutants to the antibiotics vancomycin and some β-lactams suggests that glycosylation affects the function of enzymes in cell wall biogenesis, possibly in peptidoglycan cross-linking. Here, we investigate the *Streptomyces* glycoproteome, focusing on the membrane and membrane-associated proteins with a view to elucidating the mechanism that underpins the antibiotic sensitivity. Using enrichment of the glycoproteome by lectin affinity chromatography followed by mass spectrometry, a total of ninety-five high-confidence glycopeptides were characterized from thirty-eight glycoproteins. S. coelicolor mutants were constructed in genes encoding glycoproteins that could be involved in peptidoglycan biosynthesis and were found to have an antibiotic-sensitive phenotype. These data indicate that protein glycosylation has a role in the functions of multiple periplasmic proteins.

## RESULTS AND DISCUSSION

### Enrichment and detection of a glycoproteome in S. coelicolor.

To investigate the glycoproteome in S. coelicolor, membrane protein fractions were isolated from the S. coelicolor parent strain J1929 and the glycosylation-deficient strains DT1025 (*pmt* mutant) and DT3017 (*ppm1* mutant). The strains were cultivated in defined, phosphate-limited (F134) liquid medium, as expression of the previously characterized S. coelicolor glycoprotein SCO4142 (PstS) was known to be induced on phosphate depletion (15, 19, 20). The proteins were separated by SDS-PAGE, blotted onto polyvinylidene difluoride (PVDF) membrane, and probed with concanavalin A (ConA) conjugated to horseradish peroxidase (ConA-HRP) (see Fig. S1 in the supplemental material). Several ConA-reactive bands were observed in the J1929 membrane protein fraction within the 100- to 40-kDa molecular weight range that were absent from the protein O-mannosyl transferase- and polyprenol phosphate mannose synthase-deficient strains DT1025 (*pmt* mutant) and DT3017 (*ppm1* mutant) (Fig. S1B). The ConA reactivity was lost in the presence of methyl α-d-glucopyranoside, a competitive inhibitor of mannose and glucose binding. These results demonstrate the presence of a glycoproteome in S. coelicolor that requires the activities of Pmt and Ppm1.

10.1128/mBio.01092-19.3 FIG S1 Detection of glycosylated proteins in the membrane proteome of S. coelicolor J1929 using ConA-HRP. Download FIG S1, DOCX file, 0.7 MB. Copyright © 2019 Keenan et al. 2019 Keenan et al. This content is distributed under the terms of the Creative Commons Attribution 4.0 International license.

**FIG 1:**
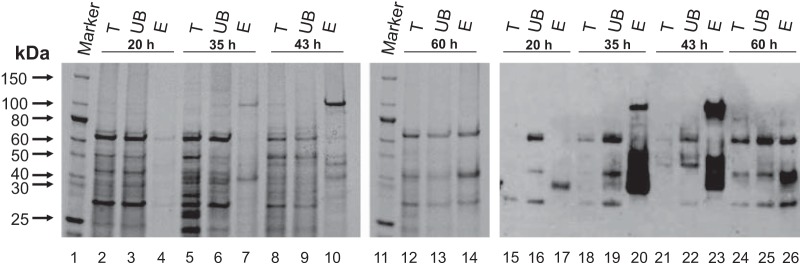
Glycoprotein enrichment time course by ConA affinity chromatography. Total membrane (T), unbound membrane (UB), and eluted (E) protein fractions were separated by SDS-PAGE and stained with protein stain (lanes 1 to 14) or probed with ConA-HRP after Western blotting (lanes 15 to 26).

To facilitate the characterization of the glycoproteome, lectin affinity chromatography was used to enrich for the S. coelicolor membrane glycoproteins. In order to maximize the number of glycoproteins isolated and to account for any growth stage-specific changes to the glycoproteome, glycoproteins were enriched from J1929 membrane protein fractions isolated after 20, 35, 43, and 60 h of growth (Fig. S2). The total, unbound, and enriched protein fractions were separated by SDS-PAGE, blotted onto a PVDF membrane, and probed with ConA-HRP (Fig. 1). Over the four time points, changes were observed in the abundance and numbers of proteins enriched after lectin affinity chromatography, as shown by Coomassie staining (Fig. 1, lanes 4, 7, 10, and 14), suggesting that there are growth stage-specific changes to the membrane glycoproteome in S. coelicolor. The ConA reactivity profiles of the enriched fractions, which also changed throughout the time course, are consistent with this observation (Fig. 1, lanes 17, 20, 23, and 26). The greatest number of strongly ConA-reactive bands were observed in membrane protein fractions enriched after 35 and 43 h of growth, suggesting that these fractions yield the most glycoproteins. The unbound fractions from the ConA columns also yielded some cross-reactivity with ConA-HRP but mostly to proteins that were abundant in the Coomassie-stained gels, suggesting nonspecific ConA reactivity. Taken together, these results show that glycoproteins are expressed throughout the S. coelicolor growth cycle and that the glycoproteome varies according to the growth stage.

10.1128/mBio.01092-19.4 FIG S2 Growth of S. coelicolor J1929 in liquid F134 medium. Download FIG S2, DOCX file, 0.7 MB. Copyright © 2019 Keenan et al. 2019 Keenan et al. This content is distributed under the terms of the Creative Commons Attribution 4.0 International license.

### S. coelicolor glycoproteome characterization using mass spectrometry.

In order to identify the S. coelicolor glycoproteins isolated from the membrane proteome after lectin affinity chromatography (Fig. 1) and characterize the sites of modification, liquid chromatography (LC) coupled to tandem mass spectrometry (MS/MS) was carried out. Since the previously characterized S. coelicolor glycoprotein PstS was shown to be modified with a trihexose (15) and numerous glycoproteins with short mannose modifications have been previously described in the closely related M. tuberculosis (10, 13, 21), we focused on short hexose modifications in our analyses. To enable a comprehensive analysis of the S. coelicolor glycoproteome, several different peptide fragmentation techniques were employed to facilitate both glycopeptide characterization and glycosylation site assignment.

**FIG 2:**
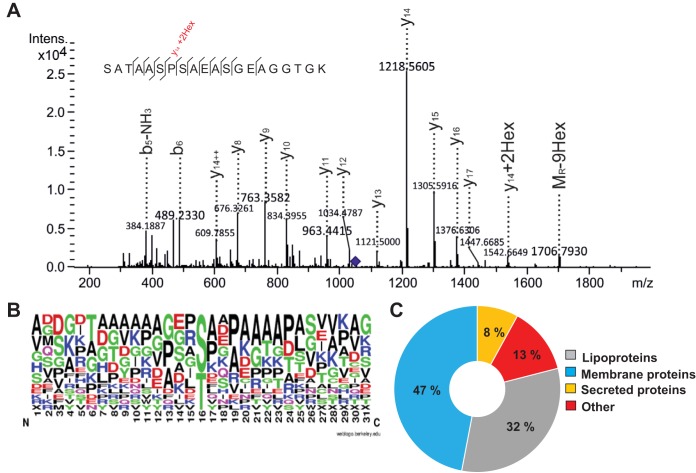
Characterization of enriched glycoproteins by mass spectrometry. (A) CID spectrum of the glycopeptide SATAASPSAEASGEAGGTGK-9Hex from SCO4847, isolated after 35 h of growth. Precursor *m/z* 1,055.7991; charge, 2+; retention time (RT), 25.7 min; e-value, 0.0003. (B) S. coelicolor O-glycosylation site motif. (C) Subcellular localization of S. coelicolor glycoproteins.

The fractions enriched in S. coelicolor glycoproteins after 20, 35, 43, and 60 h of growth were each subjected to in-gel tryptic digestion after SDS-PAGE and analyzed by liquid chromatography coupled to electrospray ionization collision-induced dissociation tandem mass spectrometry (LC-ESI-CID-MS/MS). A total of 24 different S. coelicolor glycopeptides were identified over the four time points (Data Set S1), mapping to fifteen new S. coelicolor glycoproteins. The spectra of the glycopeptides obtained by CID fragmentation were dominated by product ions formed due to the preferential cleavage of glycosidic bonds. In these cases, the glycopeptide was identified when the mass difference between the peptide backbone identified from the MS/MS spectra and the precursor ion was equivalent to a hexose (162 Da) or multiples thereof. For example, the glycopeptide N- SATAASPSAEASGEAGGTGK-C, belonging to SCO4847, was shown to be modified with nine hexose residues (Fig. 2A). The triply charged precursor ion at *m/z* 1,055.76 is consistent with a glycopeptide mass of 3,164.28 Da. The predicted mass of unmodified N-SATAASPSAEASGEAGGTGK-C is 1,705.77 Da, which is a difference of 9 hexose residues (1,458.47 Da) from the mass of the glycosylated peptide. The spectrum is dominated by the *y*-ion series that validate the sequence of the peptide backbone. While two ions were observed with the glycan intact (*y*_14_ + 2Hex and M_R_ + 9Hex), these were not enough to assign the glycosylation sites in the glycopeptide. Since the unambiguous assignment of the glycosylated amino acid residue relies on the observation of peptide product ions containing at least one hexose residue, in many cases it was not possible to map the glycosylation sites in the glycopeptides identified using CID fragmentation.

10.1128/mBio.01092-19.7 DATA SET S1 High-confidence glycopeptides identified in the LC-ESI-MS/MS experiments with CID, HCD, and ETD fragmentation. Download Data Set S1, XLSX file, 0.02 MB. Copyright © 2019 Keenan et al. 2019 Keenan et al. This content is distributed under the terms of the Creative Commons Attribution 4.0 International license.

To widen the S. coelicolor glycoproteome characterization and to enable glycosylation site assignments to be made, enriched membrane glycoproteins isolated after 43 h of growth were further analyzed by mass spectrometry using the complementary fragmentation techniques, higher-energy collision dissociation (HCD) and electron transfer dissociation (ETD). HCD fragmentation is a higher-energy form of CID available on Orbitrap mass spectrometers and produces fragmentation patterns similar to those of CID fragmentation (*y-* and *b*-type ions). In contrast, ETD fragmentation favors cleavage of the peptide backbone (*c*- and *z*-type ions), leaving the glycan structure intact, thereby facilitating glycosylation site localization (22). The combined data acquisitions using the HCD and ETD fragmentation techniques resulted in the identification of thirty-six different S. coelicolor glycopeptides (Data Set S1). The spectrum with the highest confidence of a match for each glycopeptide is shown in Data Set S2. ETD fragmentation allowed a further thirteen O-glycosylation sites to be assigned, nearly double the number of assignments made after the CID and HCD experiments combined. In total, O-glycosylation sites were assigned in approximately 30% of the glycopeptides identified in this work. While no distinct consensus sequence was identified, there was a high propensity for hydrophobic amino acids (e.g., Ala, Pro, and Gly) near the glycosylation site (Fig. 2B). This feature is reminiscent of sequences surrounding O-glycosylation sites in other *Actinobacteria* (14, 21, 23, 24). At least 30% of the glycopeptides identified in this work were supported by multiple spectra. Hex, Hex_2_, and Hex_3_ modifications were all detected, as expected. Searches for Hex_4_ and Hex_5_ modifications revealed some hits; however, upon manual inspection of these spectra it was determined that these were peptides with multiple sites modified with Hex, Hex_2_, and Hex_3_.

10.1128/mBio.01092-19.8 DATA SET S2 Spectra generated by LC-ESI-CID-MS/MS on the Bruker maXis HD and Thermo Scientific Orbitrap Fusion Tribrid systems. Download Data Set S2, PDF file, 1.3 MB. Copyright © 2019 Keenan et al. 2019 Keenan et al. This content is distributed under the terms of the Creative Commons Attribution 4.0 International license.

In total, thirty-seven new S. coelicolor glycoproteins were identified (Table 1). Additionally, the data acquired using ETD fragmentation enabled the further characterization of the previously identified S. coelicolor glycoprotein PstS (SCO4142) (15) by the assignment of two glycosylation sites (residue underlined) in glycopeptides N-DGIKTVDVK-C and N-QTPGAISYFELSYAKDGIK-C (Data Set S1). Indeed, PstS is one of the most heavily glycosylated proteins identified in this work, with at least three further glycosylation sites that could not be defined here (Fig. S3). Two of these glycopeptides overlapped the synthetic peptides that were shown previously to be glycosylated in a cell-free assay (15).

**TABLE 1:** S. coelicolor glycoproteins identified in this work

Protein	Function	TMHMM no.^*a*^	SignalP 4.1^*b*^	TatP 1.0^*c*^	LipoP 1.0^*d*^	Classification
SCO0472	Putative secreted protein		Y; 0.548	Y; 0.381	SpII; 22.2623	Lipoprotein
SCO0996	Putative metal-binding lipoprotein		Y; 0.526	N	SpI; 11.5964	Lipoprotein
SCO1714	Putative secreted protein	1	Y; 0.498	N	SpII; 12.878	Lipoprotein
SCO2838	Putative secreted endoglucanase		Y; 0.639	Y; 0.377	SpII; 32.6736	Lipoprotein
SCO3357	Hypothetical protein		N	Y; 0.492	SpII; 17.3077	Lipoprotein
SCO4142	PstS, substrate binding domain of ABC-type phosphate transporter		Y; 0.595	N	SpII; 26.7983	Lipoprotein
SCO4739	Putative lipoprotein		Y; 0.579	N	SpII; 20.7928	Lipoprotein
SCO4885	Putative nucleoside-binding lipoprotein		N	N	SpII; 23.8395	Lipoprotein
SCO4905	Putative lipoprotein		Y; 0.574	N	SpII; 13.7291	Lipoprotein
SCO4934	Putative l,d-transpeptidase		Y; 0.571	Y; 0.483	SpII; 24.1553	Lipoprotein
SCO5646	Putative thiamine-binding lipoprotein		N	Y; 0.468	SpII; 13.5061	Lipoprotein
SCO7218	Putative iron transport lipoprotein		Y; 0.632	N	SpI; 14.1761	Lipoprotein
SCO2096	Transglutaminase/protease-like membrane protein	6	Y; 0.529	N	SpII; 8.2333	Membrane
SCO2035	Putative disulfide oxidoreductase	1	N	N	N	Membrane
SCO2156	Putative cytochrome *c* oxidase subunit II	3	N	N	N	Membrane
SCO2963	Putative membrane protein	1	N	N	N	Membrane
SCO3044	Putative cell envelope-associated transcriptional attenuator LytR-CpsA-Psr	1	N	N	N	Membrane
SCO3046	Putative cell envelope-associated transcriptional attenuator LytR-CpsA-Psr	1	N	N	N	Membrane
SCO3184	Putative penicillin acylase	1	N	Y; 0.366	N	Membrane
SCO3848	Putative serine/threonine protein kinase	1	N	N	N	Membrane
SCO3891	Putative membrane protein	1	N	N	N	Membrane
SCO4013	Putative secreted penicillin-binding protein FtsI	1	N	N	N	Membrane
SCO4130	Putative integral membrane protein	1	N	N	N	Membrane
SCO4141	Phosphate ABC transport system permease protein	5	N	N	N	Membrane
SCO4256	Putative hydrolytic protein	1	N	N	N	Membrane
SCO4548	Putative integral membrane protein	3	N	Y; 0.479	N	Membrane
SCO4968	Putative membrane protein	1	N	N	N	Membrane
SCO5204	Integral membrane protein	7	N	N	N	Membrane
SCO5751	Putative membrane protein	1	N	N	N	Membrane
SCO5818	Putative ABC-type Na^+^ transport system	5	N	N	N	Membrane
SCO3540	Proteinase (putative secreted protein)	1	Y; 0.627	Y; 0.700	SpI; 18.2099	Secreted
SCO4847	DacC, putative d-alanyl-d-alanine carboxypeptidase	1	Y; 0.711	Y; 0.427	SpI; 27.3476	Secreted
SCO5776	Glutamate binding protein		Y; 0.618	N	SpI; 21.8509	Secreted
SCO3353	Hypothetical protein		N	N	N	Other
SCO4307	MurQ, *N*-acetylmuramic acid-6-phosphate etherase		N	N	N	Other
SCO5115	BldKD, putative ABC transporter intracellular ATPase subunit		N	N	N	Other
SCO5736	30S ribosomal protein S15		N	N	N	Other
SCO6558	Putative oxidoreductase		N	N	N	Other

a The number of transmembrane helices predicted by the TMHMM 2.0 server (http://www.cbs.dtu.dk/services/TMHMM/).

b SignalP 4.1 software predicts the presence of a signal peptide (http://www.cbs.dtu.dk/services/SignalP/). d-score is a score used to discriminate signal peptides from non-signal peptides. Scores of >0.450 indicate a signal peptide. Y, yes; N, no.

c TatP 1.0 predicts the presence of twin arginine (TAT) signal peptides. d-score of >0.36 predicts the presence of a TAT pathway signal.

d LipoP 1.0 software produces predictions of lipoproteins (http://www.cbs.dtu.dk/services/LipoP/). SpI denotes SEC signal peptide; SpII denotes lipoprotein.

10.1128/mBio.01092-19.5 FIG S3 PstS glycopeptides overlap with synthetic peptides previously shown to be glycosylated in a cell-free assay. Download FIG S3, DOCX file, 0.7 MB. Copyright © 2019 Keenan et al. 2019 Keenan et al. This content is distributed under the terms of the Creative Commons Attribution 4.0 International license.

Database searches were carried out in order to classify the glycoproteins as either lipoproteins, membrane proteins, or secreted proteins. Proteins were functionally annotated using the *Streptomyces* genome database (StrepDB; http://strepdb.streptomyces.org.uk/) and the Conserved Domain Database (CDD) (https://www.ncbi.nlm.nih.gov/Structure/cdd/wrpsb.cgi) (25). In some cases, the literature was contradictory to the results observed after the database searches. For example, SCO7218 is annotated as a putative iron transport lipoprotein in the StrepDB. However, the LipoP 1.0 server did not predict a lipoprotein signal peptide (SpII) in this protein. SCO7218 is upstream of an ABC transporter (SCO7216/SCO7217), which is consistent with the known genome architecture of solute binding lipoproteins in S. coelicolor (26). In these cases, the literature searches were considered to be more reliable in assigning a category to the proteins.

Protein O-glycosylation by Pmt was shown to be coupled to protein secretion via the Sec pathway in M. tuberculosis, suggesting that protein O-mannosylation should only affect extracellular proteins (16). Consistent with this precedent, more than a third of the newly identified S. coelicolor glycoproteins in this study were predicted lipoproteins and other secreted proteins (Fig. 2C). The lipoproteins included SCO3357 (CseA), which is proposed to dampen the cell envelope stress response by the two-component sensor regulators CseB and CseC, which activate the expression of the SigE-encoding gene *sco3356* (27, 28). In addition the putative lipoprotein, SCO4905 (AfsQ3) was also glycosylated and is also proposed to be a modulator of a two-component sensor regulator, AfsQ1/AfsQ2 (27). Many of the glycolipoproteins are, or are predicted to be, substrate binding proteins that interact with ABC transporters (SCO0472, SCO5776, SCO7218, SCO4885, and SCO4142). Nearly 50% of the glycoproteins identified in this study are putative membrane proteins with predicted functions, including transport (SCO4141 and SCO5818) and serine/threonine kinases (SCO3848), as well as many proteins of unknown function (SCO2963, SCO3891, SCO4130, SCO4548, SCO4968, SCO5204, and SCO5751). Additionally, five of the glycoproteins identified here had no predicted transmembrane domains or secretory signals. Three of these, SCO5736, SCO4307, and SCO5115, are very likely to be intracellular proteins; SCO5736 is a predicted S15 ribosomal subunit, SCO4307 is an *N*-acetylmuramic acid (MurNAc)-6-phosphate etherase (MurQ), an enzyme that acts intracellularly to recycle peptidoglycan MurNAc (29), and SCO5115 (BldKD) is a predicted intracellular ATPase subunit for an oligopeptide uptake system (30). Clearly, as these three proteins go against the precedent that Pmt glycosylates only extracellular proteins, further investigations are required to validate this observation.

Nearly 25% of the glycoproteins identified here are predicted to be TAT-targeted proteins. The TAT protein transport system functions to secrete folded proteins across the cytoplasmic membrane and to insert some integral membrane proteins into the membrane (31). The pathway is well characterized in S. coelicolor, and it is known to translocate large numbers of lipoproteins (26, 32). SCO4934, a predicted l,d-transpeptidase and glycoprotein identified in this study, was experimentally verified as a TAT substrate by Thompson et al. (26) after it was shown to be absent from S. coelicolor *ΔtatC* strains. In mycobacteria, the fact that protein O-glycosylation was shown to be coupled to protein translocation via the Sec pathway suggests that protein O-glycosylation occurs on unfolded proteins (16). While protein O-mannosylation in eukaryotes is conventionally thought to be coupled to protein translocation into the endoplasmic reticulum (ER), Pmt-mediated glycosylation of misfolded proteins after they have been translocated into the ER has been demonstrated (33). The translocation of glycoproteins via the TAT pathway in S. coelicolor suggests that glycosylation is also possible on folded proteins. Although Pmt has not been shown definitively to be the enzyme that glycosylates proteins secreted through the TAT pathway, one could envisage that the glycosylation occurs on surface-exposed regions of the protein or in flexible loops that link secondary structure elements.

### Glycoproteins with functions in cell wall biogenesis.

Upon characterizing the membrane glycoproteome in S. coelicolor, we were particularly interested in proteins that could help to explain the antibiotic hypersensitivity phenotypes observed previously in the *pmt* and *ppm1* mutant S. coelicolor strains (6). It was hypothesized that the S. coelicolor glycoproteome could contain proteins that are important in cell wall biosynthesis or for maintaining membrane integrity. In this study, at least seven glycoproteins have been identified that have predicted functions in the cell wall (SCO4934, SCO4847, SCO3044, SCO3046, SCO3184, SCO4013, and SCO4307). SCO4847, for example, is a putative d-Ala-d-Ala carboxypeptidase and low-molecular-weight penicillin-binding protein. These proteins are thought to catalyze the hydrolysis of the terminal d-alanine from the peptidoglycan stem peptide (34). SCO4013 is another predicted penicillin binding protein, while SCO4934 is a predicted l,d-transpeptidase. l,d-Transpeptidases catalyze an alternative type of peptidoglycan cross-linking between the third-position amino acids of tetrapeptide stems, termed 3→3 cross-linking. l,d-transpeptidases have been identified in M. tuberculosis and were shown to be important for maintaining cell shape, virulence, and resistance to β-lactam antibiotics (35). SCO3044 and SCO3046 both belong to the LytR-CpsA-Psr (LCP) family of proteins that were first shown to catalyze the ligation of wall teichoic acids (WTA) to the MurNAc units of peptidoglycan in Bacillus subtilis (36). Other studies have demonstrated that LCP proteins are required to attach the capsular polysaccharide to peptidoglycan in both Staphylococcus aureus and Streptococcus pneumoniae (37, 38). Recently, however, an LCP protein in M. tuberculosis (Lcp1) was shown to be required for cell viability and to attach arabinogalactan to peptidoglycan in a cell-free assay (39).

**FIG 3:**
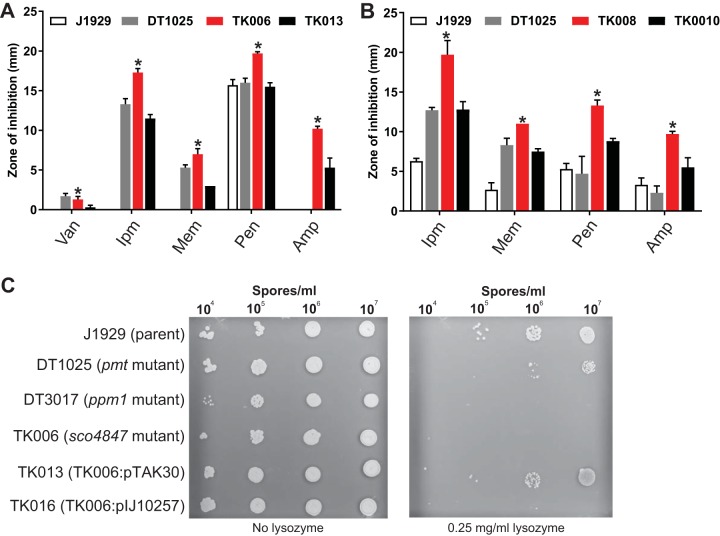
Antibiotic sensitivities of glycoprotein-deficient mutants. (A and B) Diameters of growth inhibition zones from disc diffusion assays for the S. coelicolor glycoprotein-deficient mutants TK006 (*sco4847*::Tn*5062*) (A) and TK008 (*sco4934*::Tn*5062*) (B) and respective complement strains TK013 (*sco4847*::Tn*5062*, pTAK30) and TK010 (*sco4934*::*T*n*5062*, pTAK32) against the parent strain J1929 and the glycosylation-deficient strain DT1025 (*pmt* mutant). Means from three biological replicates are shown, except for TK006, where the means from two biological replicates and three technical replicates are shown. Error bars indicate standard errors of the means. An asterisk indicates a *P* value of <0.05, i.e., that the difference between the glycoprotein-deficient mutant and the parent strain J1929 has occurred by chance. Only a selection of antibiotic concentrations (vancomycin, 40 μg; imipenem, 4 μg; meropenem, 4 μg; penicillin, 100 μg; ampicillin, 200 μg) are shown here; the full set is in Data Set S3. (C) Lysozyme sensitivity of TK006 (*sco4847*::Tn*5062*) and complement strain TK013 (*sco4847*::Tn*5062*, pTAK30) compared to the parent strain J1929, DT1025 (*pmt* mutant), and DT3017 (*ppm1* mutant). Images are representative of two biological replicates and two technical replicates.

To investigate the putative roles of glycoproteins SCO4847 and SCO4934 in cell wall biosynthesis, *sco4847* and *sco4934* were disrupted in S. coelicolor by allelic exchange with cosmids containing Tn*5062* in the gene of interest. The susceptibilities of the *sco4847* (TK006) and *sco4934* (TK008) mutant strains to a range of antibiotics were measured (Fig. 3). Both *sco4847* (TK006) and *sco4934* (TK008) mutants were significantly more susceptible to the β-lactam antibiotics imipenem, meropenem, ampicillin, and penicillin than the S. coelicolor parent strain J1929 (Fig. 3A and B). Additionally, *sco4847* (TK006) mutants displayed a slight increase in sensitivity to vancomycin compared to that of J1929 (Fig. 3A). Both mutants were more sensitive to the antibiotics than DT1025 (*pmt* mutant), suggesting that the nonglycosylated SCO4847 and SCO4934 isoforms still have some activity in DT1025. The increased antibiotic susceptibility was partially complemented upon the reintroduction of the wild-type copies of *sco4847* and *sco4934*, respectively. Neither of the mutants displayed any change in susceptibility to rifampin, bacitracin, or teicoplanin (Data Set S3), suggesting that the mutants were only affected by antibiotics that targeted peptidoglycan cross-linking. To further investigate the roles of SCO4847 and SCO4934 in cell wall biosynthesis, the susceptibility of the *sco4847* (TK006) and *sco4934* (TK008) mutants to lysozyme was tested. The *sco4847* (TK006) mutant was more sensitive to lysozyme treatment than J1929 and DT1025 (*pmt* mutant), and a wild-type level of lysozyme sensitivity was restored in the complemented strain (TK013) (Fig. 3C). No change in lysozyme sensitivity was observed in the *sco4934* (TK008) mutant (Fig. S4). Neither of the mutants displayed any changes in colony morphology, sporulation, or φC31*cΔ25* phage sensitivity (data not shown). The increase in susceptibility to cell wall-targeting antibiotics in the glycoprotein-deficient mutants suggests that both proteins are required for maintaining normal cell wall integrity in S. coelicolor. The lack of sensitivity to lysozyme observed in the *sco4934* mutant may be due to the compensatory actions of other l,d-transpeptidases in the cell. A BLAST search of the SCO4934 protein sequence against the StrepDB revealed at least three other putative l,d-transpeptidases in the S. coelicolor genome (SCO3194, SCO5458, and SCO5457). The increased lysozyme susceptibility observed in the *sco4847* (TK006) mutant suggests that SCO4847 has a very specific role in cell wall biosynthesis of S. coelicolor or is required during a specific growth stage.

10.1128/mBio.01092-19.6 FIG S4 Lysozyme sensitivity of TK008 (*sco4934* mutant) compared to the parent strain J1929, DT1025 (*pmt* mutant), and DT3017 (*ppm1* mutant). Download FIG S4, DOCX file, 0.7 MB. Copyright © 2019 Keenan et al. 2019 Keenan et al. This content is distributed under the terms of the Creative Commons Attribution 4.0 International license.

10.1128/mBio.01092-19.9 DATA SET S3 Antibiotic disc diffusion assay data. Download Data Set S3, XLSX file, 0.1 MB. Copyright © 2019 Keenan et al. 2019 Keenan et al. This content is distributed under the terms of the Creative Commons Attribution 4.0 International license.

### Conclusions.

In this study, we have combined biochemical and MS-based approaches to isolate and characterize the membrane O-glycoproteome in S. coelicolor. Collectively we have identified thirty-seven new S. coelicolor glycoproteins and further characterized the previously identified glycoprotein, PstS (15). As in M. tuberculosis (12, 14), S. coelicolor glycosylates a large number of proteins with a wide range of biological functions, including solute binding, polysaccharide hydrolases, ABC transporters, and cell wall biosynthesis. Glycosylation sites were found to be modified with up to three hexose residues, which is consistent with what has been seen previously in other *Actinobacteria* (10, 13, 14). The identification of glycoproteins with putative roles in cell wall biogenesis supports our hypothesis that glycoproteins in S. coelicolor are required for maintaining cell wall integrity. Upon further investigation of two of these glycoproteins, a putative d-Ala-d-Ala carboxypeptidase (SCO4847) and an l,d-transpeptidase (SCO4934), through the generation of null mutants we were able to reproduce the antibiotic susceptibility phenotype observed previously in the S. coelicolor *pmt* mutants (6). Additionally, the *sco4847* mutants displayed an increased susceptibility to lysozyme treatment. These findings strongly suggest that both glycoproteins are required for maintaining cell wall integrity and that glycosylation affects enzyme function.

## MATERIALS AND METHODS

### Bacterial strains, plasmids, and growth conditions.

Bacterial strains, plasmids, cosmids, and primers used in this work are listed in Table S1 in the supplemental material. Escherichia coli strains were grown in LB or on LB agar. Streptomyces coelicolor A3 (2) strains were maintained on solid soya flour mannitol (SFM) medium, from which spores were harvested and kept frozen in 20% glycerol at −38°C (40). For the preparation of mycelium from liquid cultures, pregerminated spores (40) were inoculated into F134 medium (19) to an optical density at 450 nm (OD_450_) of 0.03 to 0.05 and grown at 30°C with shaking (180 rpm) for up to 60 h. E. coli DH5α was used as a cloning host, and plasmids/cosmids were introduced into S. coelicolor by conjugation from the donor E. coli strain ET12567(pUZ8002) (40, 41). Apramycin (cosmids) or hygromycin (complementation plasmids) was used to select for exconjugates, and nalidixic acid was used to prevent growth of the E. coli donors. S. coelicolor strains containing a Tn*5062* insertion in the desired gene in the chromosome were obtained by screening exconjugants for those that had undergone double crossovers with the incoming cosmids and were apramycin resistant and kanamycin sensitive. Tn*5062* insertion mutants and complemented strains were validated by PCR and Southern blotting.

10.1128/mBio.01092-19.1 TABLE S1 Bacterial strains, cosmids, plasmids, and primers used in this work. Download Table S1, DOCX file, 0.02 MB. Copyright © 2019 Keenan et al. 2019 Keenan et al. This content is distributed under the terms of the Creative Commons Attribution 4.0 International license.

### Construction of the complementation plasmids.

For the construction of the *sco4934* complementation plasmid pTAK32, the sco4934 coding sequence was amplified by PCR from S. coelicolor J1929 genomic DNA using primers TK101 and TK102 (Table S1) and cloned into NdeI-digested pIJ10257. For the construction of the *sco4847* complementation plasmid pTAK30, the *sco4847* coding sequence could not be amplified by PCR from S. coelicolor J1929 genomic DNA, as it contained several sequence repeats. To simplify the template for PCR, the cosmid St5G8 was restricted with BamHI and separated by agarose gel electrophoresis, and a 2,270-bp product containing the *sco4847* coding sequence was excised and gel extracted. The purified DNA was used as a template for the amplification of *sco4847* by PCR with primers TK97 and TK98 (Table S1). The resulting PCR product was cloned into NdeI-digested pIJ10257. All plasmids were validated by DNA sequencing.

### Antibiotic disc diffusion assays.

Antibiotic disc diffusion assays were performed as described previously (6). Briefly, Difco nutrient agar plates were overlaid with soft nutrient agar (2.5 ml) containing ∼10^7^ S. coelicolor spores. Sterile filter discs (5-mm width) were placed on the surface of the soft agar, and 5 μl of antibiotic stock solution was allowed to absorb to the disc. Plates were incubated at 30°C for 2 days and zones of inhibition (measured in millimeters) were recorded.

### Lysozyme sensitivity assays.

Lysozyme sensitivity assays were performed by plating 5 μl of a dilution series of S. coelicolor spores (10^8^ to 10^4^ spores/ml in double-distilled H_2_O) onto Difco nutrient agar plates with and without lysozyme (0.25 mg/ml) and incubated at 30°C for 60 h.

### Preparation of S. coelicolor membrane proteins.

S. coelicolor membrane proteins were isolated as previously described (15). Briefly, the mycelium from liquid cultures was harvested by centrifugation (5 min, 3,500 × *g*, 4°C) and washed in 20 mM Tris-HCl buffer (pH 8, 4°C). Mycelial pellets were resuspended in twice the pellet volume of lysis buffer at 4°C (20 mM Tris-HCl, pH 8, 4 mM MgCl_2_, protease inhibitor tablet according to volume [Roche] and 1 unit mlˉ^1^ Benzonase [Sigma]). The mycelium was lysed using a manual French press (Thermo Fisher Scientific) at 25,000 lb/in^2^ kPsi. Cell debris was removed by centrifugation (30 min, 5,525 × *g*, followed by 30 min at 12,000 to 15,000 × *g*, 4°C). Membranes in the supernatant were pelleted by ultracentrifugation (1 h, 100,000 × *g*, 4°C). Membrane pellets were solubilized overnight on ice in 1% (wt/vol) dodecyl-β-d-maltoside (Sigma) in 20 mM Tris-HCl buffer (pH 8).

### SDS-PAGE and lectin Western blotting.

Protein concentrations were determined using the Pierce Coomassie (Bradford) assay kit (Thermo Fisher Scientific). Proteins were prepared by boiling in 1× RunBlue LDS sample buffer (Expedeon) with β-mercaptoethanol (5% [vol/vol]) and separated in RunBlue 4 to 12% SDS protein gels (Expedeon). For protein staining, gels were soaked in InstantBlue protein stain (Expedeon) per the manufacturer’s instructions. For glycoprotein detection, proteins were transferred to PVDF membranes by semidry Western transfer (42). Nonspecific binding to the membranes was blocked by incubation in TBS (50 mM Tris-HCl, 150 mM NaCl, pH 7.5) plus 2% (vol/vol) Tween 20 for 30 min, before washing the membranes 2 × 5 min in TBS. Membranes were incubated for 2 h in TBS plus 0.05% (vol/vol) Tween 20, 1 mM MgCl_2_, 1 mM MnCl_2_, and 1 mM CaCl_2_ with 5 μg·ml^−1^ ConA-HRP conjugate (Sigma). For the inhibition of glycoprotein binding, membranes were incubated for 2 h in TBS plus 0.05% (vol/vol) Tween 20, 1 mM MgCl_2_, 1 mM MnCl_2_, and 1 mM CaCl_2_ with 5 μg·mlˉ^1^ ConA-HRP conjugate and 200 mM methyl α-d-glucopyranoside. The membranes were washed twice for 10 min each time in TBS plus 0.05% (vol/vol) Tween 20 and once for 5 min in TBS. Chemiluminescent detection solution was prepared by adding 5 ml of 100 mM Tris-HCl, pH 8.5, buffer with 0.2 mM p-coumaric acid (Sigma) and 1.25 mM luminol to 15 μl of 3% (vol/vol) hydrogen peroxide solution. Under dark-room conditions, the membranes were incubated in chemiluminescent detection solution for 1 min. After exposure to the blot, X-ray film (GE Healthcare Life Sciences) was incubated for 3 to 5 min in Developer solution (Kodak) and 3 min in Fixer solution (Kodak).

### Lectin affinity chromatography.

Lectin affinity chromatography was performed on the AKTA pure chromatography system (GE Healthcare) using a column of agarose-bound ConA (Vector Laboratories). Prior to sample loading, the column was washed in lectin buffer (20 mM Tris-HCl, pH 7.5, 400 mM NaCl, 5 mM MgCl_2_, 5 mM MnCl_2_, and 5 mM CaCl_2_) and then equilibrated in 5× column volumes (CV) of binding buffer (20 mM Tris-HCl, pH 7.5, 0.4 M NaCl, and 0.1% [wt/vol] *n*-dodecyl β-d-maltoside). Samples were loaded onto the column at a flow rate of 5 ml·minˉ^1^, the column was washed with 16× CV of binding buffer, and glycoproteins were eluted in 4× CV of a 200 mM methyl α-d-glucopyranoside solution. Glycoprotein fractions were concentrated using Amicon ultracentrifugal filters (9-kDa molecular weight cutoff; Merck) and stored in 50% (wt/vol) glycerol at −80°C.

### Glycoproteomics.

For detailed glycoproteomics methods, please see Text S1 in the supplemental material. Glycoproteins were in-gel digested with trypsin before LC-MS/MS acquisition over 180 min using multiple fragmentation strategies. CID fragmentation acquisitions were performed using a Waters nanoAcquity ultraperformance liquid chromatograph interfaced to a Bruker maXis HD mass spectrometer as previously described (43). HCD, ETD, and mixed fragmentation acquisitions were performed using a Thermo UltiMate 3000 RSLCnano high-performance liquid chromatograph and Orbitrap Fusion hybrid mass spectrometer. Four MS/MS strategies were employed: ETD spectra acquired in the linear ion trap (ETD_IT), ETD spectra acquired in the Orbitrap (ETD_OT), HCD spectra acquired in the linear ion trap (HCD_IT), and HCD spectra acquired in the linear ion trap with ETD spectra acquired in the Orbitrap (HCD/ETD IC). Resulting tandem mass spectral data were searched against the Streptomyces coelicolor subset of the NCBI database using Mascot. Search criteria were the following: enzyme, trypsin; fixed modifications, carbamidomethyl (C); variable modifications, oxidation (M), deamidated (NQ), and Hex_(1–5)_ (ST). Mass tolerance and fragmentation ion types were adjusted to match acquisition dependencies (see the supplemental material). Peptide spectral matches were filtered to expect scores of ≤0.05. All glycopeptide spectra with MASCOT expect scores of 0.05 or lower were manually validated. For glycopeptide spectra generated by CID and HCD fragmentation, glycosylation sites were only assigned in cases where only a single glycosylated residue was possible within the glycopeptide. For the site localizations of glycopeptides identified in the ETD_IT and ETD_OT acquisitions, an MD score cutoff of 10 was applied. In matches where the MD score was greater than 10, the spectra were manually validated to confirm the site localization.

10.1128/mBio.01092-19.2 TEXT S1 Supplemental methods. Download Text S1, DOCX file, 0.04 MB. Copyright © 2019 Keenan et al. 2019 Keenan et al. This content is distributed under the terms of the Creative Commons Attribution 4.0 International license.

### Data availability.

All proteomics data are available through MassIVE as data set MSV000083115.
