# Supplementary material for: Characterization of the Streptomyces coelicolor Glycoproteome Reveals Glycoproteins Important for Cell Wall Biogenesis
Source: mBio. 2019 Jun 25;10(3):e01092-19. doi: 10.1128/mBio.01092-19 (PMC6593405; doi:10.1128/mBio.01092-19)
Supplement: FIG S1 [file mBio.01092-19-sf001.docx]

**
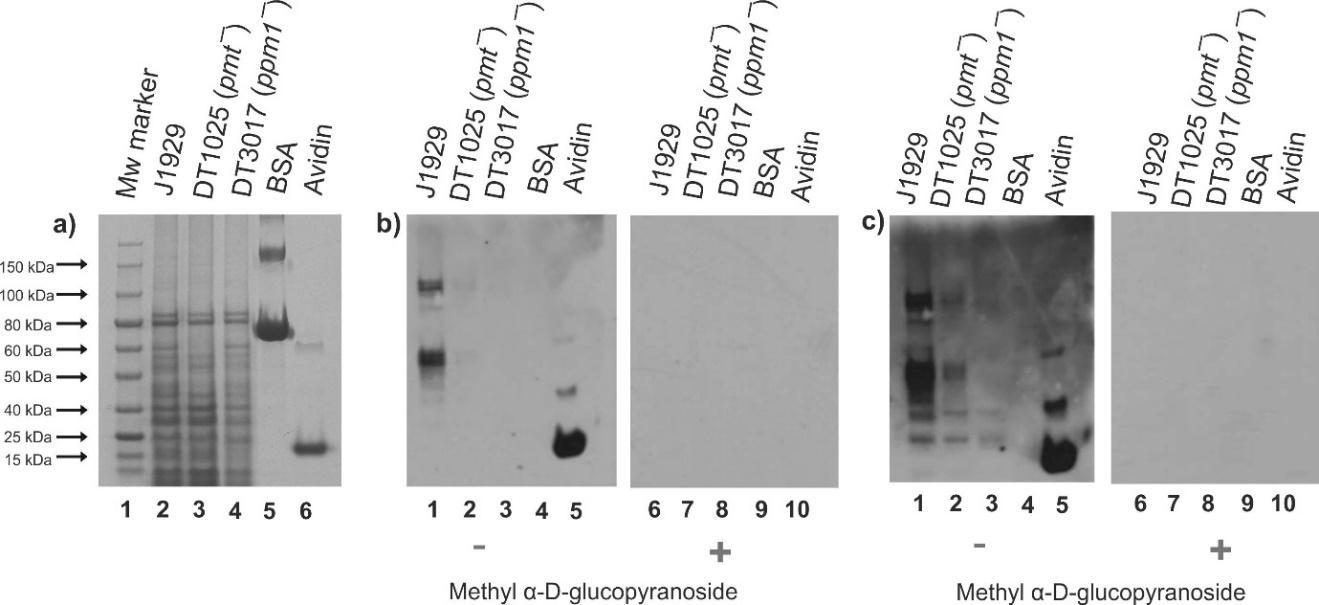
**

**Fig. S1. Detection of glycosylated proteins in the membrane proteome of S. coelicolor J1929 using Con A-HRP.** *S. coelicolor* J1929 and derivatives DT1025 (pmt¯) and DT3017 (ppm1¯) were grown in liquid culture for 25 h and the total membrane protein was isolated. Proteins were separated by SDS-PAGE and either stained with InstantBlue protein stain (a), or blotted onto PVDF membranes (b and c) and probed with Con A-HRP in the presence (lanes 1 - 5) and absence (lanes 6 - 10) of methyl α-D glucopyranoside. Protein loading was 17 µg for gels stained with InstantBlue protein stain and 5 µg for western blots probed with Con A-HRP. For the western blots probed with Con A-HRP, a 2 min (b) and 8 min (c) exposure to the membrane is shown. Bovine serum albumin (BSA) was a negative control and Avidin was a positive control for the Con A-HRP reactivity. The protein marker was the Broad range 10 – 250 kDa Mw marker (NEB).
