# Supplementary material for: Characterization of the Streptomyces coelicolor Glycoproteome Reveals Glycoproteins Important for Cell Wall Biogenesis
Source: mBio. 2019 Jun 25;10(3):e01092-19. doi: 10.1128/mBio.01092-19 (PMC6593405; doi:10.1128/mBio.01092-19)
Supplement: FIG S2 [file mBio.01092-19-sf002.docx]

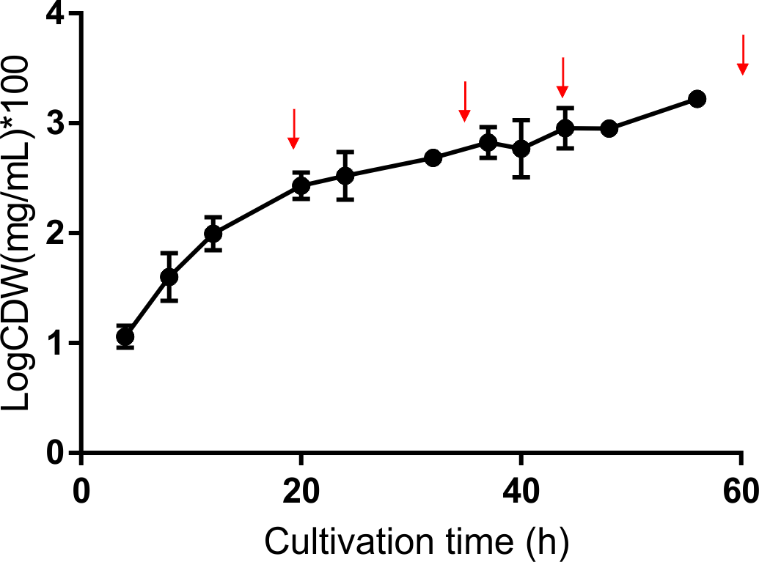


**Fig S2.** Growth of S. coelicolor J1929 in liquid F134 medium. S. coelicolor J1929 spores were germinated for 6 h and the cultures were grown for 56 h in F134 medium. Measurements of cell dry weight (CDW) were taken to monitor growth at regular intervals. The time points selected to harvest the cultures for glycoprotein isolation are indicated by red arrows. Error bars represent the standard error of the mean of three biological replicates.
