# Supplementary material for: Characterization of the Streptomyces coelicolor Glycoproteome Reveals Glycoproteins Important for Cell Wall Biogenesis
Source: mBio. 2019 Jun 25;10(3):e01092-19. doi: 10.1128/mBio.01092-19 (PMC6593405; doi:10.1128/mBio.01092-19)

Spectra generated by LC-ESI-CID-MS/MS on the Bruker maXis HD system

Key:

Ion Type	Description
b(++)	doubly charged ion series
b(*)	b - NH_3
b(0)	b - H_2O
y(++)	doubly charged ion series
y(*)	y - NH_3
y(0)	y - H_2O

Time point	60 hr
SCO number	SCO5115
Precursor ion mass	692.336
Charge	2
Retention time	103.6
Hex on peptide	1
e-value	8.90E-03
Site allocated?	Ser43

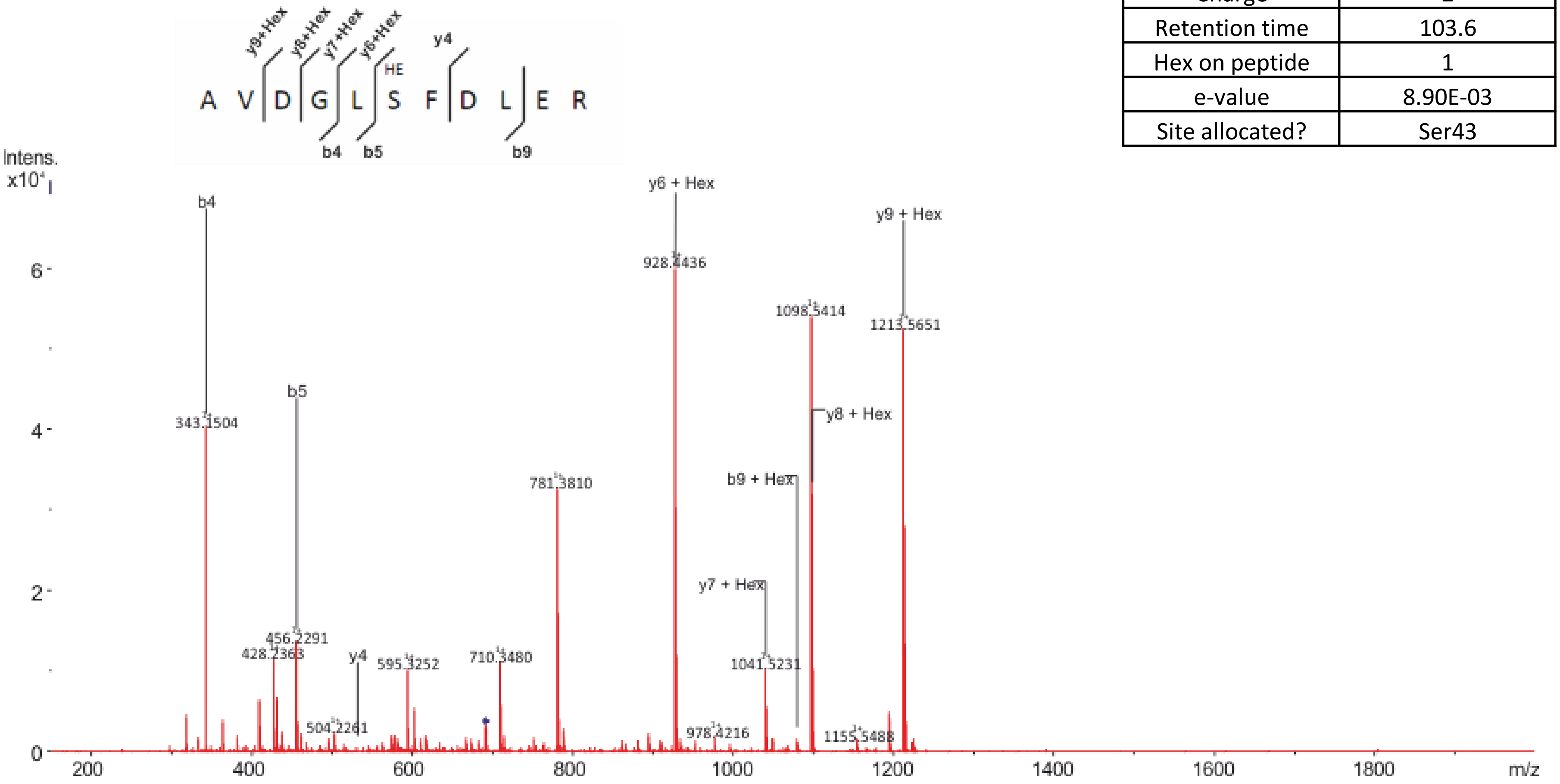

Time point	43 hr
SCO number	SCO6558
Precursor ion mass	559.797
Charge	2
Retention time	87.9
Hex on peptide	1
e-value	1.20E-02
Site allocated?	Thr104

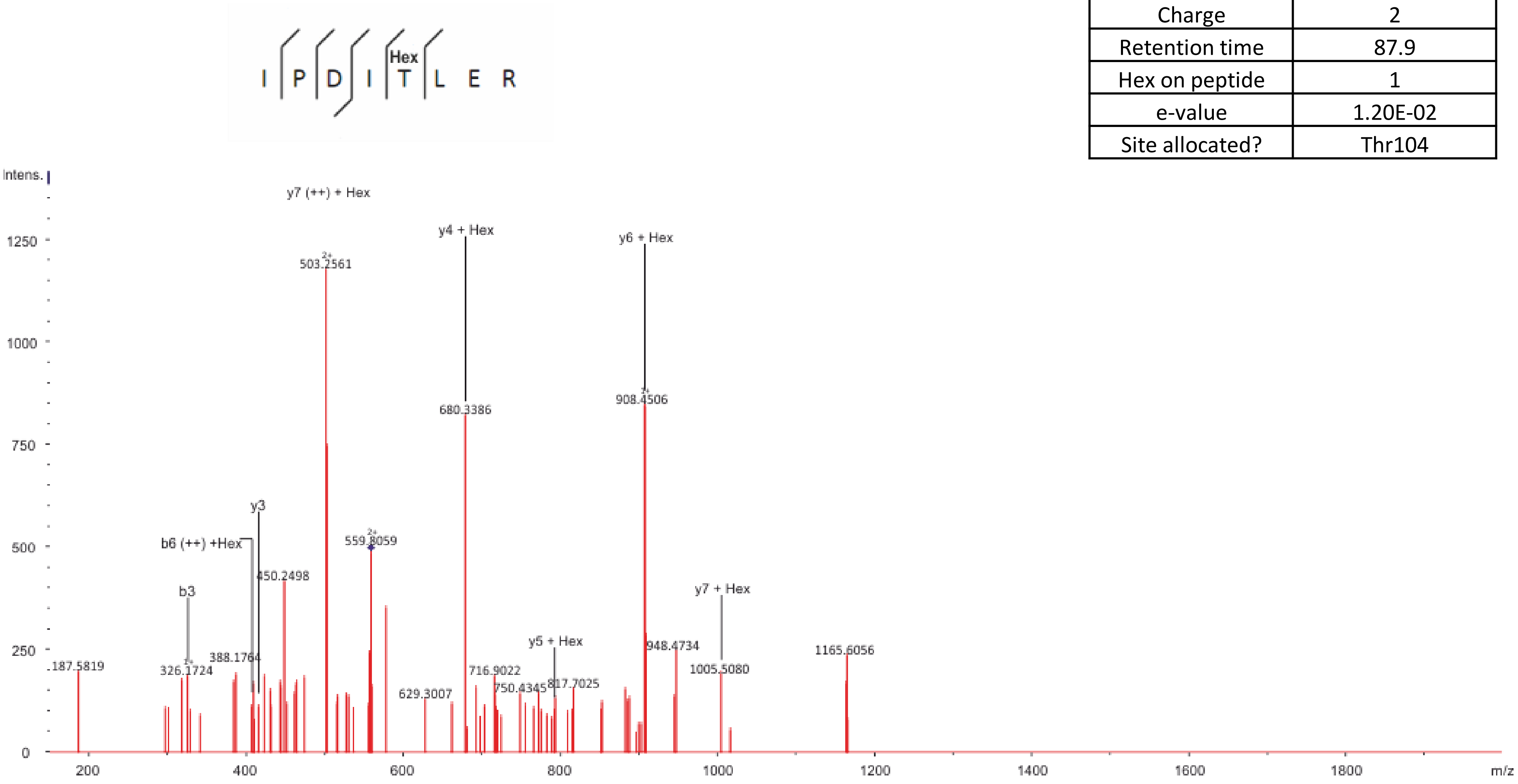

Time point	60 hr
SCO number	SCO5815
Precursor ion mass	910.981
Charge	2
Retention time	128.8
Hex on peptide	3
e-value	4.10E-02
Site allocated?	Ser228, Thr239

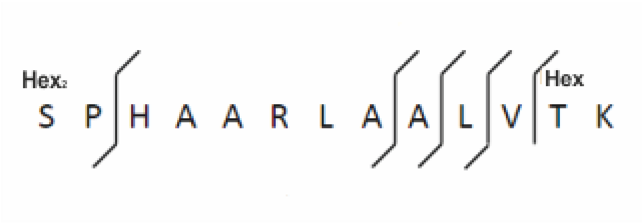

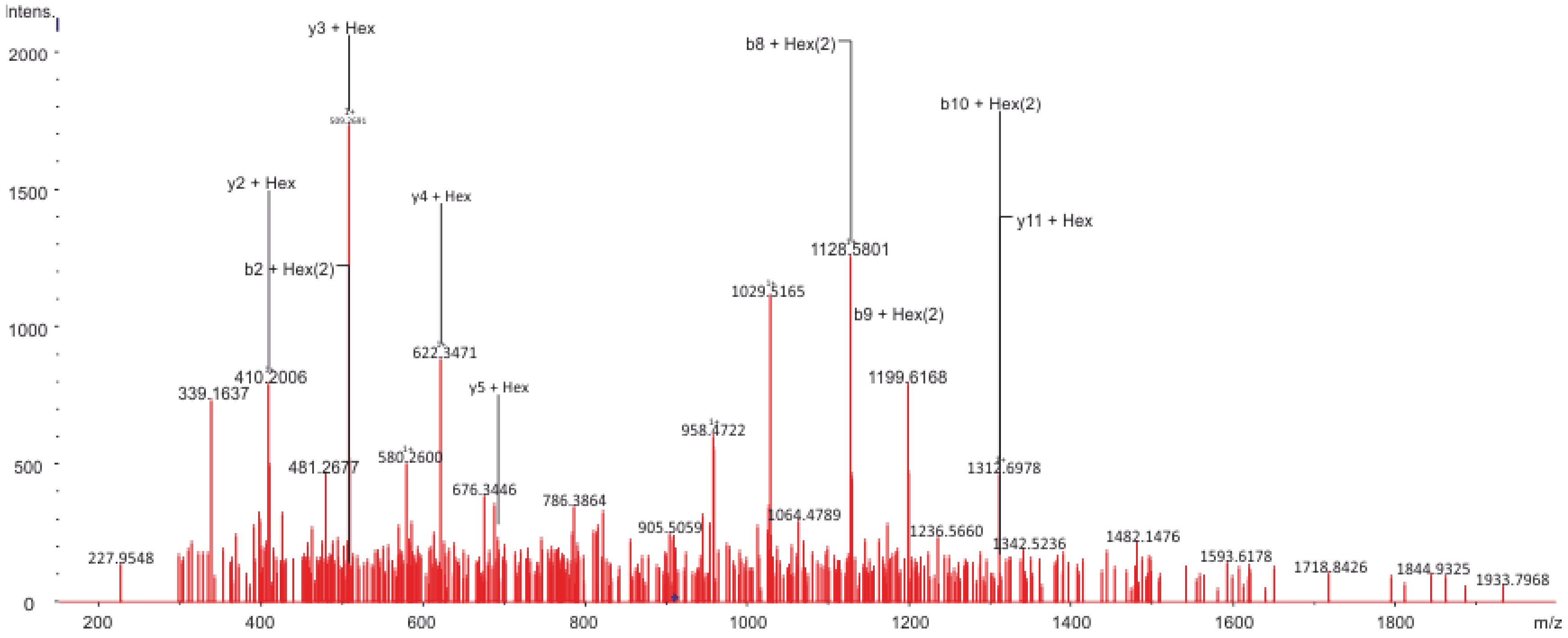

Time point	20 hr
SCO number	SCO5736
Precursor ion mass	910.450
Charge	2
Retention time	79.3
Hex on peptide	1
e-value	0.0005
Site allocated?	N

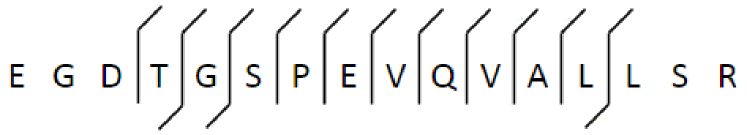

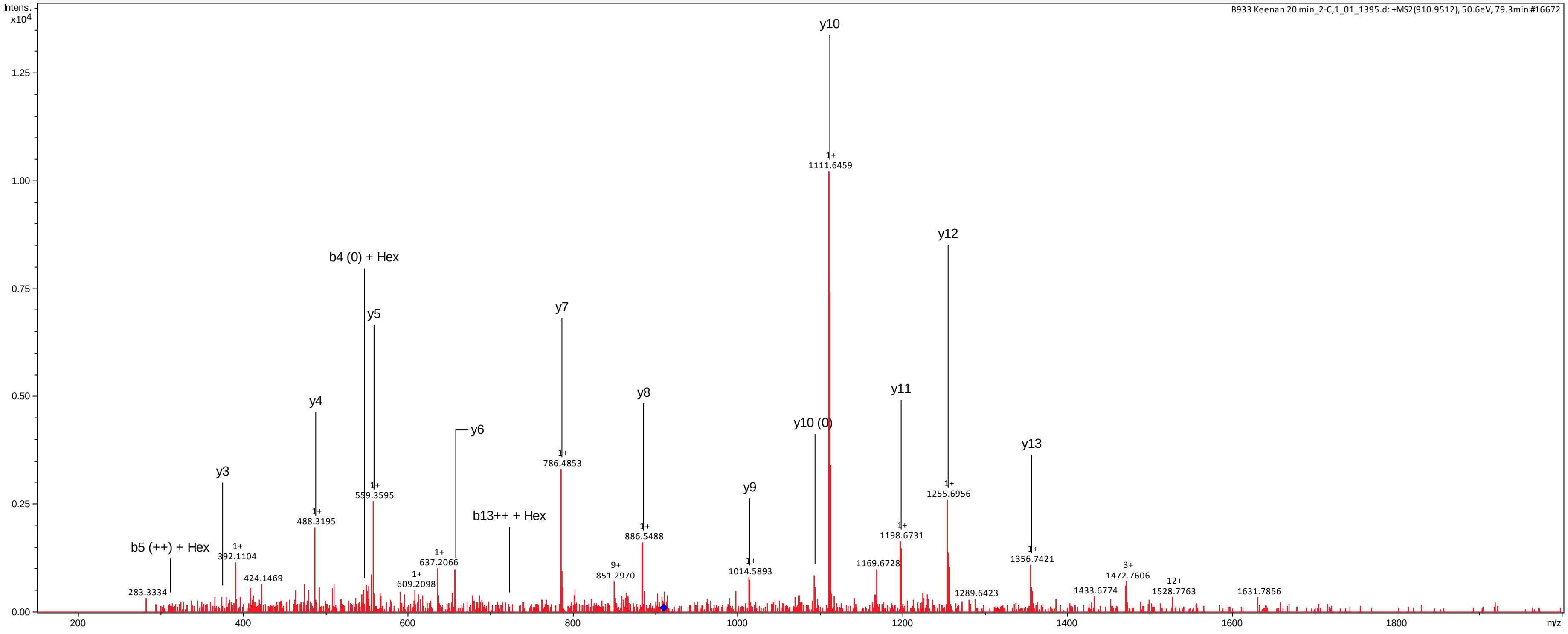

Time point	20 hr
SCO number	SCO4847
Precursor ion mass	1055.759
Charge	3
Retention time	59.5
Hex on peptide	9
e-value	0.00083
Site allocated?	N

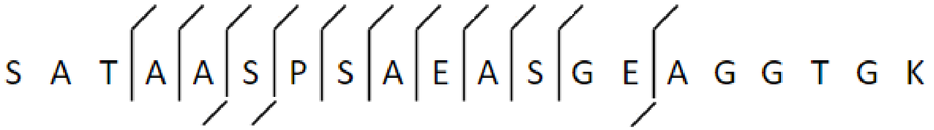

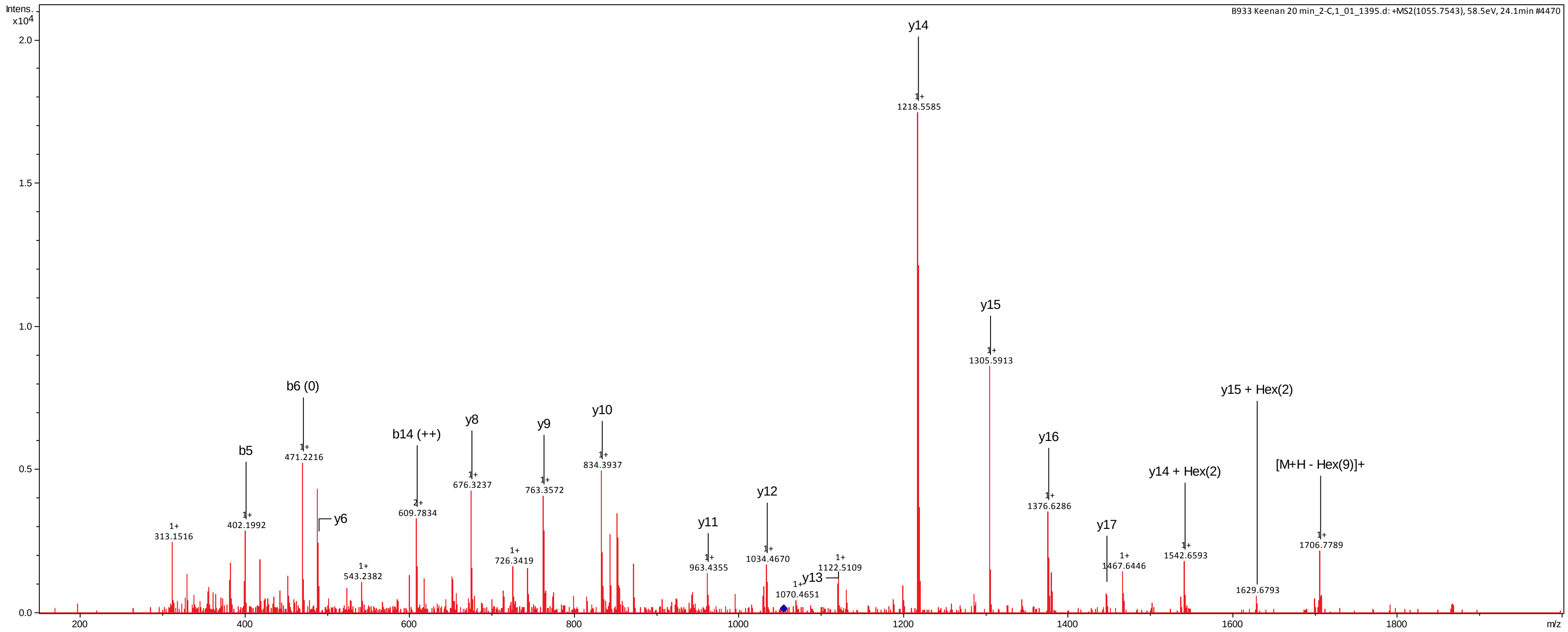

Time point	20 hr
SCO number	SCO4739
Precursor ion mass	1148.485
Charge	2
Retention time	25.1
Hex on peptide	4
e-value	0.0000041
Site allocated?	N

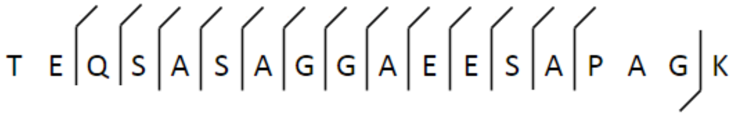

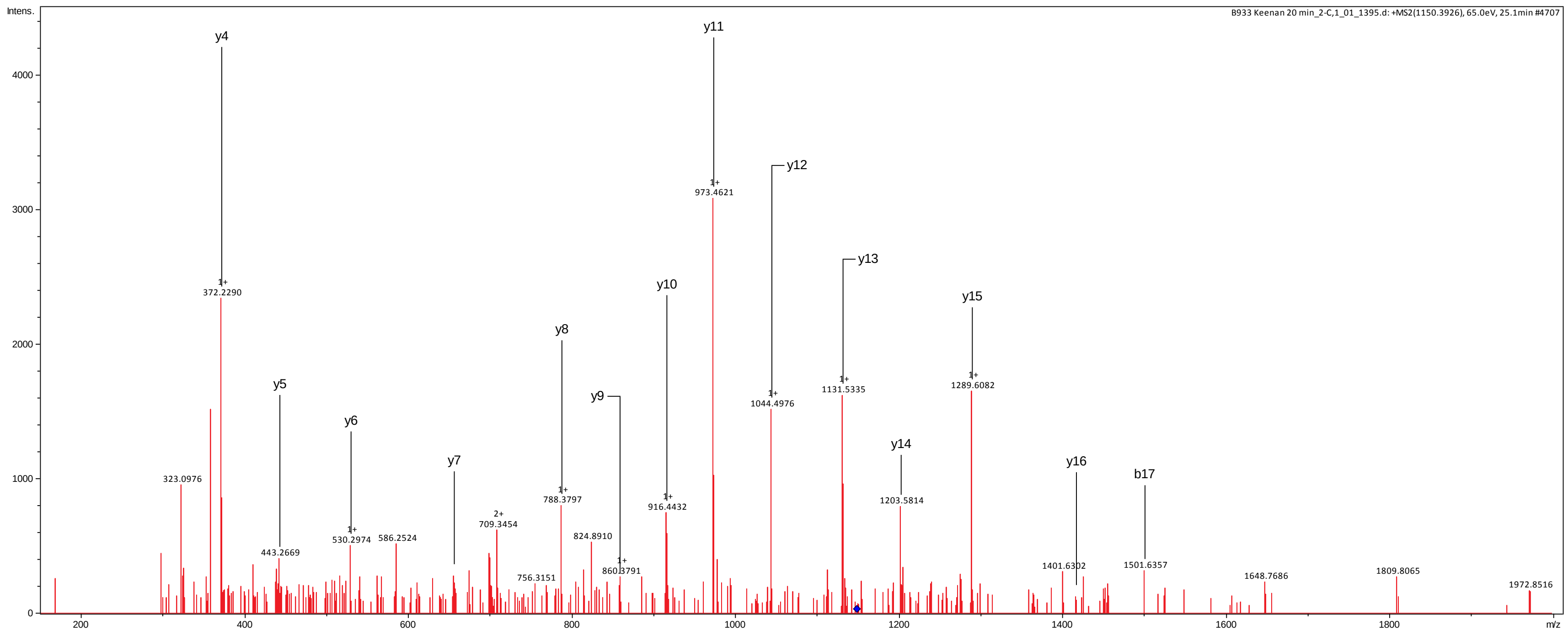

Time point	20 hr
SCO number	SCO4739
Precursor ion mass	1229.501
Charge	2
Retention time	24.8
Hex on peptide	5
e-value	0.0025
Site allocated?	N

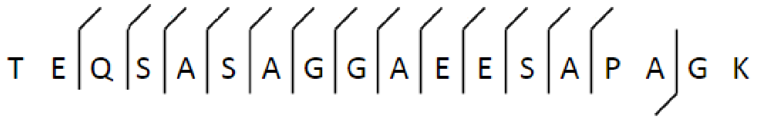

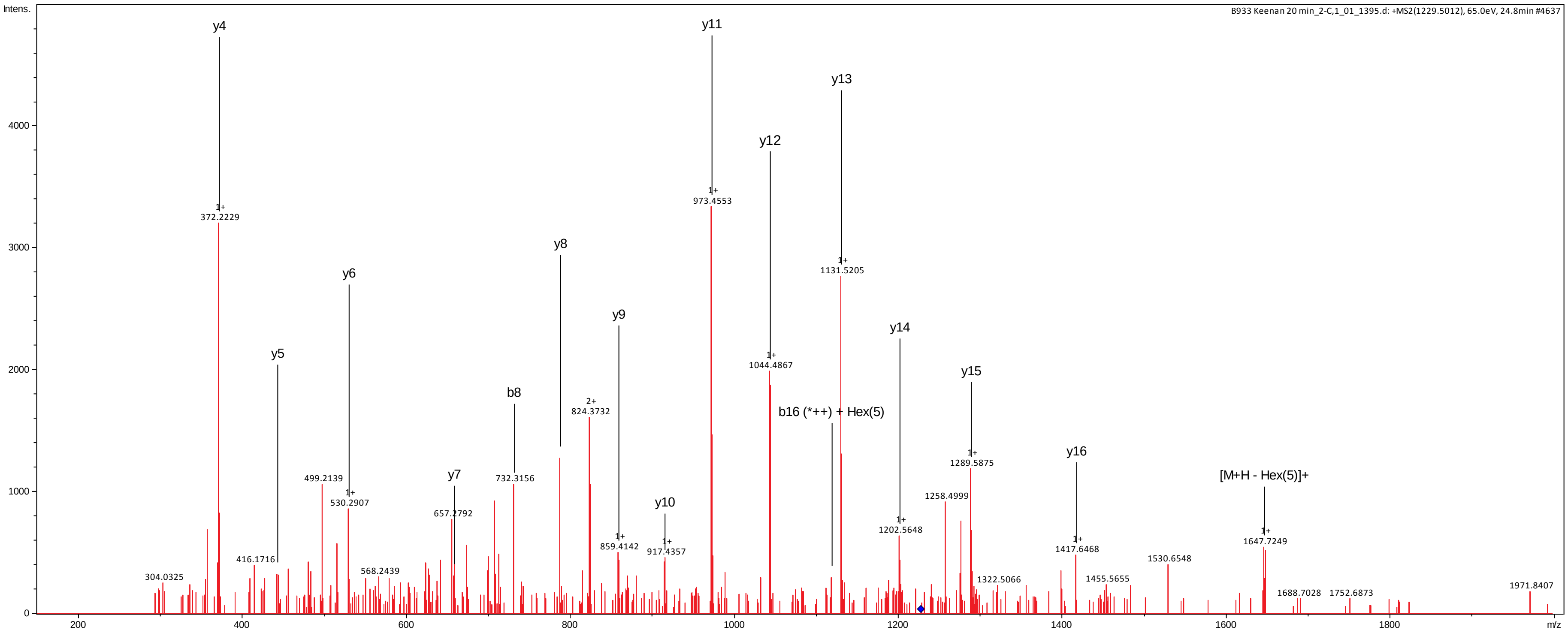

Time point	20 hr
SCO number	SCO4739
Precursor ion mass	874.023
Charge	3
Retention time	24.3
Hex on peptide	6
e-value	0.00025
Site allocated?	N

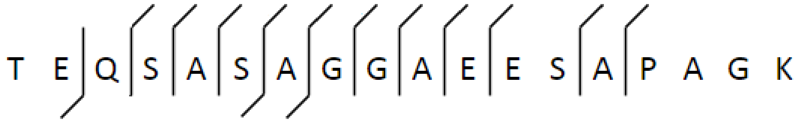

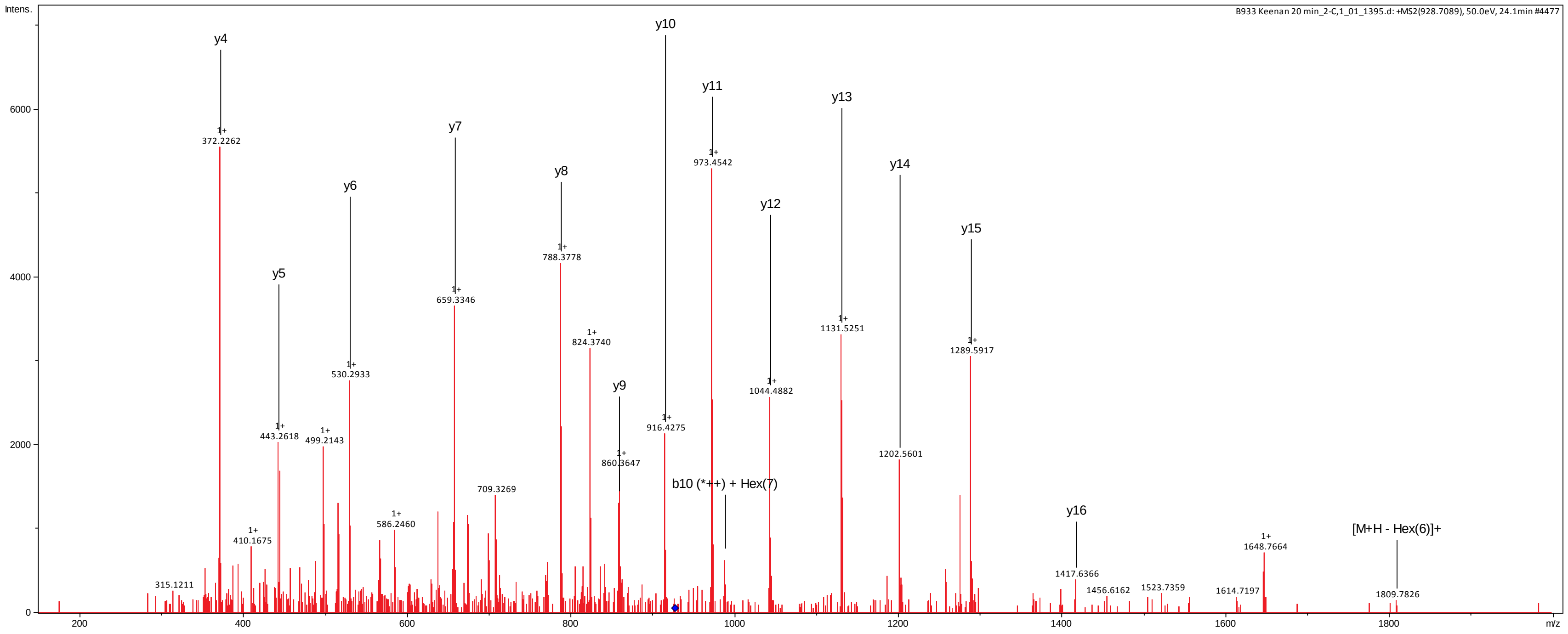

Time point	20 hr
SCO number	SCO4739
Precursor ion mass	928.040
Charge	3
Retention time	24.1
Hex on peptide	7
e-value	0.012
Site allocated?	N

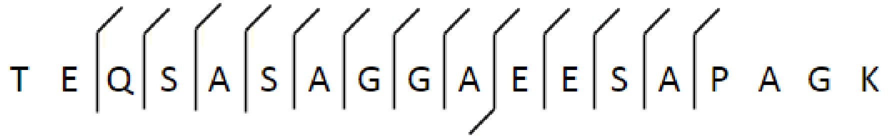

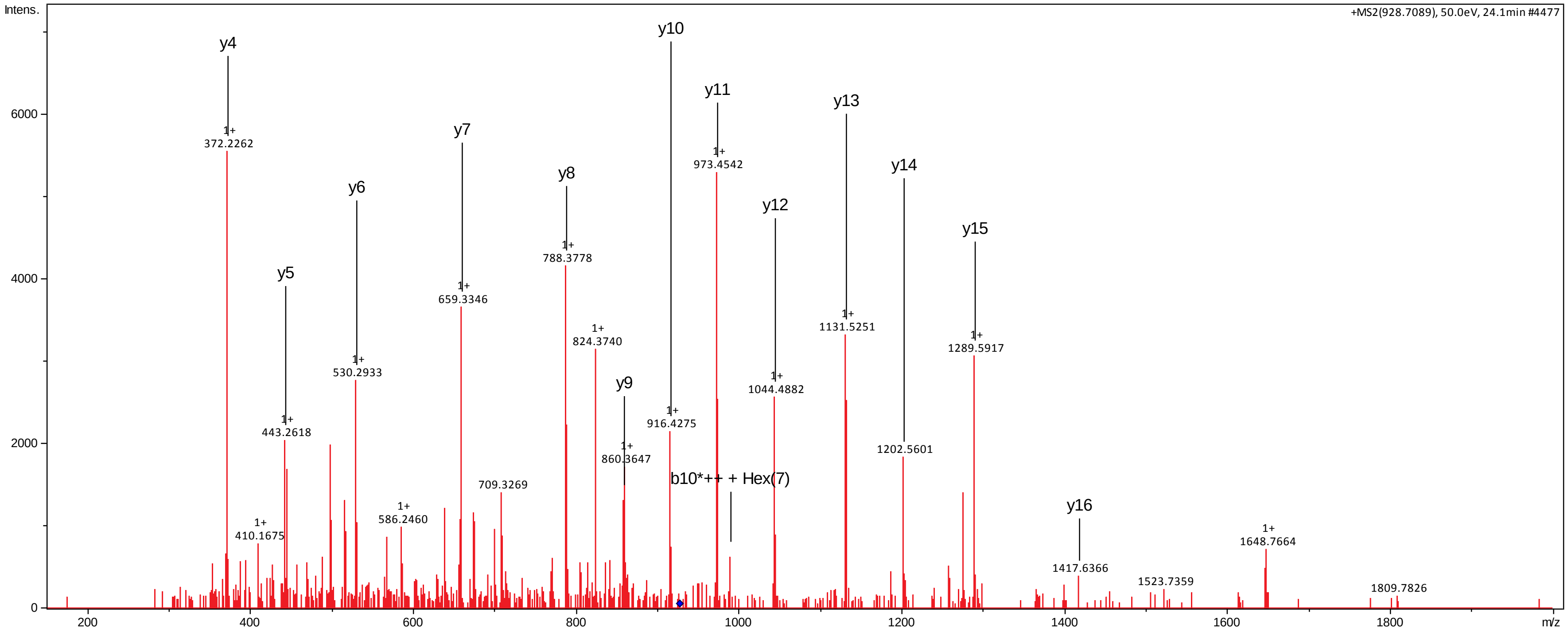

Time point	20 hr
SCO number	SCO4739
Precursor ion mass	982.060
Charge	3
Retention time	23.8
Hex on peptide	8
e-value	0.0018
Site allocated?	N

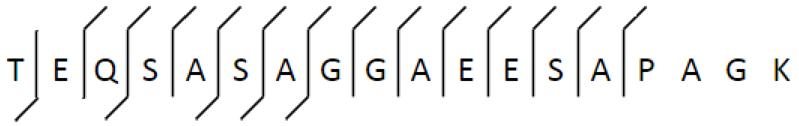

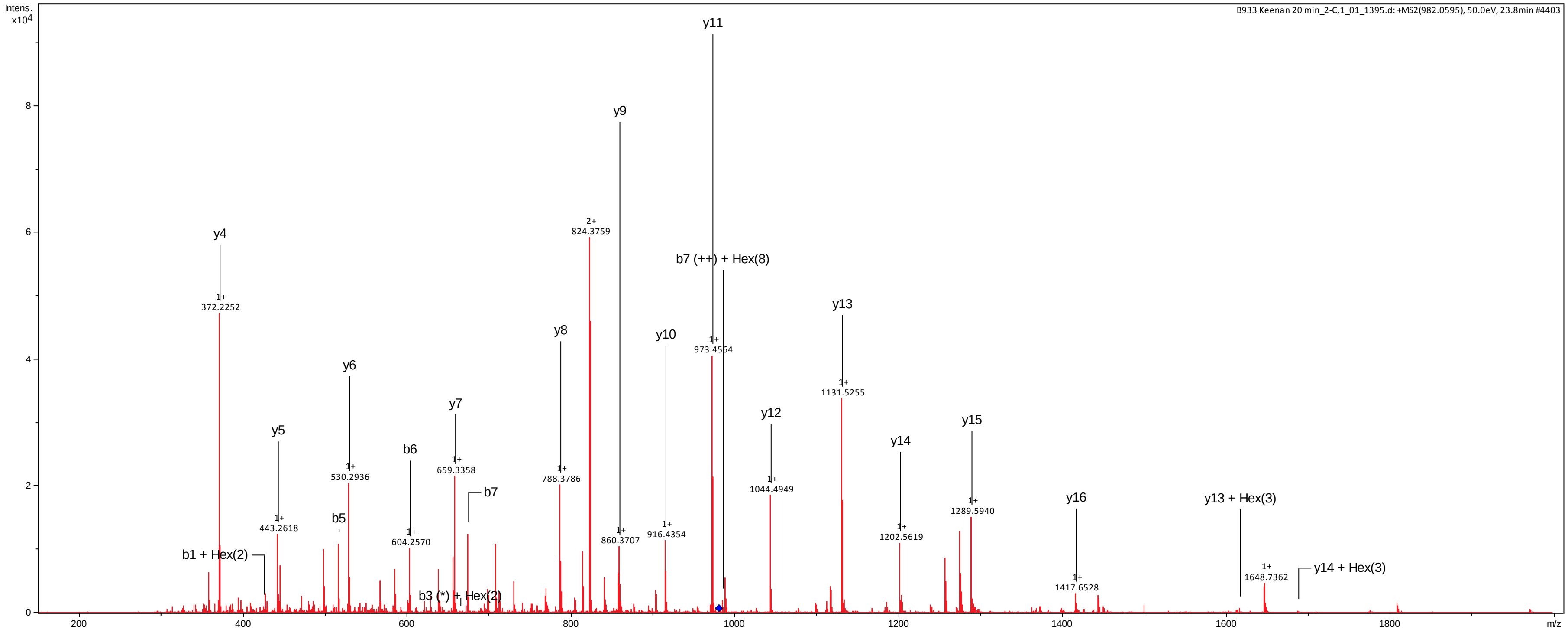

Time point	20 hr
SCO number	SCO4739
Precursor ion mass	1036.081
Charge	3
Retention time	23.6
Hex on peptide	9
e-value	0.00034
Site allocated?	N

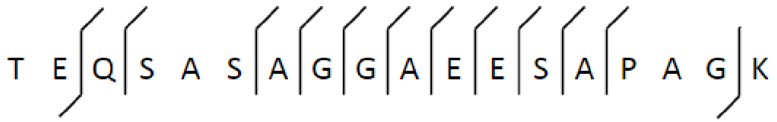

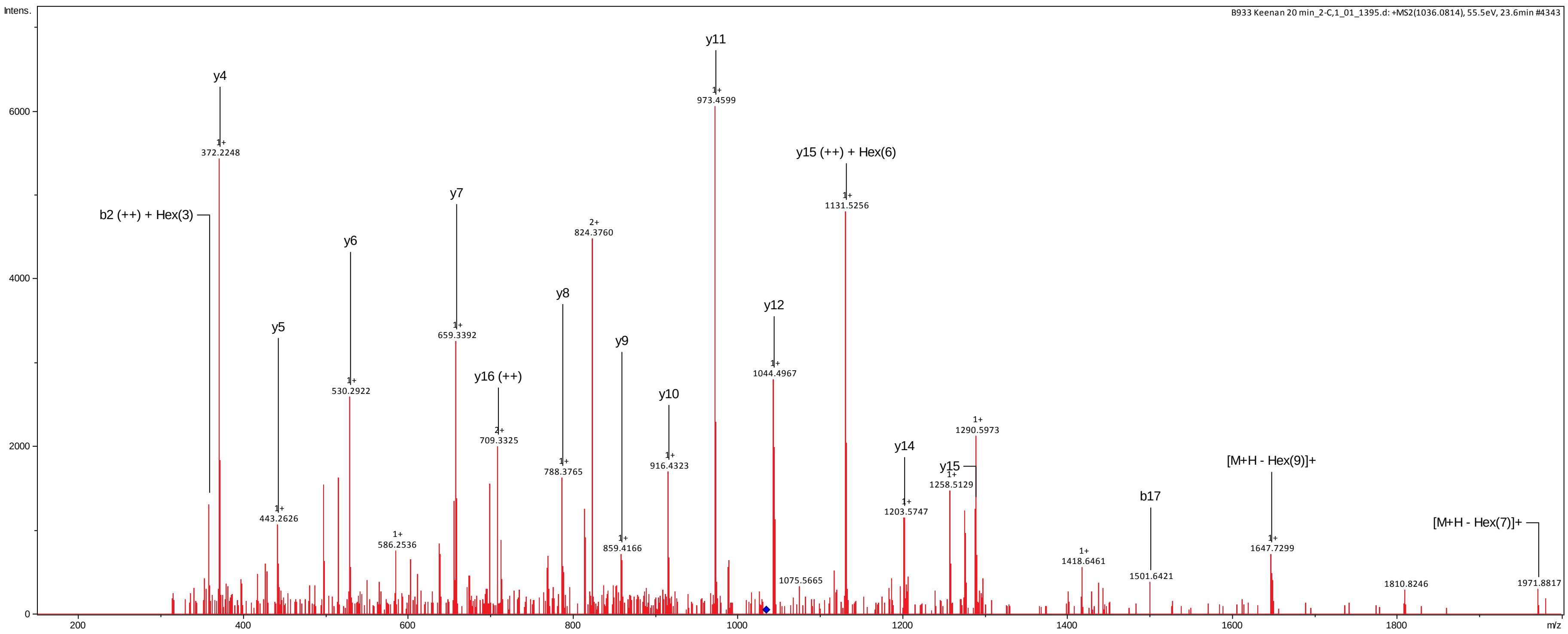

Time point	20 hr
SCO number	SCO0996
Precursor ion mass	1210.556
Charge	2
Retention time	75.7
Hex on peptide	3
e-value	0.00003
Site allocated?	N

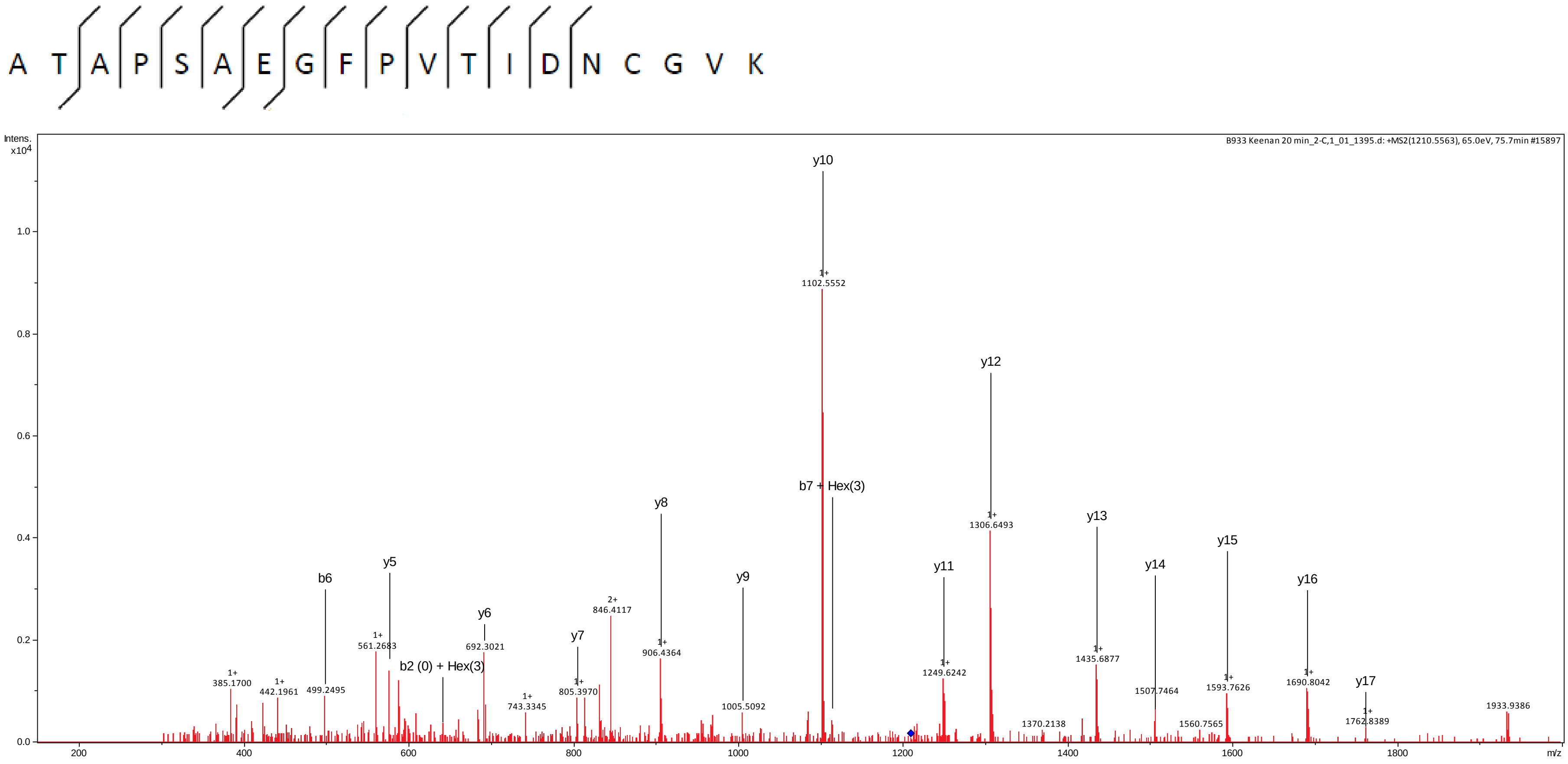

Time point	20 hr
SCO number	SCO4905
Precursor ion mass	919.788
Charge	3
Retention time	121.6
Hex on peptide	3
e-value	0.000000016
Site allocated?	N

A T P G L P A Q V F L L C G S S L V A V D R

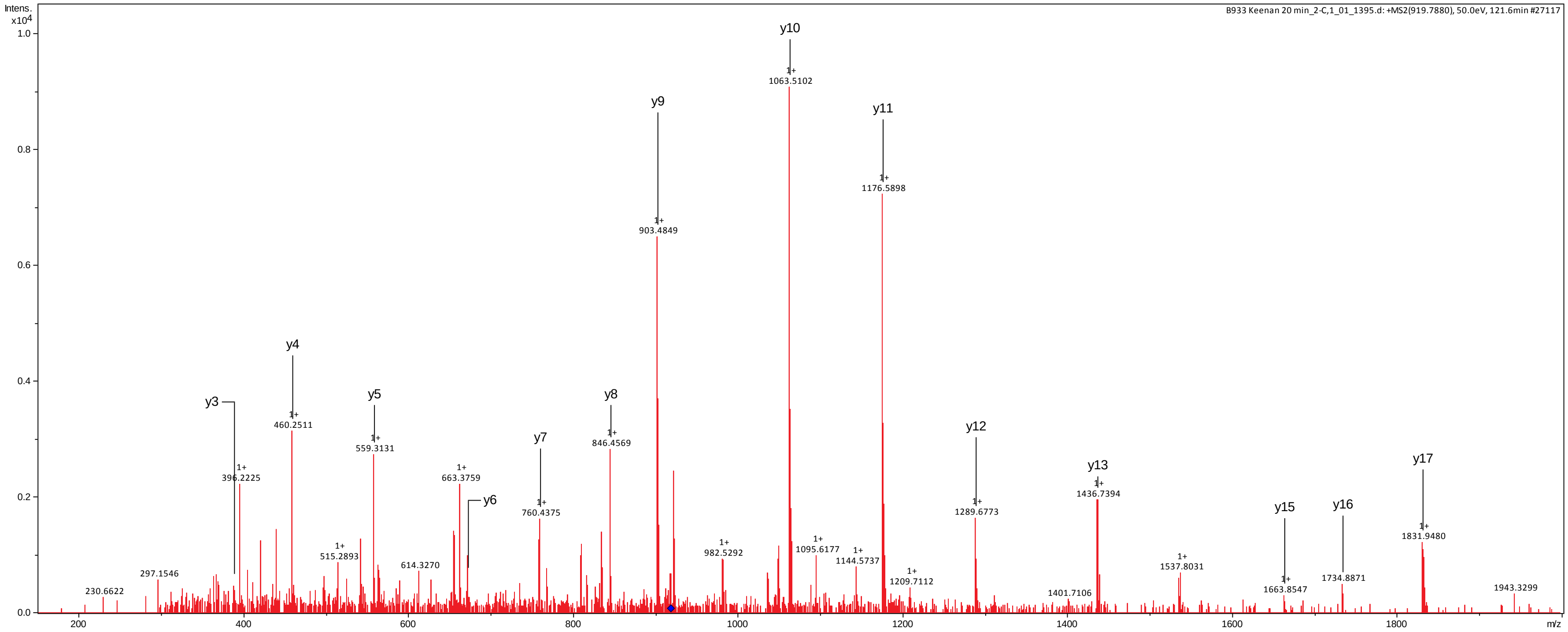

Time point	20 hr
SCO number	SCO4934
Precursor ion mass	853.378
Charge	2
Retention time	27.9
Hex on peptide	3
e-value	0.023
Site allocated?	N

T S Q A E V D E A A A K

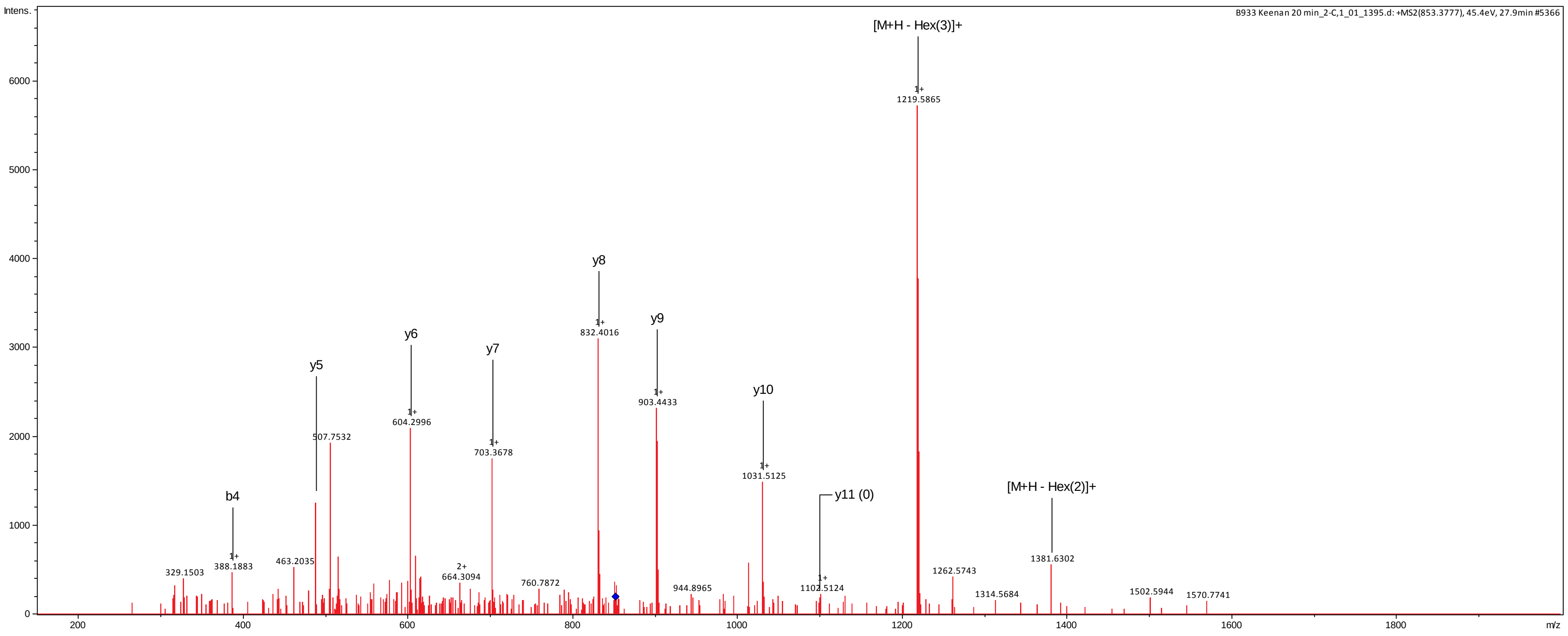

Time point	35 hr
SCO number	SCO4934
Precursor ion mass	772.341
Charge	2
Retention time	27.4
Hex on peptide	2
e-value	0.00019
Site allocated?	N

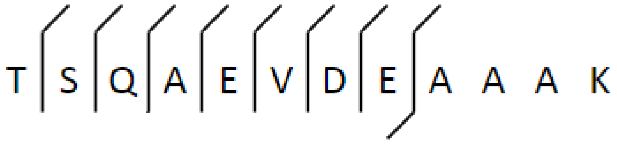

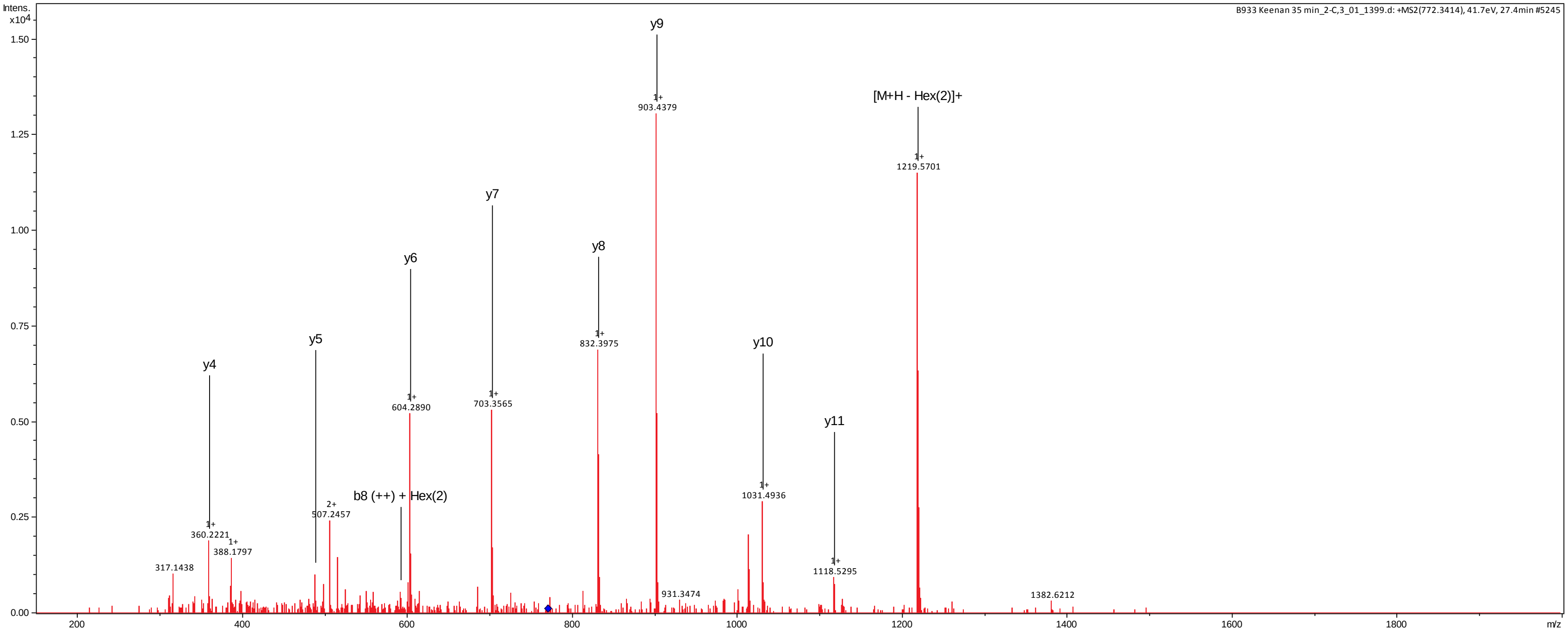

Time point	35 hr
SCO number	SCO4934
Precursor ion mass	853.368
Charge	2
Retention time	27
Hex on peptide	3
e-value	0.00024
Site allocated?	N

T S Q A E V D E A A A K

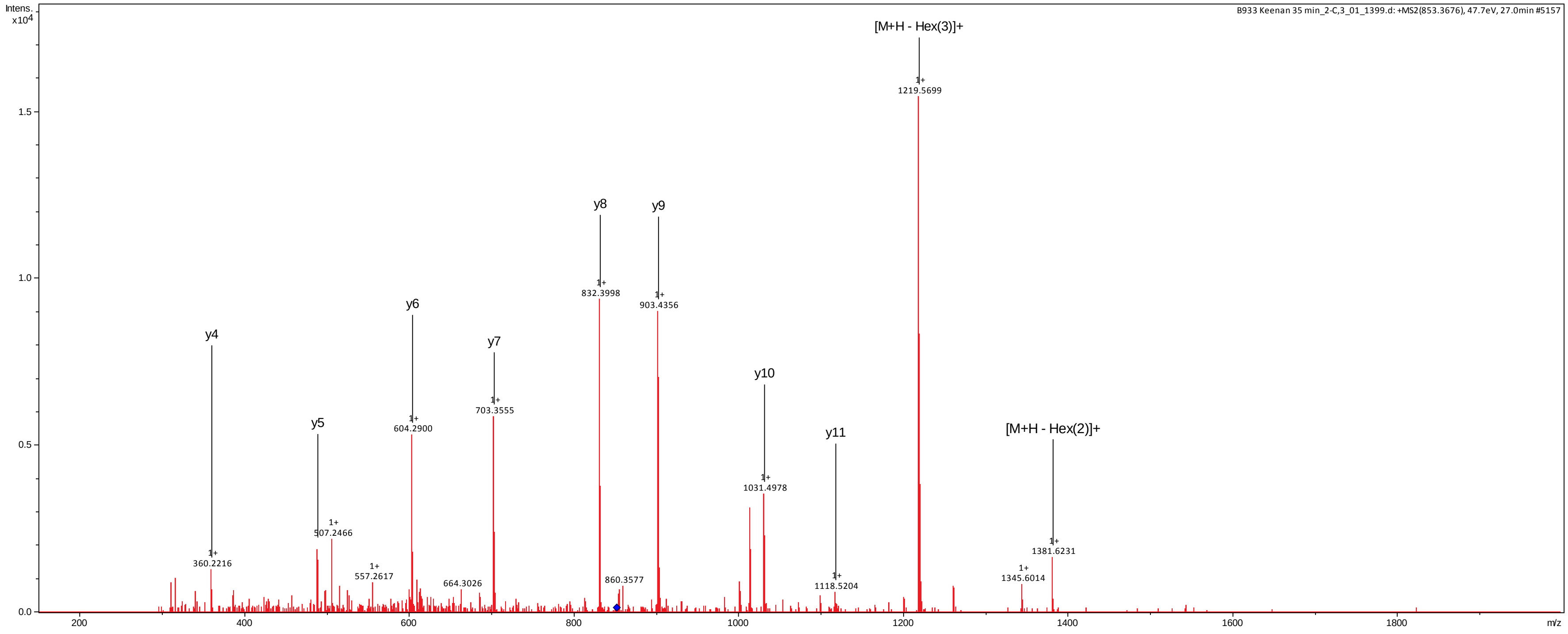

Time point	35 hr
SCO number	SCO4847
Precursor ion mass	1055.759
Charge	3
Retention time	59.5
Hex on peptide	9
e-value	0.0003
Site allocated?	N

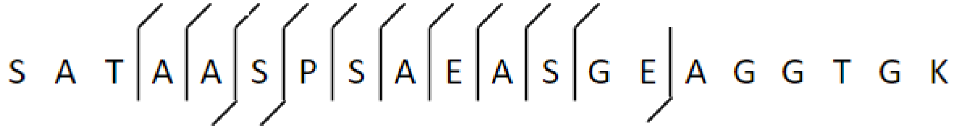

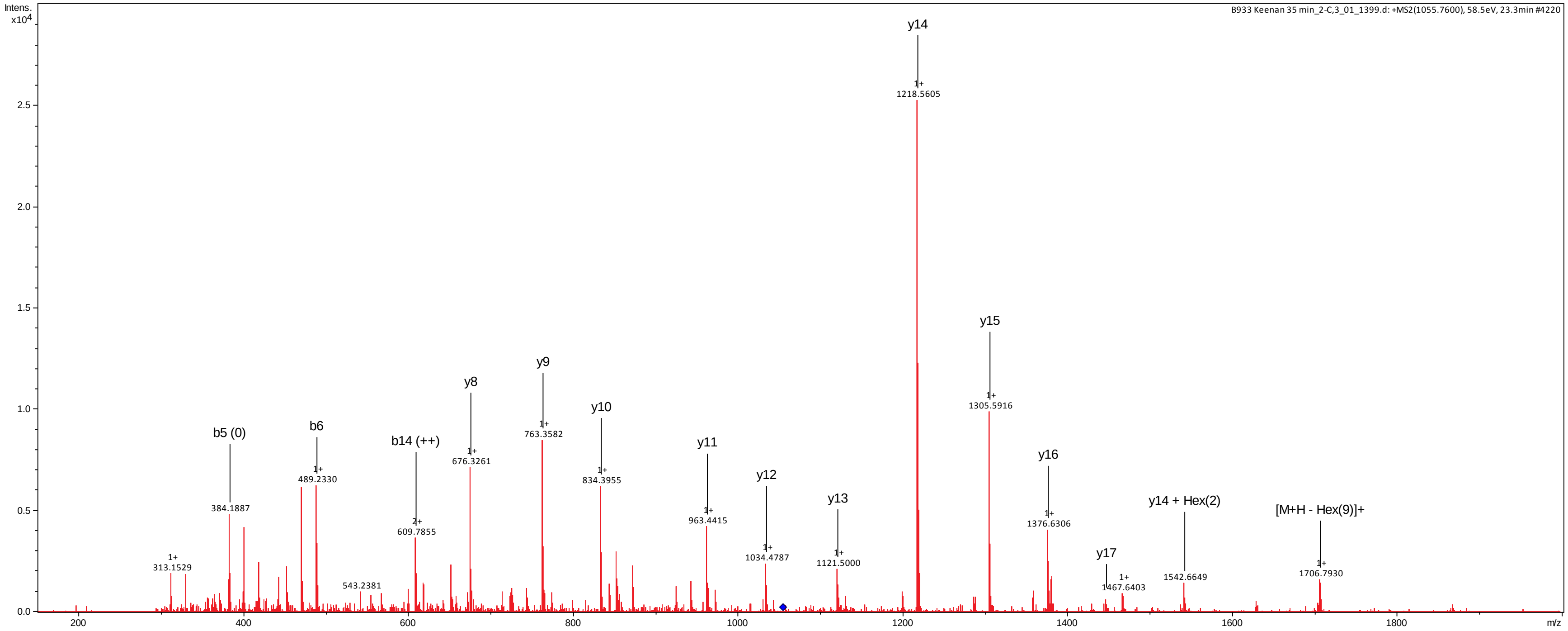

Time point	35 hr
SCO number	SCO4739
Precursor ion mass	982.063
Charge	3
Retention time	23.3
Hex on peptide	8
e-value	0.0045
Site allocated?	N

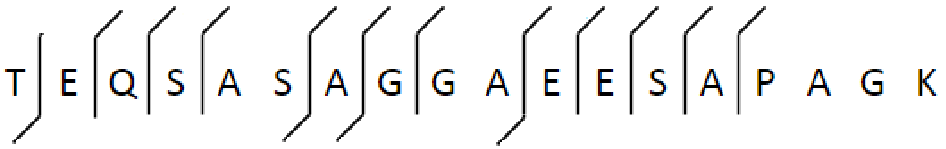

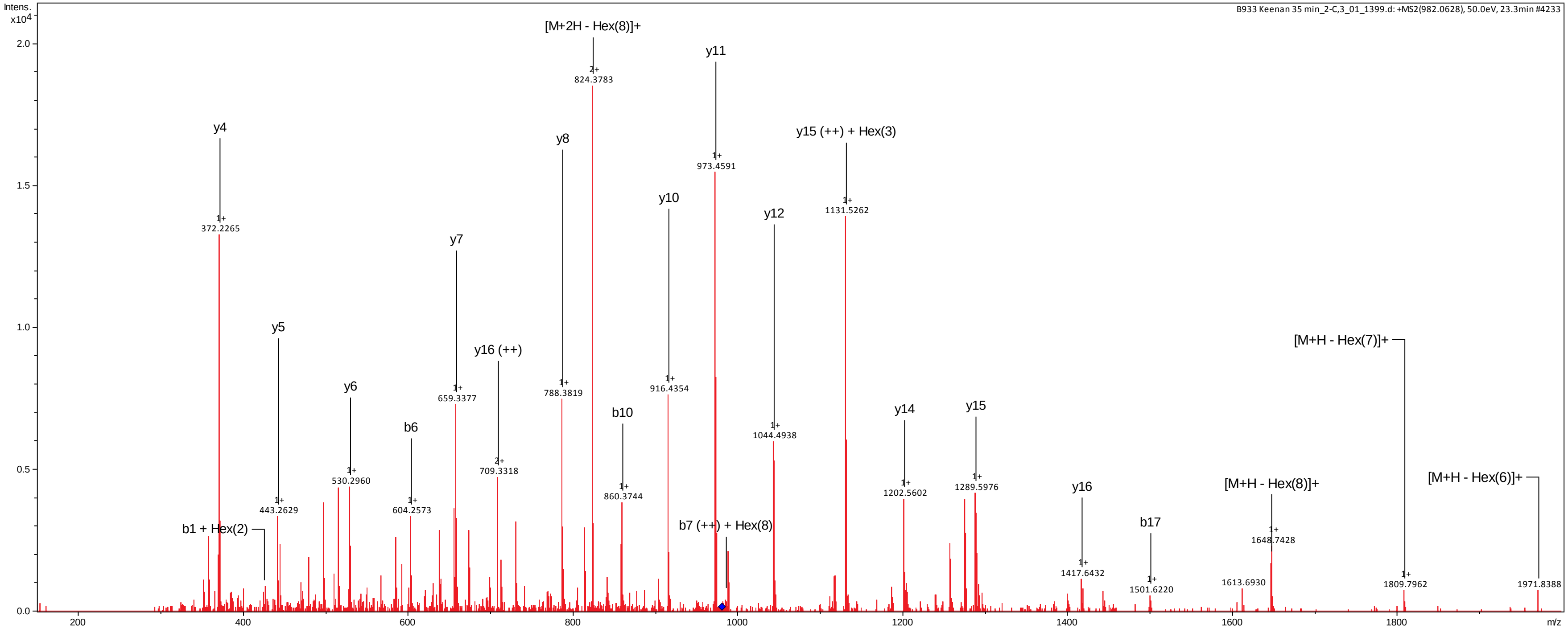

Time point	35 hr
SCO number	SCO4739
Precursor ion mass	1036.084
Charge	3
Retention time	23.1
Hex on peptide	9
e-value	0.0087
Site allocated?	N

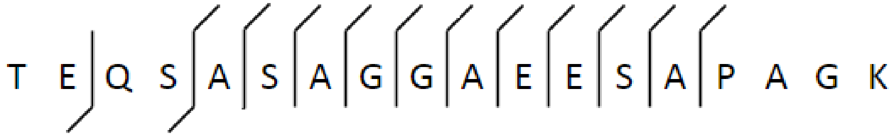

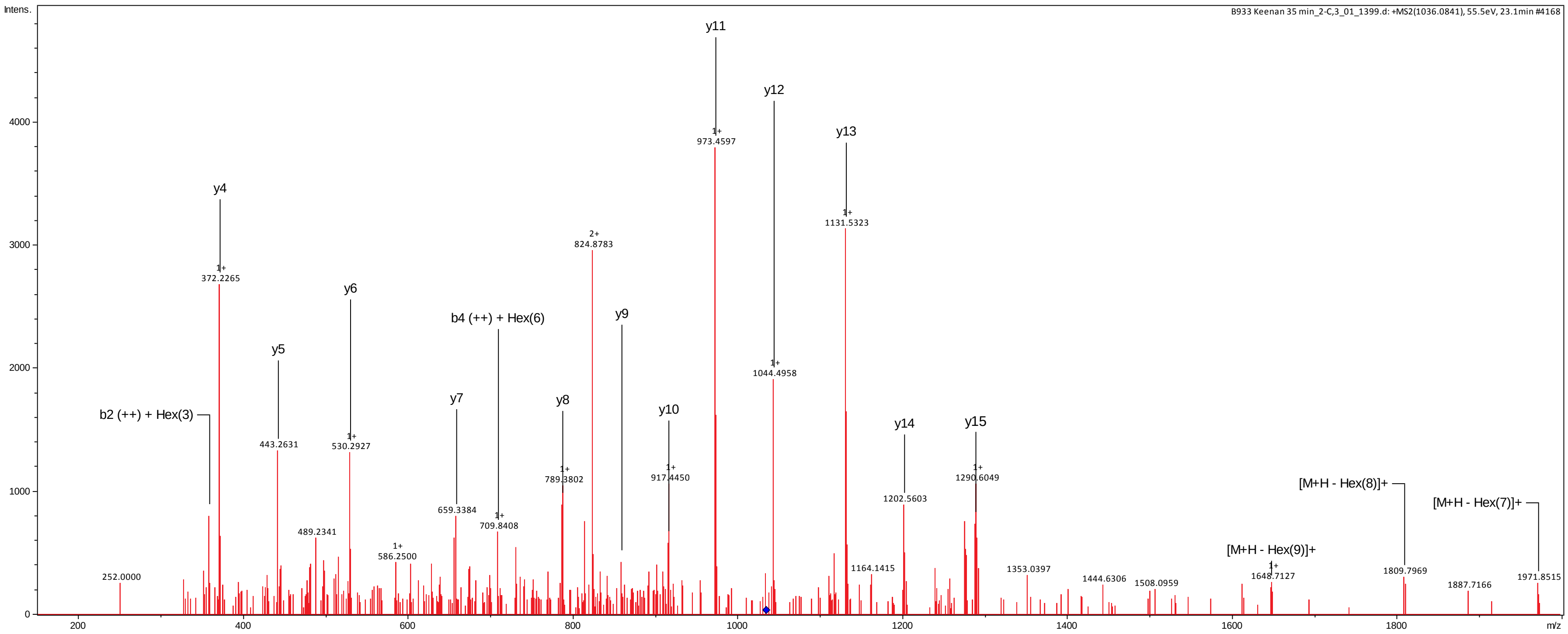

Time point	35 hr
SCO number	SCO1714
Precursor ion mass	723.318
Charge	2
Retention time	25.9
Hex on peptide	3
e-value	0.019
Site allocated?	N

T V T E P A A D R

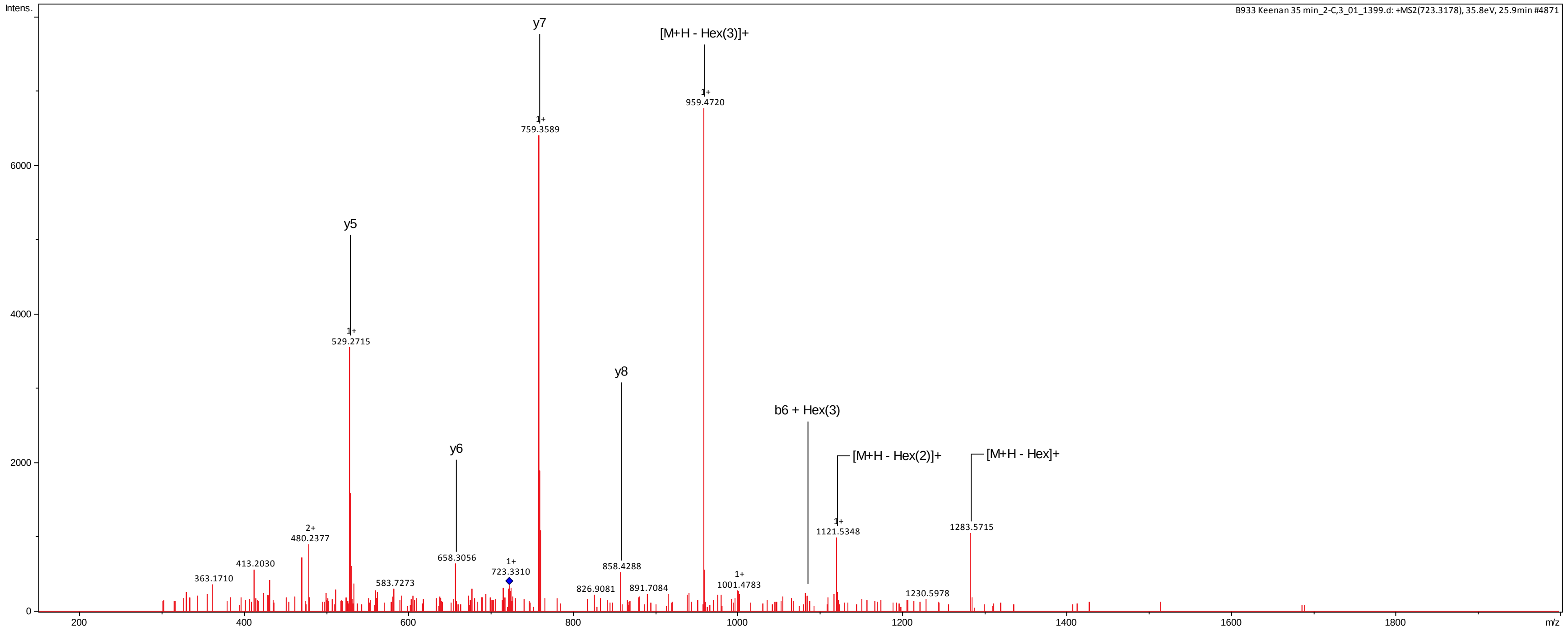

Time point	35 hr
SCO number	SCO3540
Precursor ion mass	910.927
Charge	2
Retention time	53.2
Hex on peptide	2
e-value	0.000055
Site allocated?	N

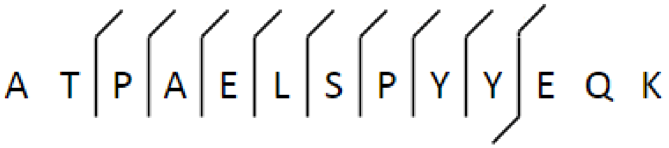

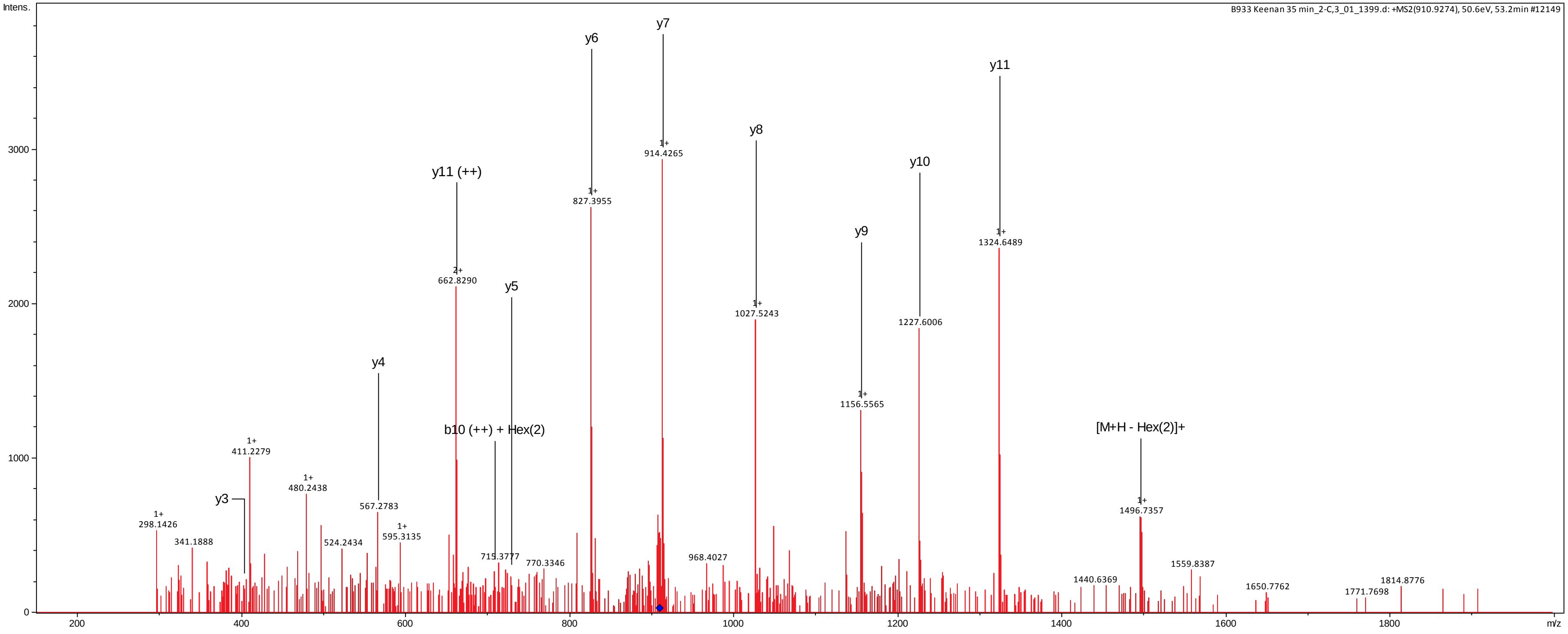

Time point	35 hr
SCO number	SCO0472
Precursor ion mass	1507.674
Charge	3
Retention time	136.5
Hex on peptide	9
e-value	0.00021
Site allocated?	N

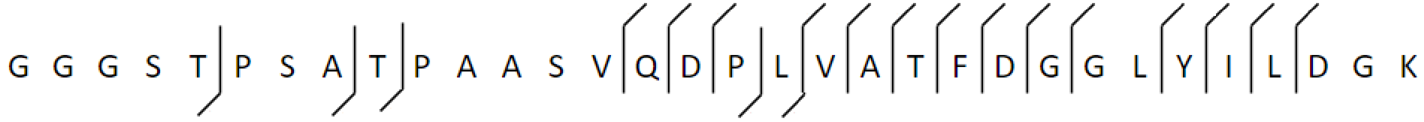

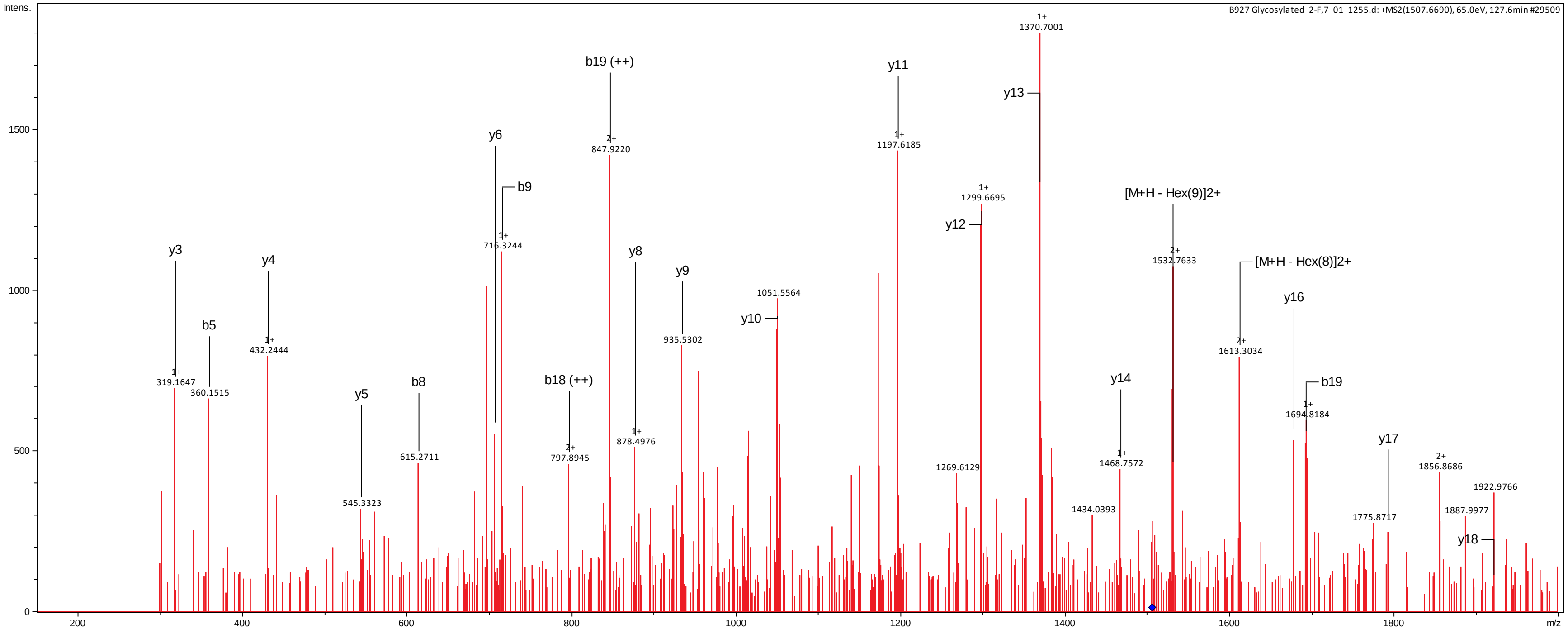

Time point	43 hr
SCO number	SCO5204
Precursor ion mass	1070.999
Charge	2
Retention time	73.2
Hex on peptide	1
e-value	0.012
Site allocated?	N

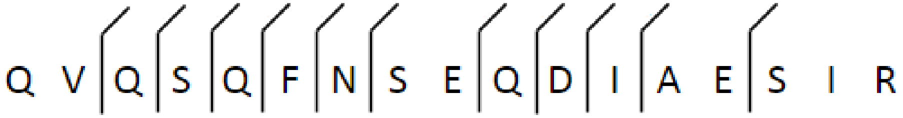

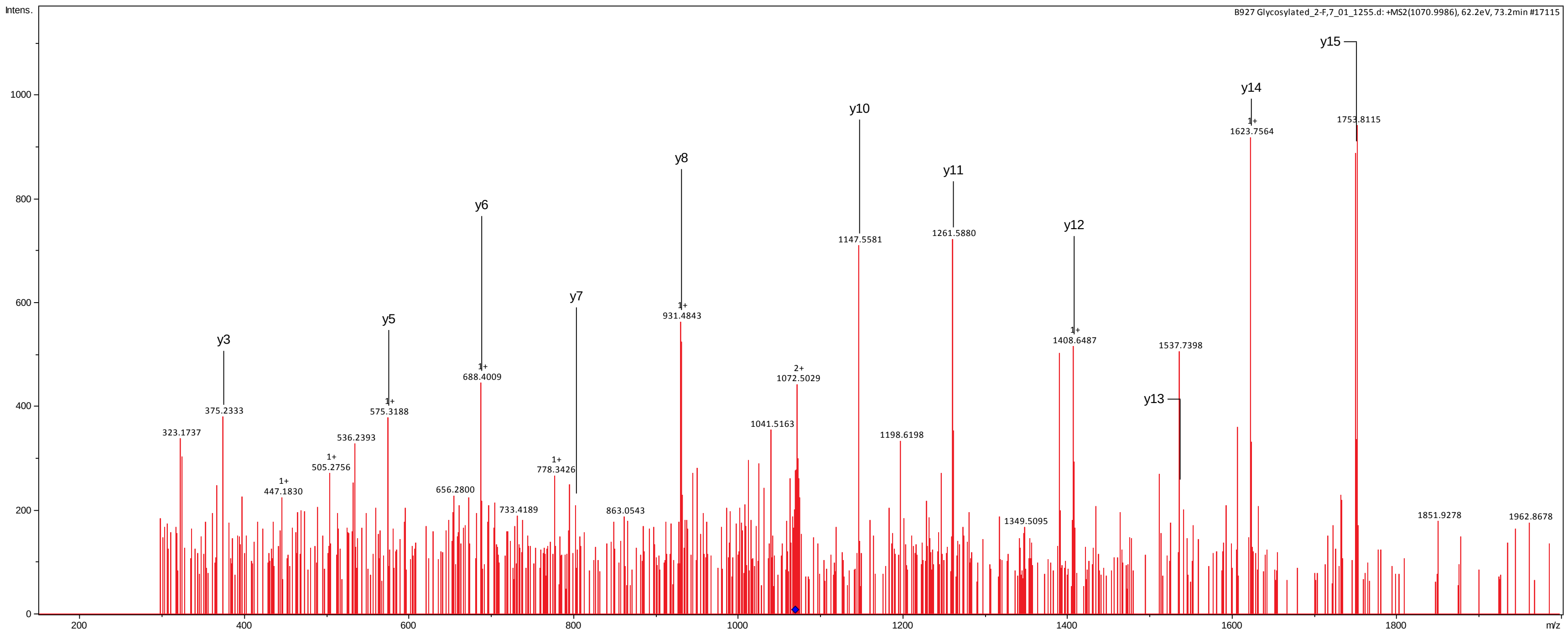

Time point	43 hr
SCO number	SCO4934
Precursor ion mass	853.373
Charge	2
Elution time	30.1
Hex on peptide	3
e-value	0.0036
Site allocated?	N

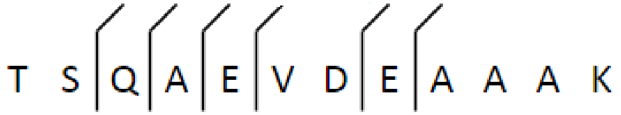

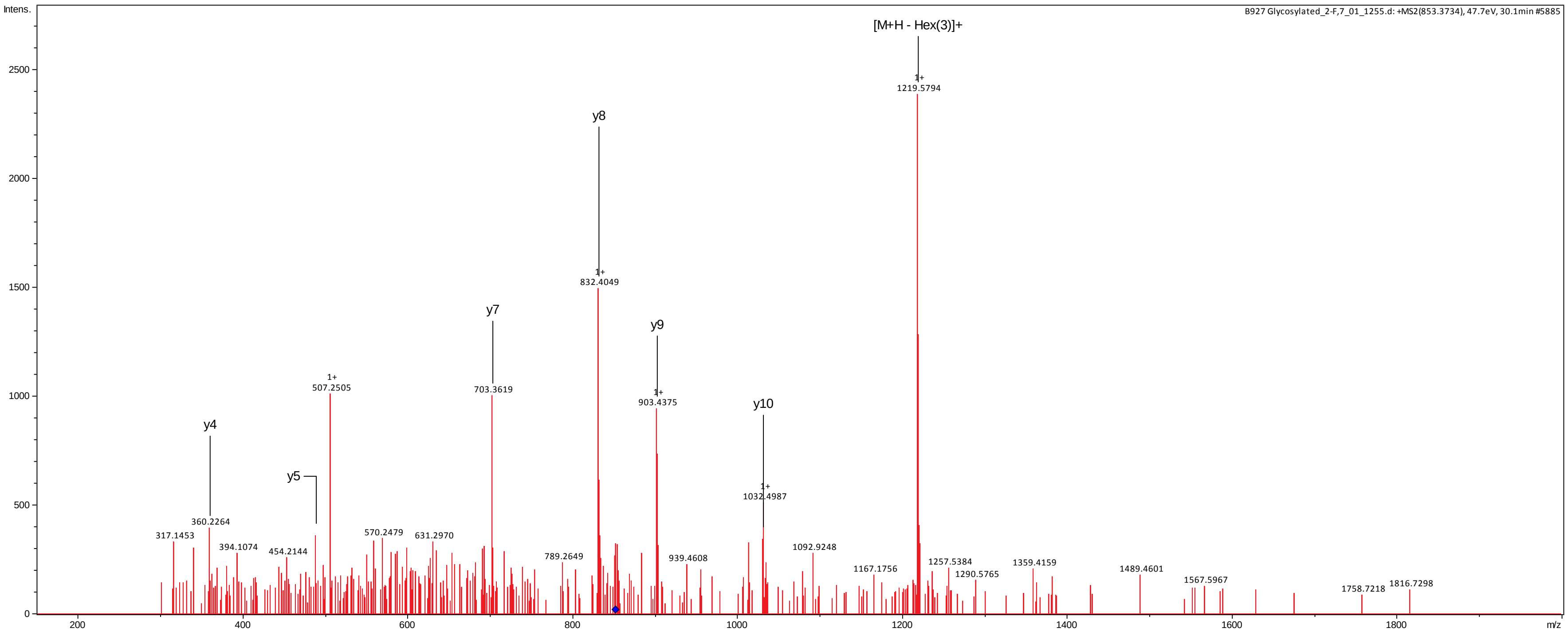

Time point	43 hr
SCO number	SCO3540
Precursor ion mass	910.921
Charge	2
Retention time	56.4
Hex on peptide	2
e-value	0.019
Site allocated?	T2

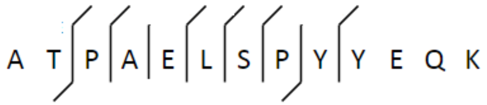

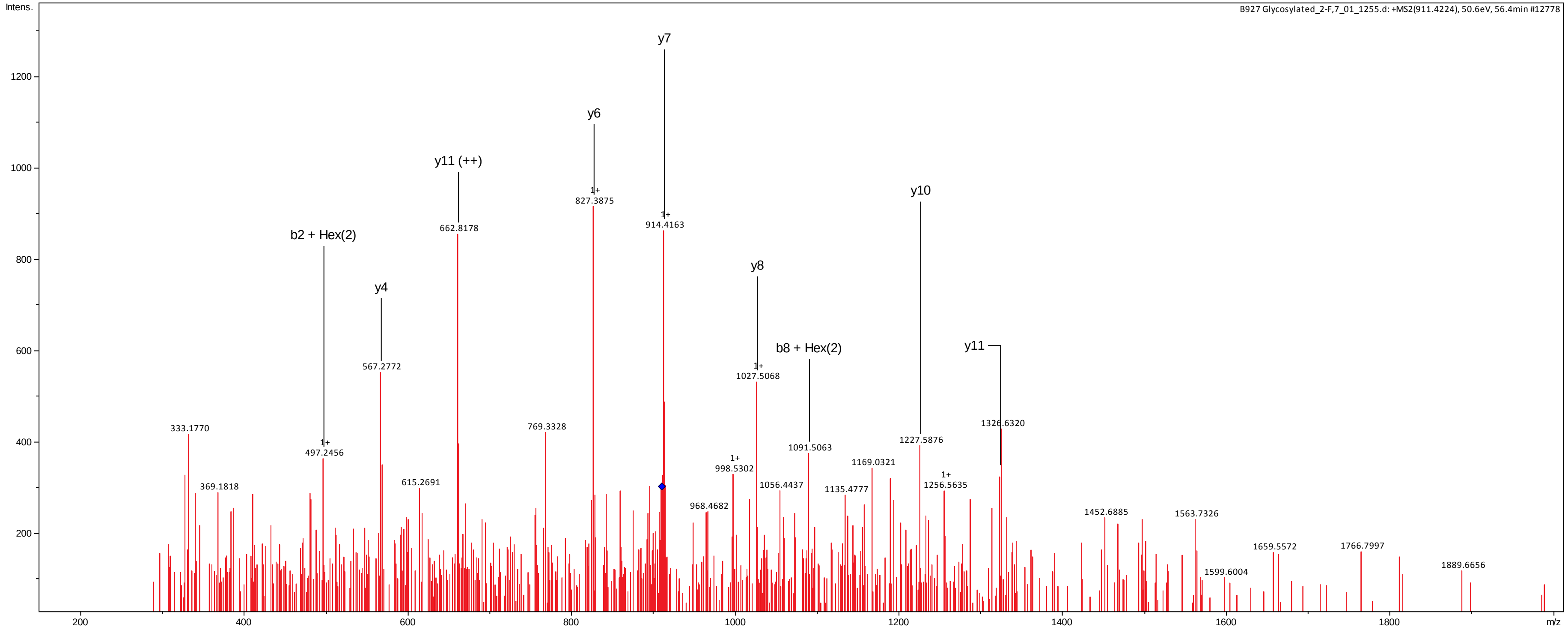

Time point	43 hr
SCO number	SCO3357
Precursor ion mass	992.761
Charge	3
Retention time	35.9
Hex on peptide	6
e-value	0.013
Site allocated?	N

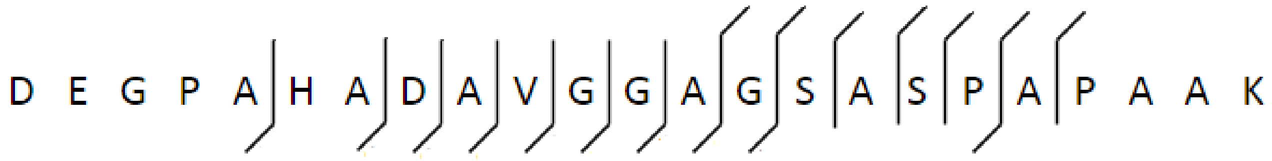

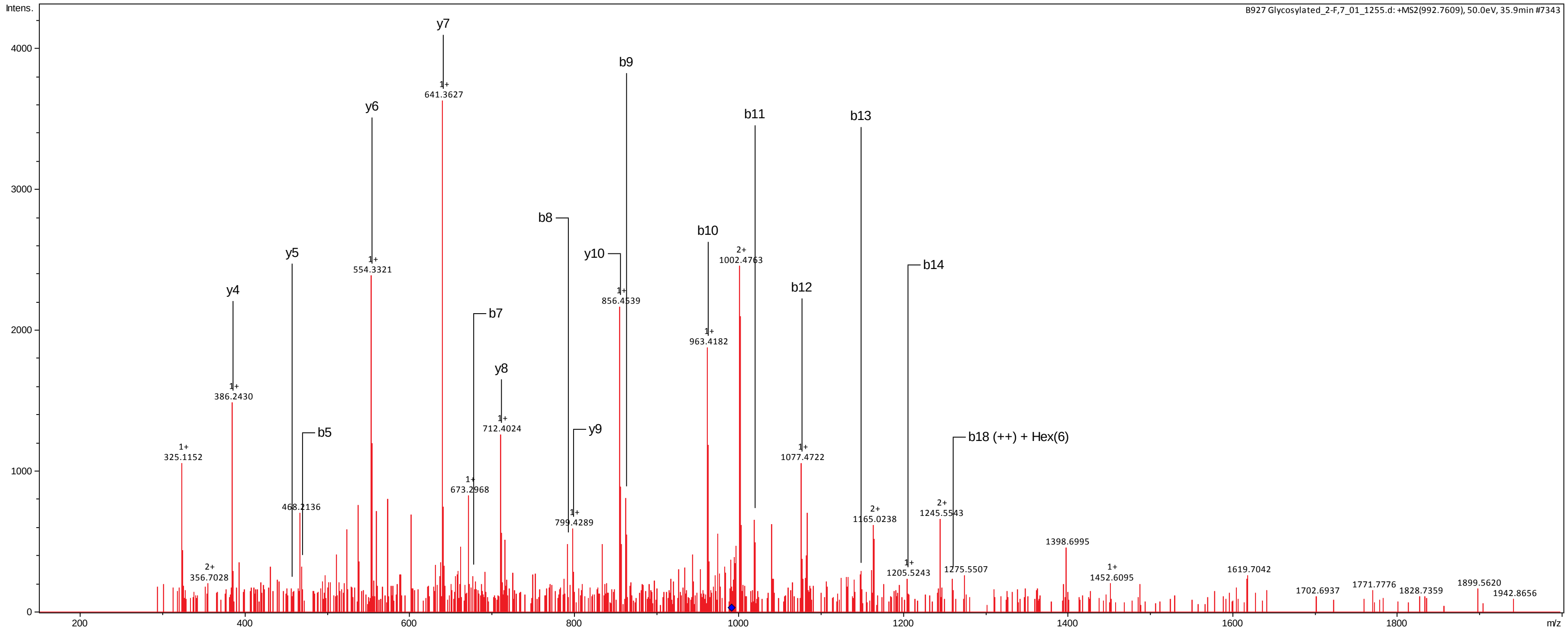

Time point	60 hr
SCO number	SCO4934
Precursor ion mass	772.350
Charge	2
Retention time	27.7
Hex on peptide	2
e-value	0.00019
Site allocated?	N

T S Q A E V D E A A A K

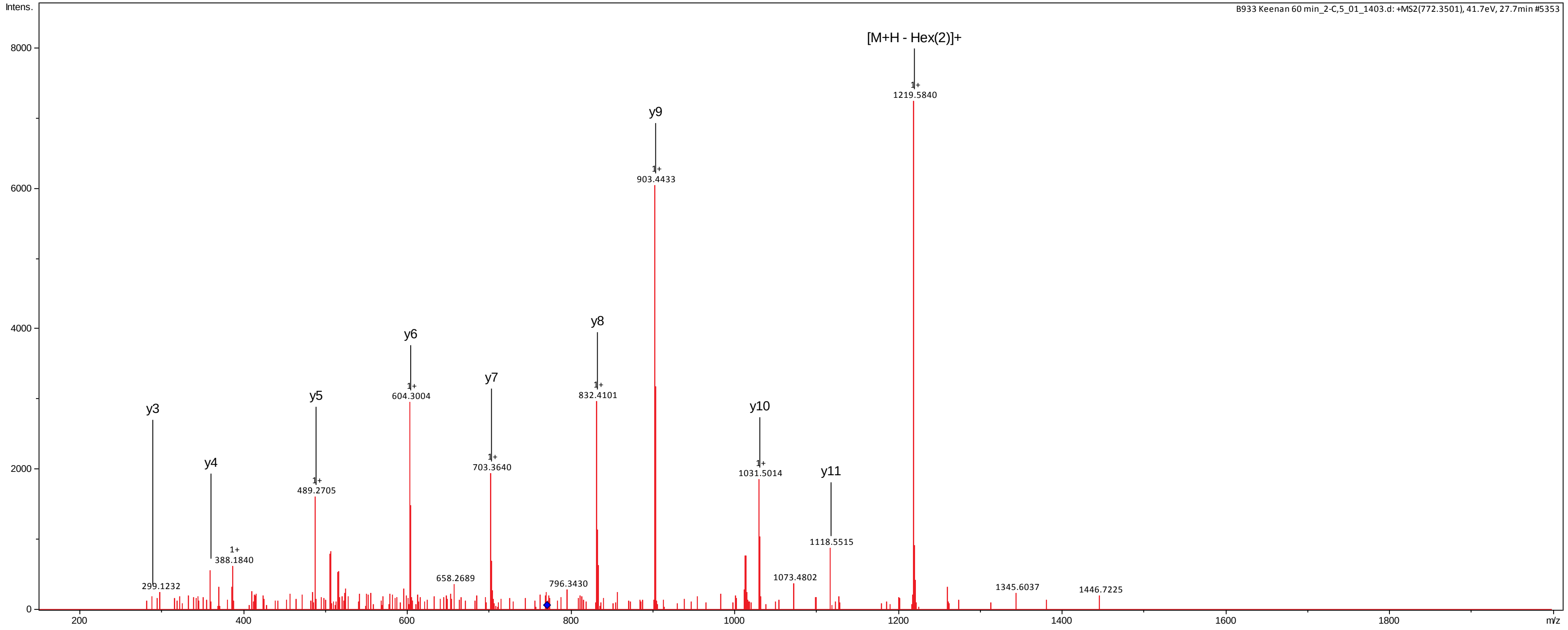

Time point	60 hr
SCO number	SCO4934
Precursor ion mass	853.375
Charge	2
Retention time	27.4
Hex on peptide	3
e-value	0.024
Site allocated?	N

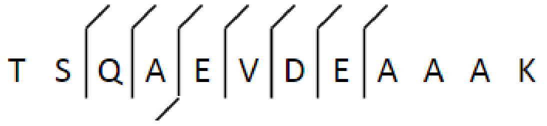

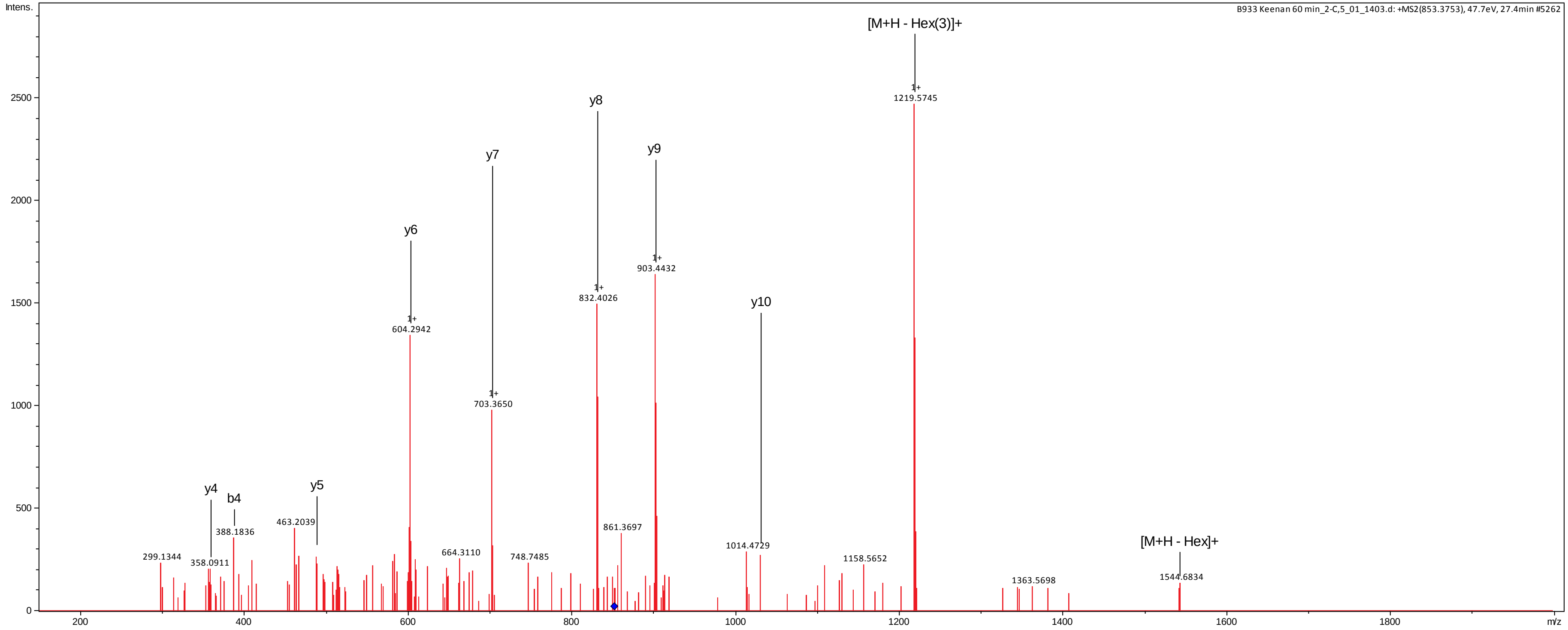

Time point	60 hr
SCO number	SCO4141
Precursor ion mass	562.279
Charge	3
Retention time	31.2
Hex on peptide	1
e-value	0.04
Site allocated?	N

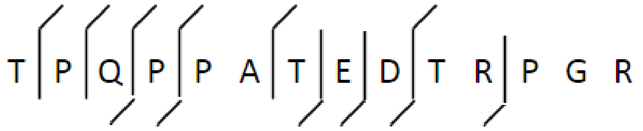

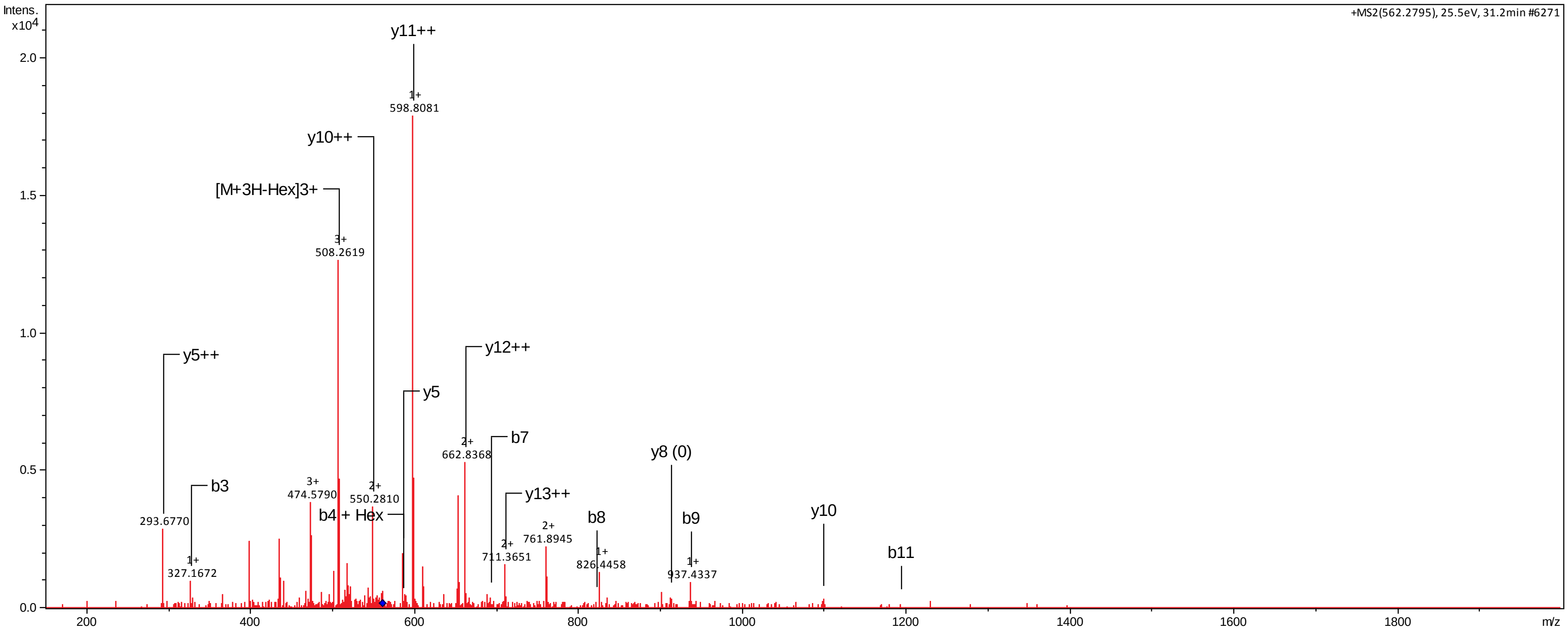

Time point	60 hr
SCO number	SCO4905
Precursor ion mass	973.935
Charge	2
Retention time	46.7
Hex on peptide	3
e-value	0.0091
Site allocated?	N

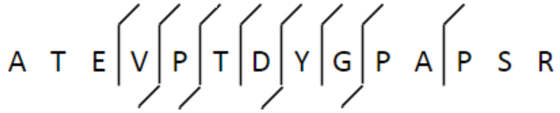

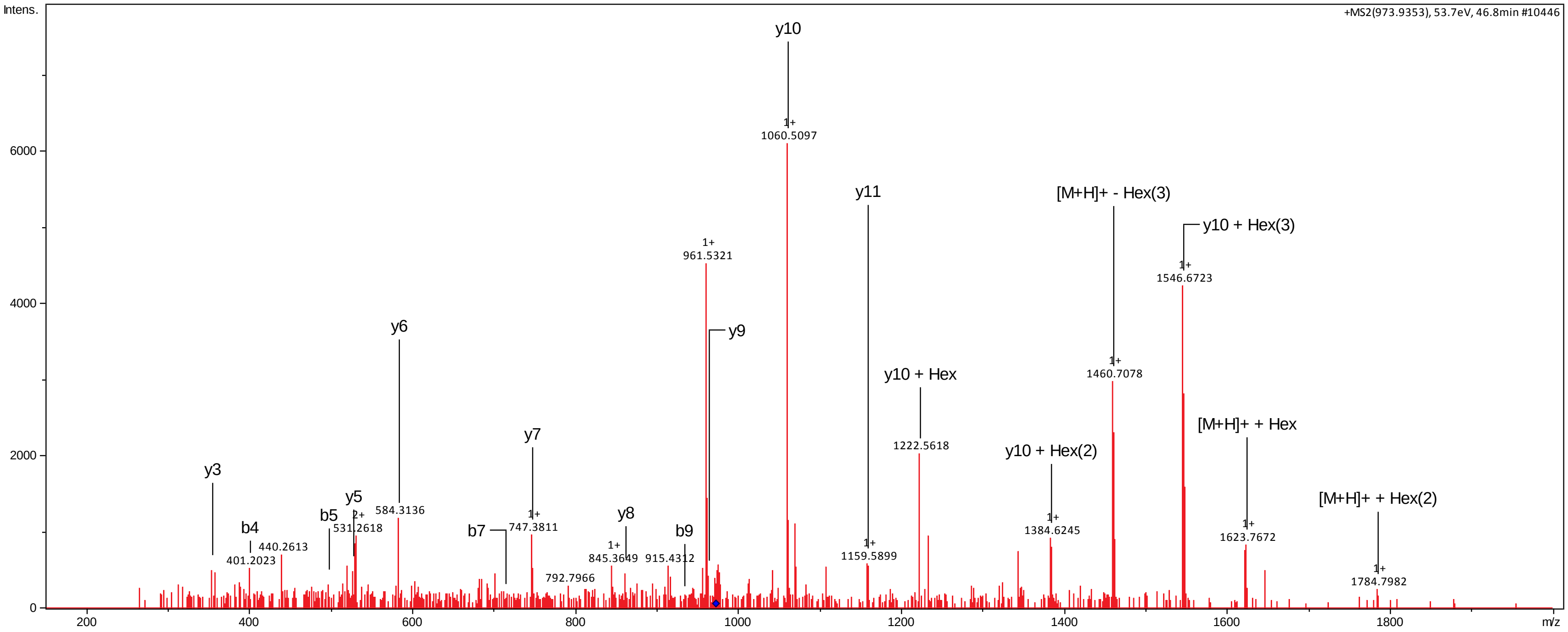

Time point	60 hr
SCO number	SCO4739
Precursor ion mass	982.063
Charge	3
Retention time	23.8
Hex on peptide	8
e-value	0.0041
Site allocated?	N

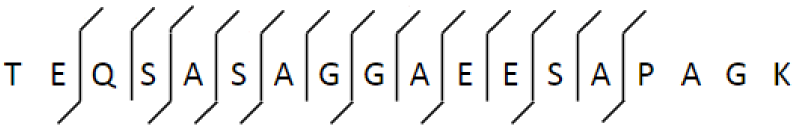

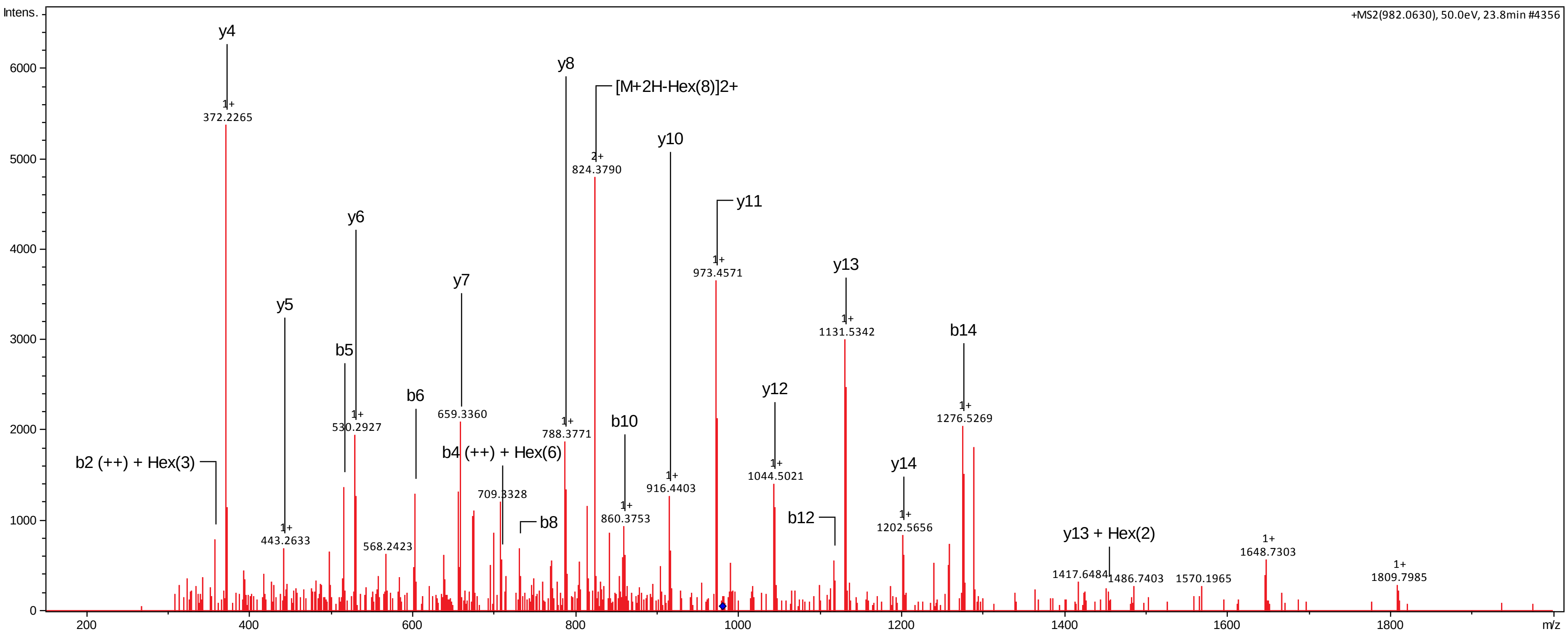

Time point	20 hr
SCO number	SCO4739
Precursor ion mass	1067.459
Charge	2
Retention time	25.7
Hex on peptide	3
e-value	1.10E-08
Site allocated?	N

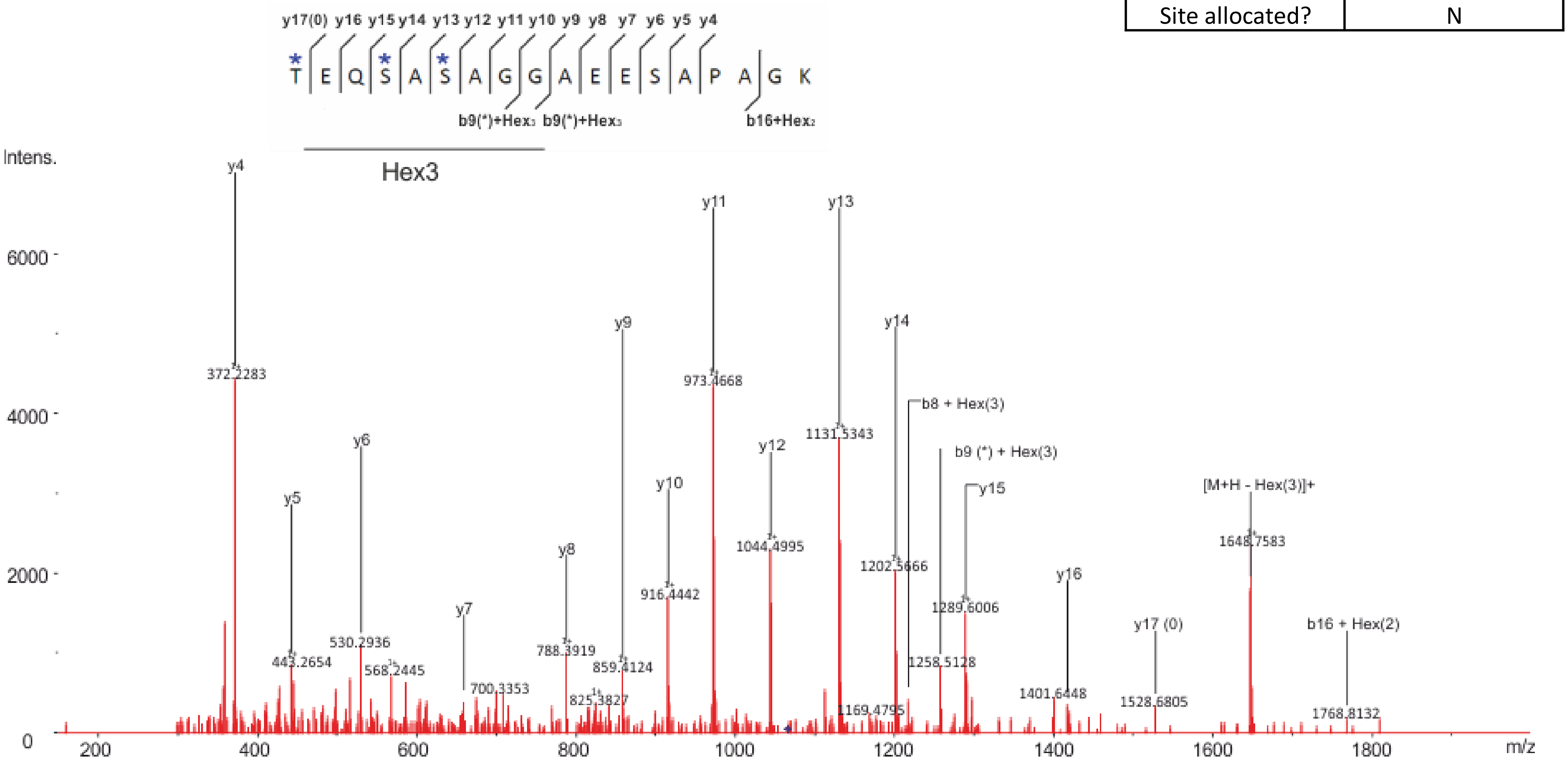

Time point	20 hr
SCO number	SCO4905
Precursor ion mass	865.783
Charge	3
Retention time	122.5
Hex on peptide	1
e-value	1.90E-04
Site allocated?	N

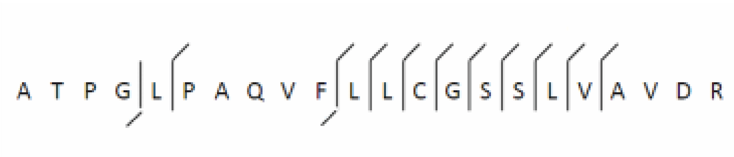

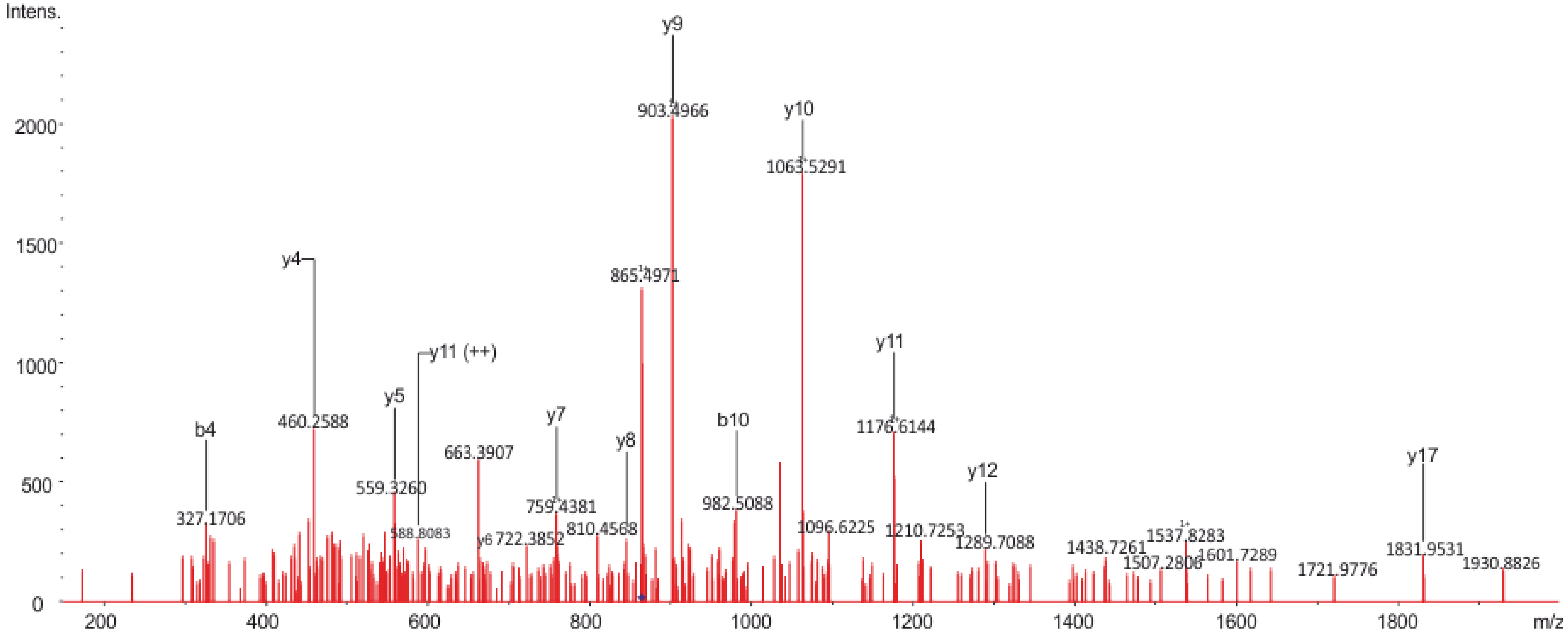

Spectra generated using HCD and ETD fragmentation techniques on the Thermo Orbitrap Fusion Tribrid mass spectrometer.

Key:

Ion Type	Description
c(++)	doubly charged series
y(++)	doubly charged series
z(+2)	z + 2
z(++)	doubly charged series
z(+2++)	z+2 doubly charged series

SCO Number	SCO4142
Precursor m/z	750.712
Charge	3
Retention time	91.5
Scan number	24203
Hex on peptide	1
e-value	0.0028
Site allocated?	Ser251
Method	ETD_IT

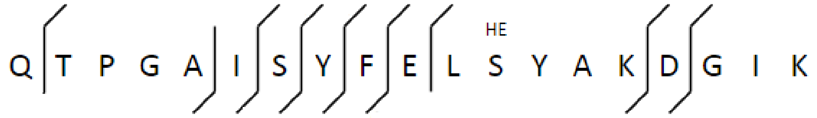

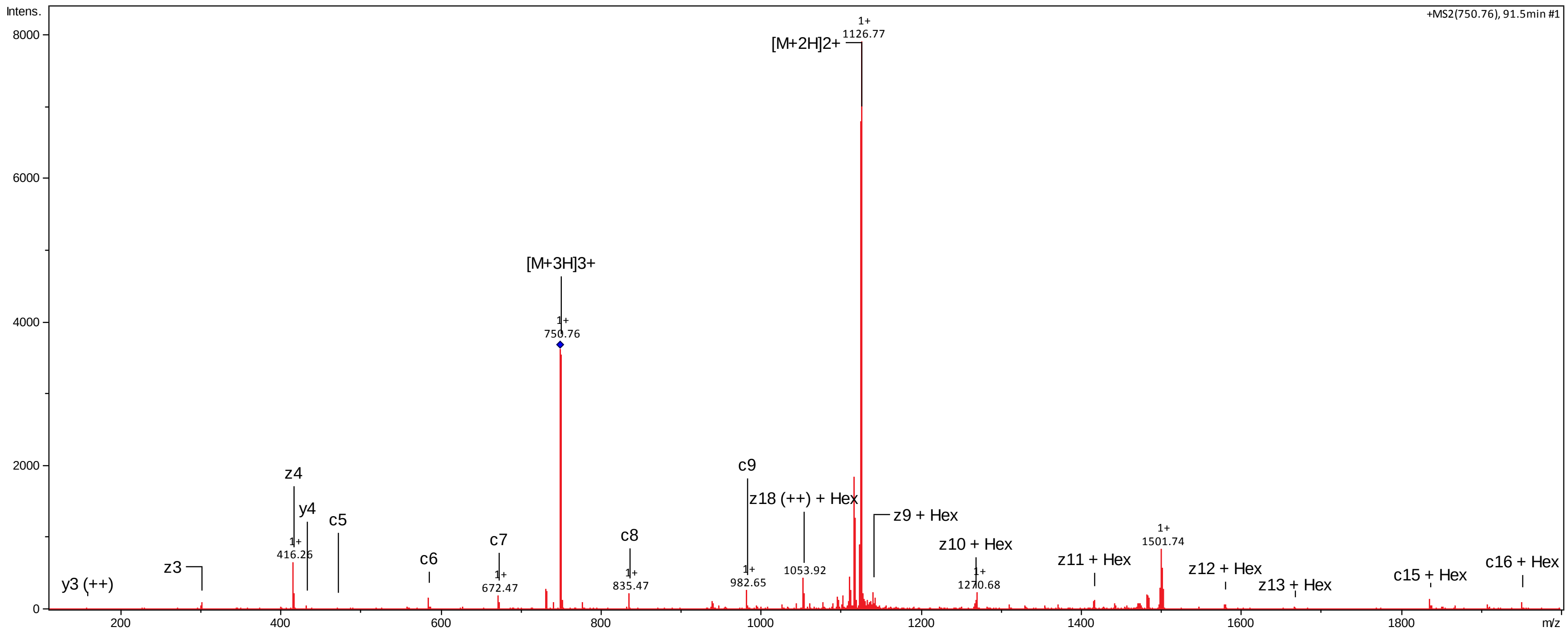

SCO Number	SCO3046
Precursor m/z	909.820
Charge	3
Retention time	89.4
Scan number	22874
Hex on peptide	2
e-value	0.0000031
Site allocated?	Thr47
Method	HCD_IT, ETD_OT

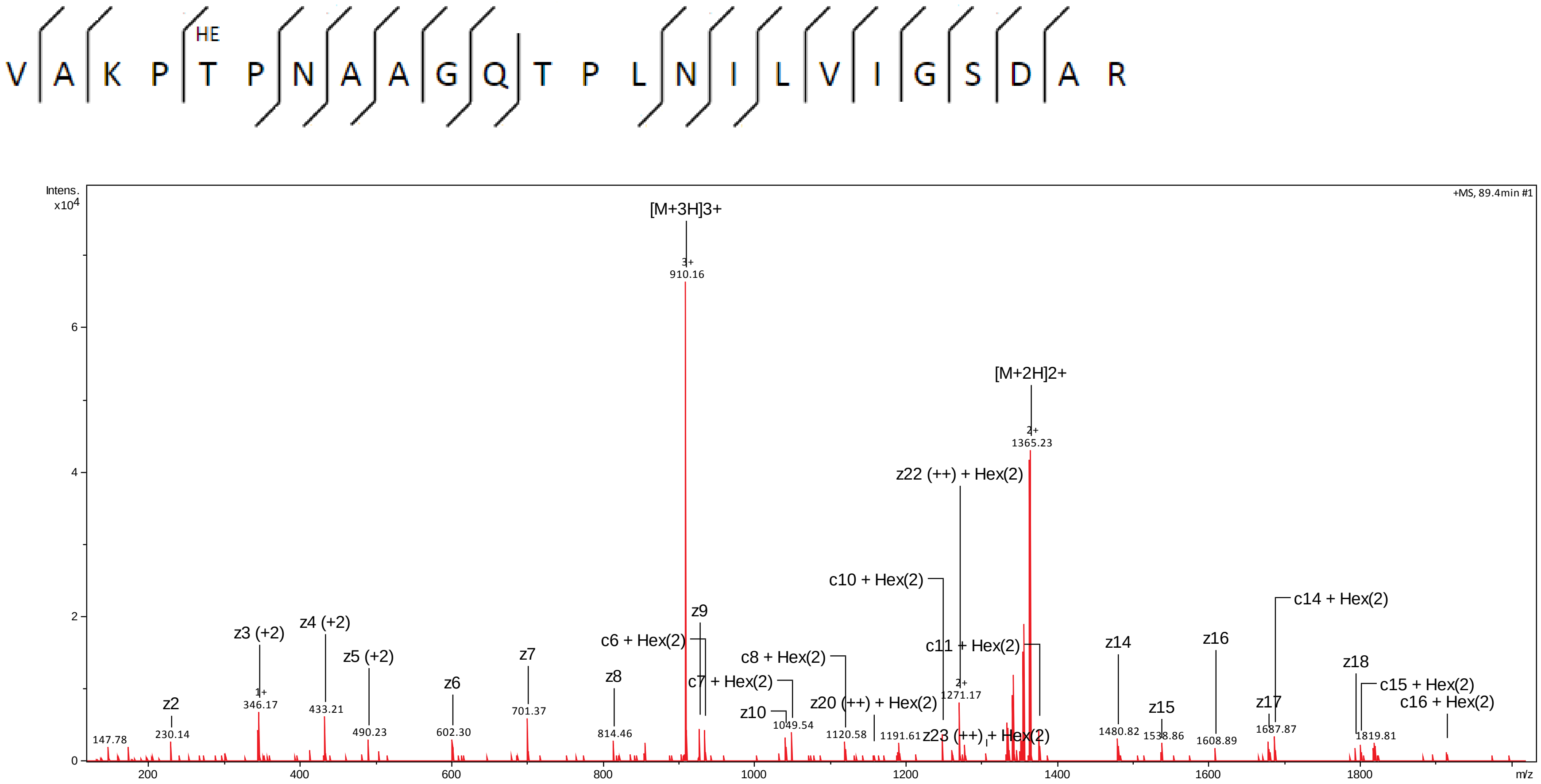

SCO Number	SCO4968
Precursor m/z	811.392
Charge	3
Retention time	31.4
Scan number	6232
Hex on peptide	1
e-value	0.0000091
Site allocated?	Ser65
Method	ETD_OT

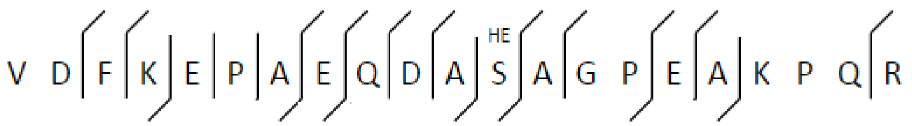

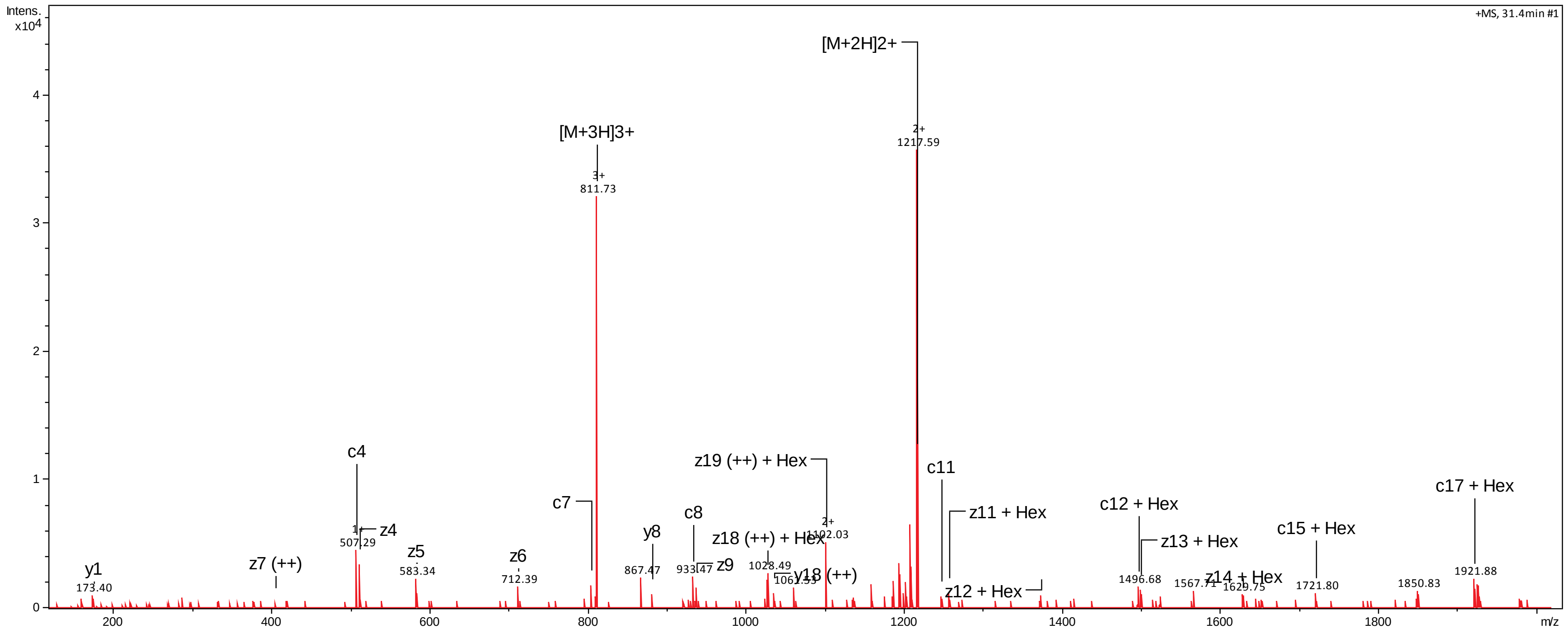

SCO Number	SCO3353
Precursor m/z	626.960
Charge	3
Retention time	17.5
Scan number	2027
Hex on peptide	2
e-value	0.000016
Site allocated?	Thr94
Method	HCD_IT, ETD_IT, ETD_OT

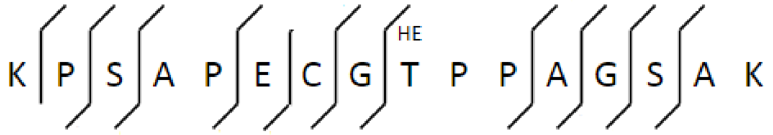

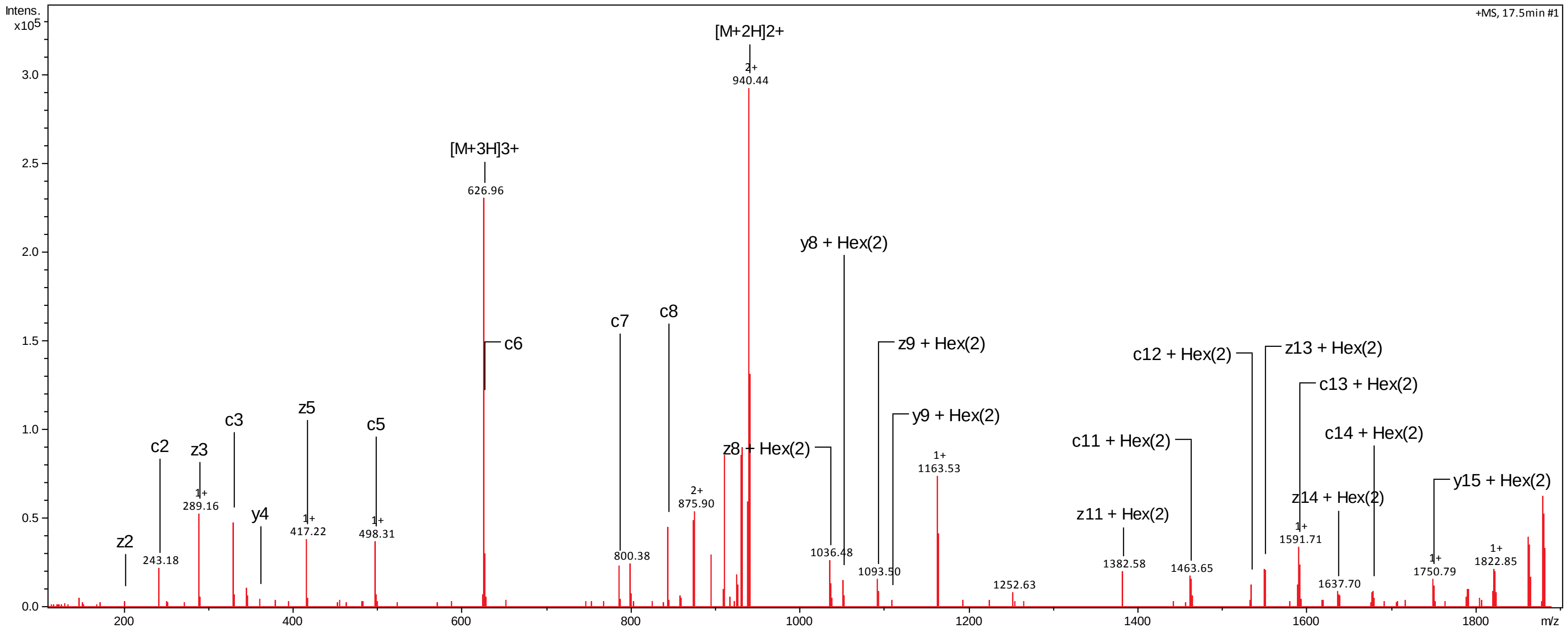

SCO Number	SCO3353
Precursor m/z	680.978
Charge	3
Retention time	17.3
Scan number	4050
Hex on peptide	3
e-value	0.00071
Site allocated?	Thr94
Method	HCD_IT, ETD_IT, ETD_OT

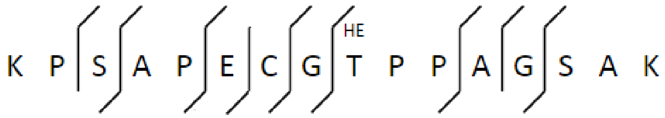

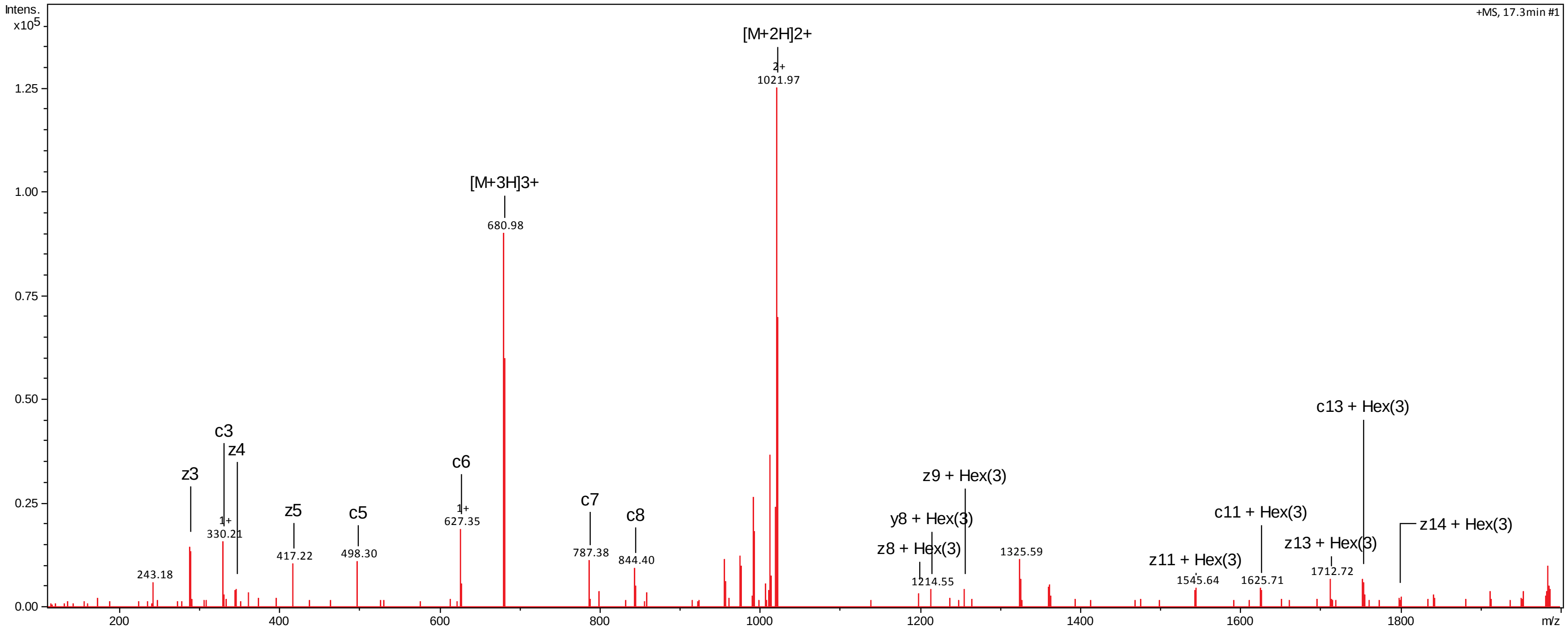

SCO Number	SCO4141
Precursor m/z	562.277
Charge	3
Retention time	18.7
Scan number	2571
Hex on peptide	1
e-value	0.00021
Site allocated?	Thr15
Method	HCD_IT, ETD_IT, ETD_OT

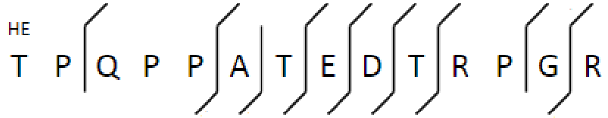

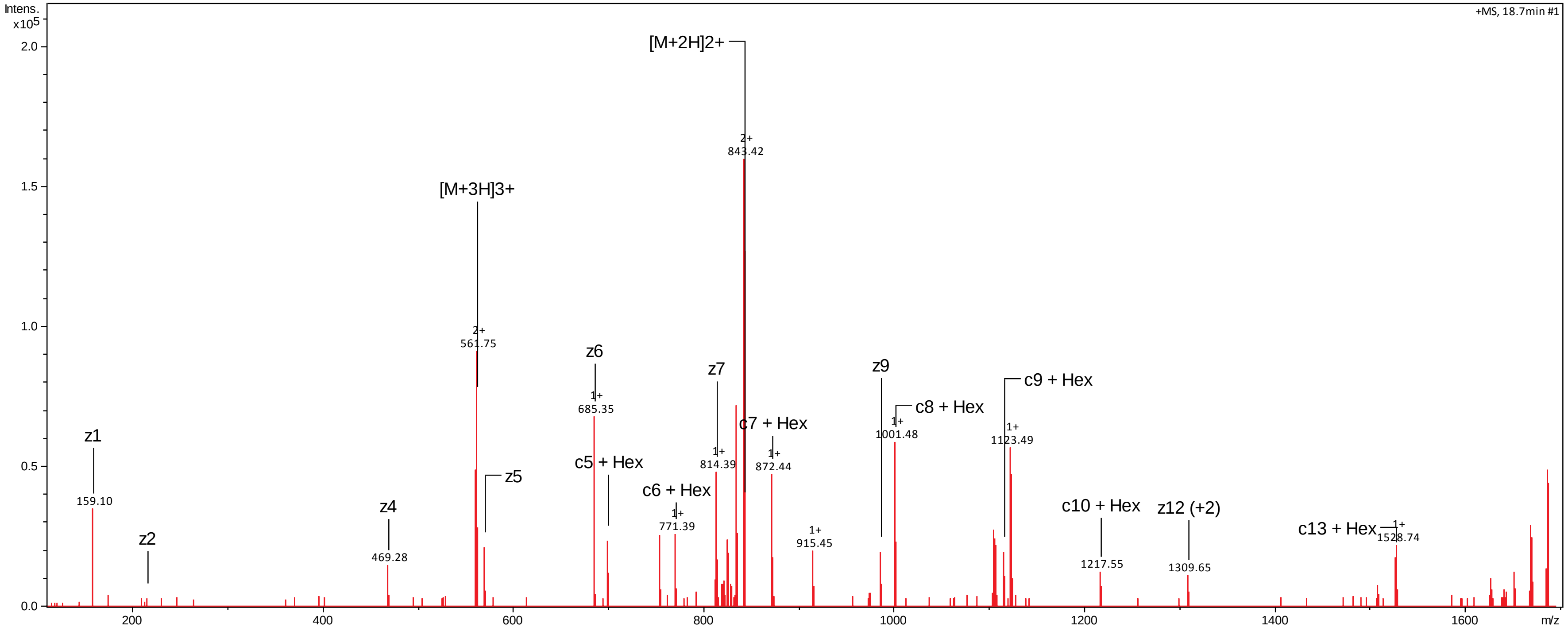

SCO Number	SCO5751
Precursor m/z	695.938
Charge	5
Retention time	26.8
Scan number	4858
Hex on peptide	6
e-value	0.00044
Site allocated?	Ser193, Ser195
Method	ETD_OT

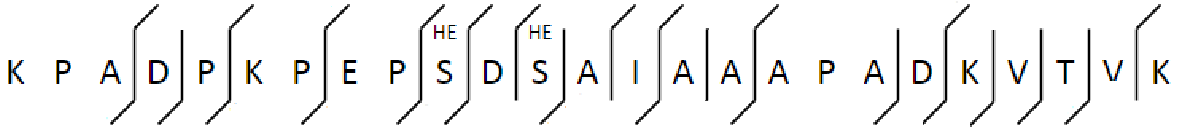

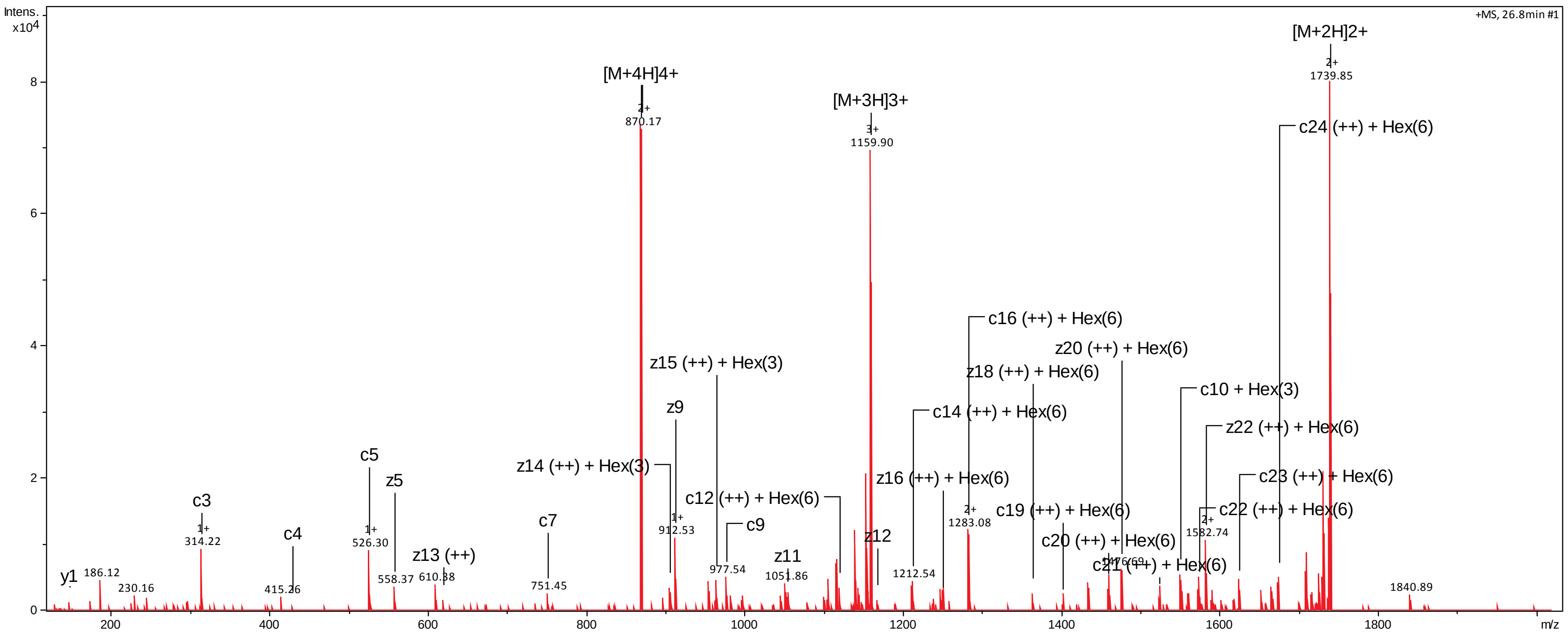

SCO Number	SCO3357
Precursor m/z	938.741
Charge	3
Retention time	20.4
Scan number	2972
Hex on peptide	6
e-value	0.00076
Site allocated?	Ser37, Ser39
Method	ETD_OT

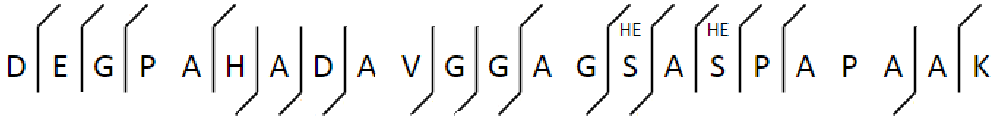

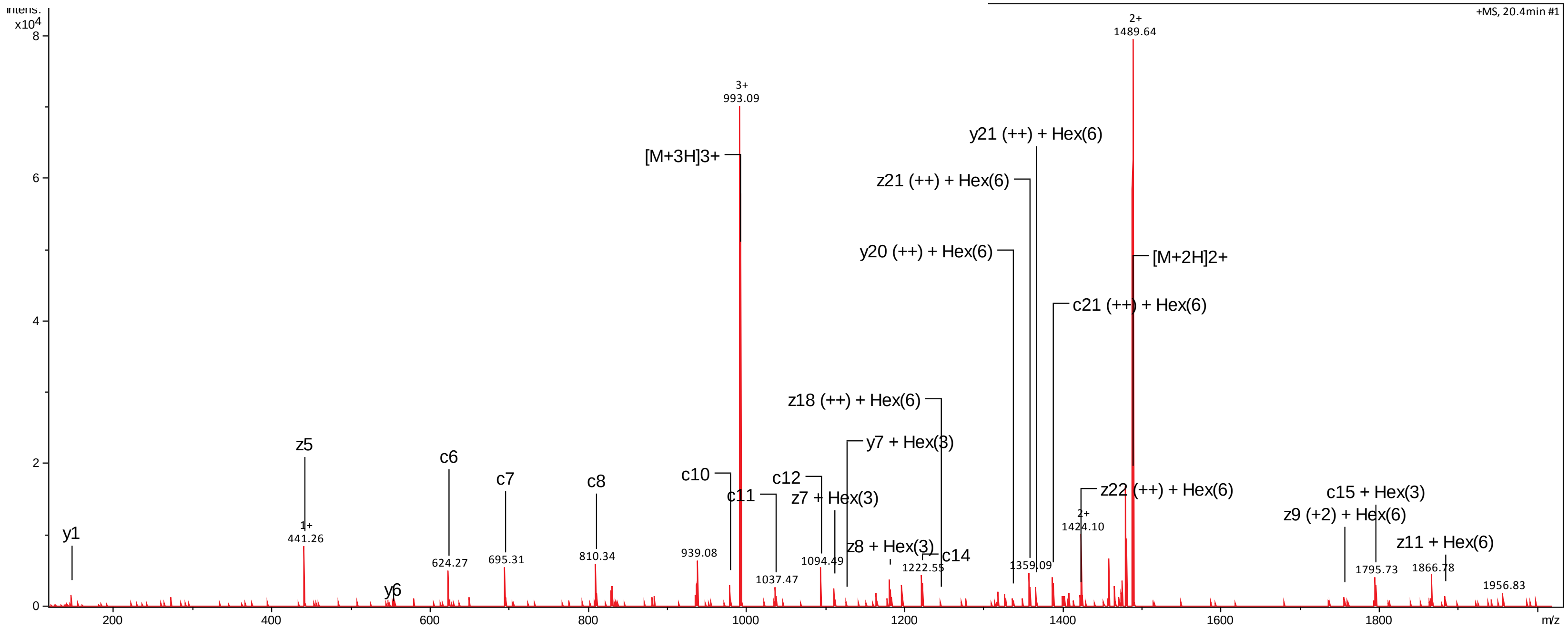

SCO Number	SCO5776
Precursor m/z	609.072
Charge	4
Retention time	94.1
Scan number	60208
Hex on peptide	1
e-value	0.0013
Site allocated?	Ser114
Method	HCD_IT

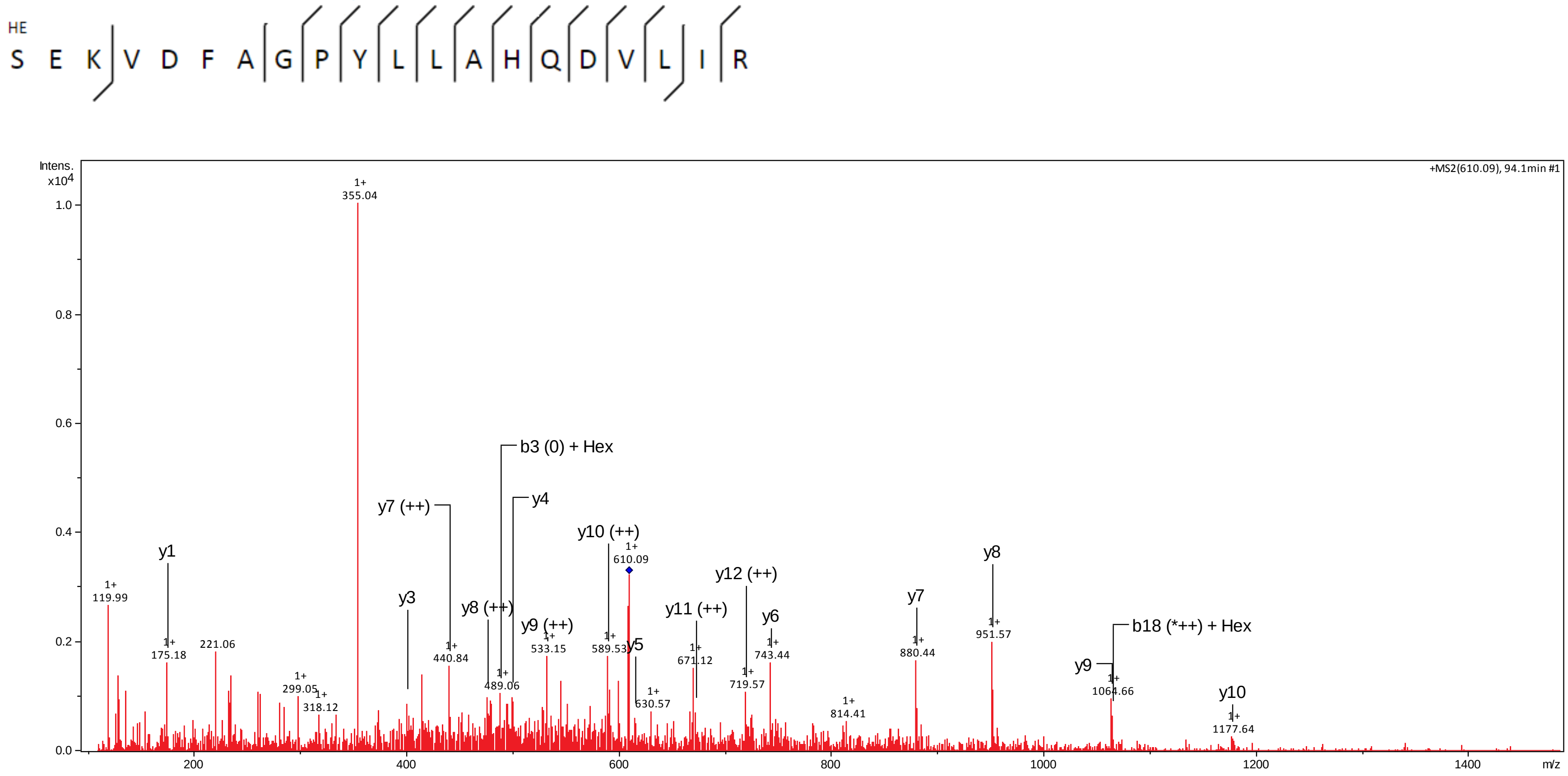

SCO Number	SCO2838
Precursor m/z	683.341
Charge	2
Retention time	18.5
Scan number	2576
Hex on peptide	2
e-value	0.002
Site allocated?	Thr38
Method	ETD_IT

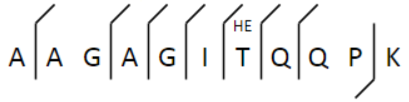

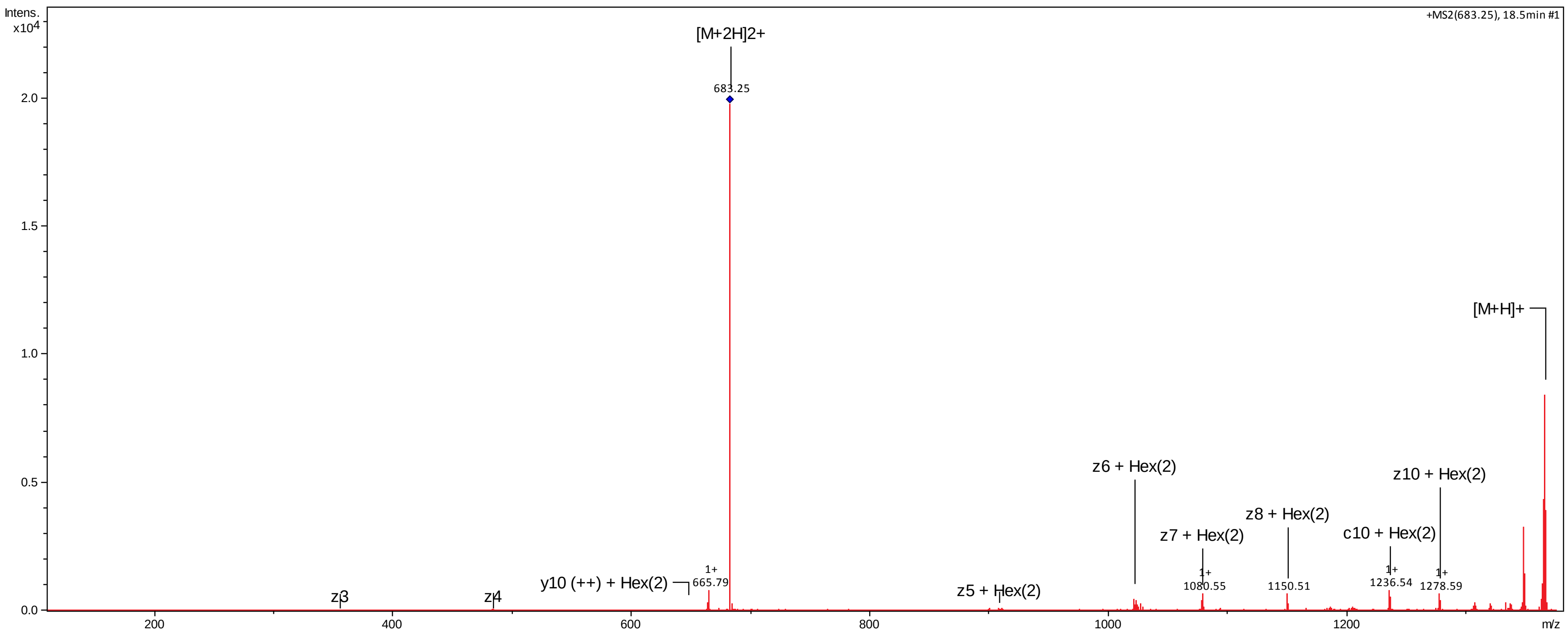

SCO Number	SCO4307
Precursor m/z	404.545
Charge	3
Retention time	32.5
Scan number	6751
Hex on peptide	1
e-value	0.0006
Site allocated?	Thr83
Method	ETD_IT

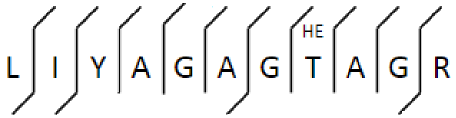

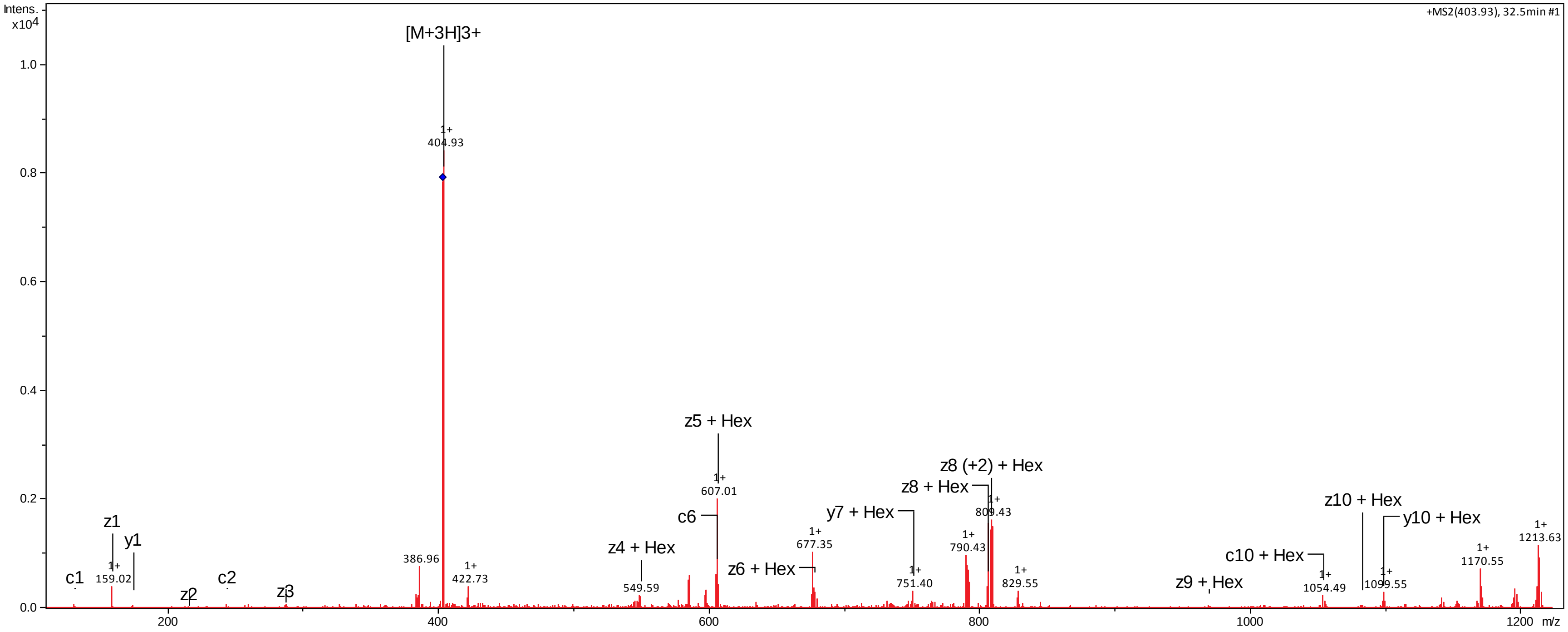

SCO Number	SCO4142
Precursor m/z	379.541
Charge	3
Retention time	23.9
Scan number	3598
Hex on peptide	1
e-value	0.0081
Site allocated?	Thr259
Method	ETD_OT

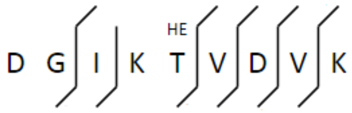

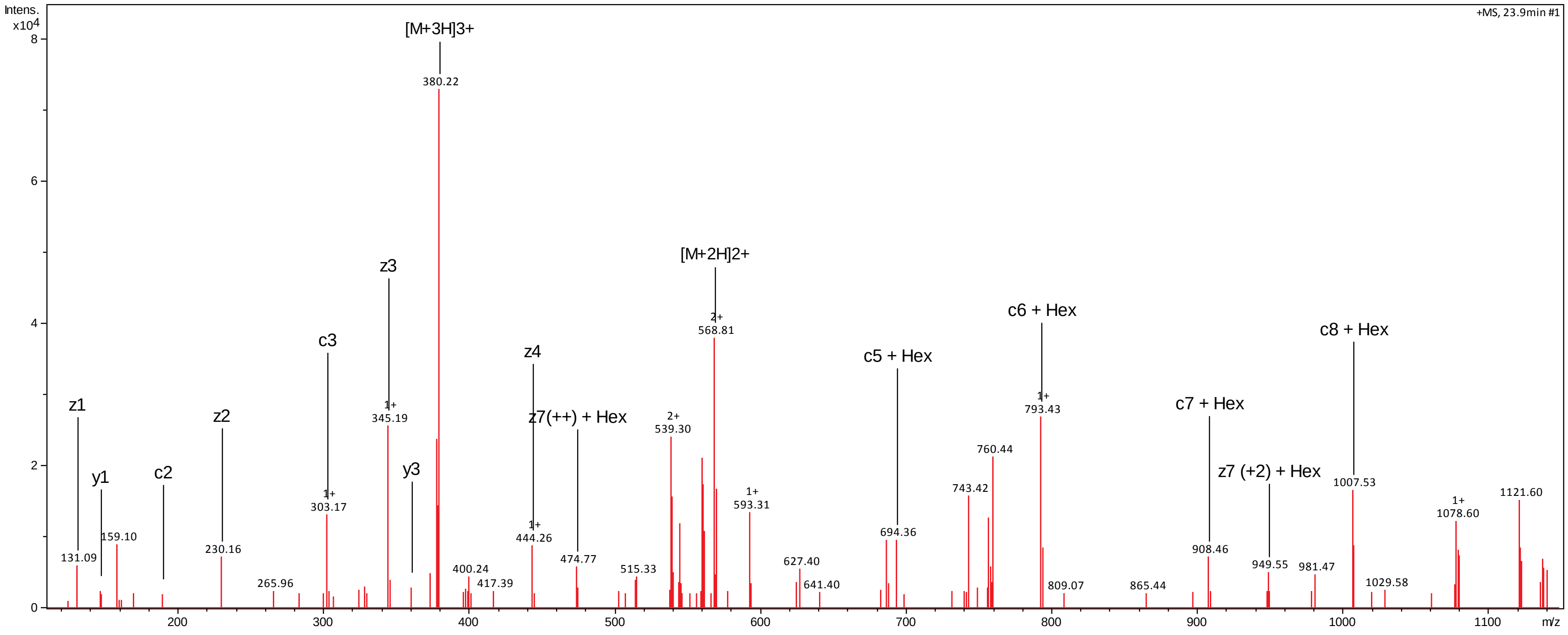

SCO Number	SCO4256
Precursor m/z	527.764
Charge	4
Retention time	15.5
Scan number	1712
Hex on peptide	3
e-value	0.018
Site allocated?	Ser317
Method	ETD_OT

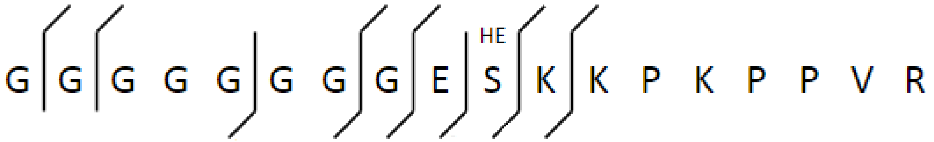

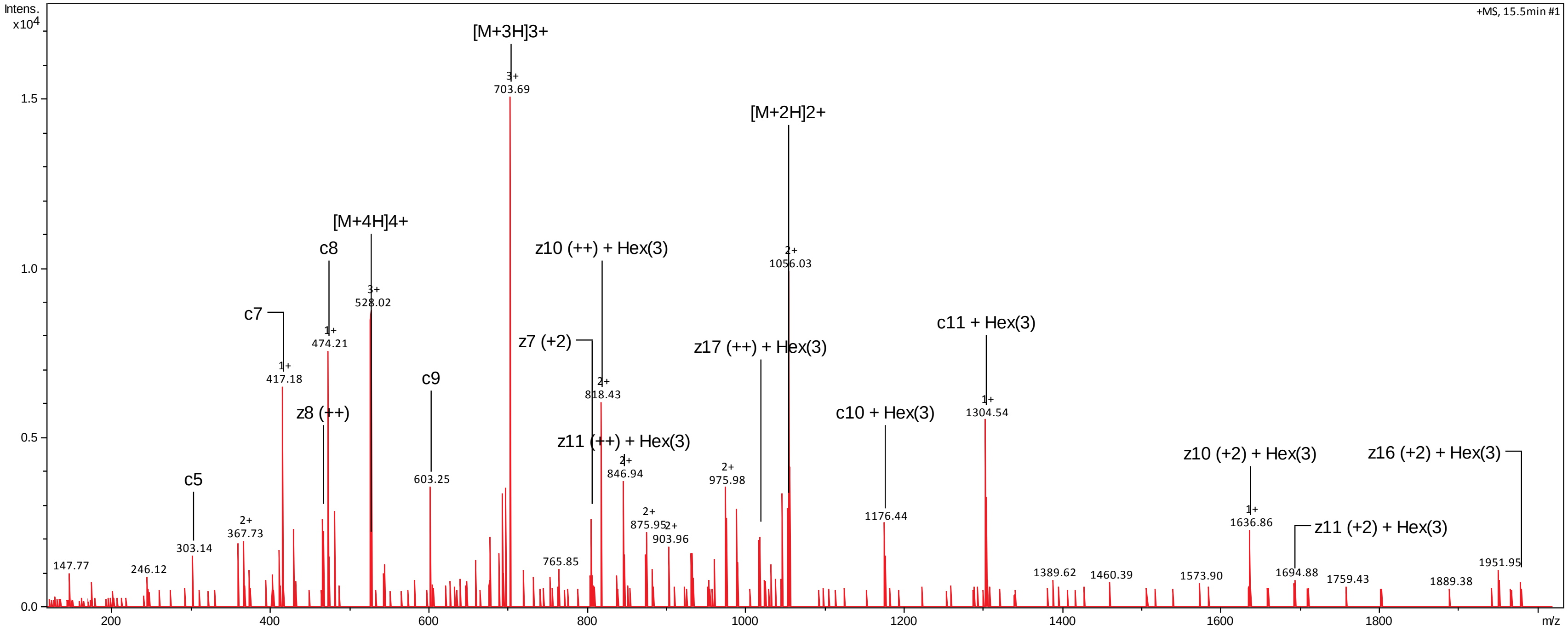

SCO Number	SCO7218
Precursor m/z	759.991
Charge	3
Retention time	22.9
Scan number	3332
Hex on peptide	3
e-value	0.00066
Site allocated?	N
Method	ETD_OT

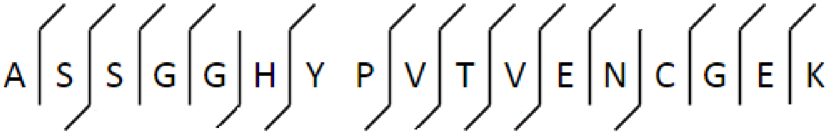

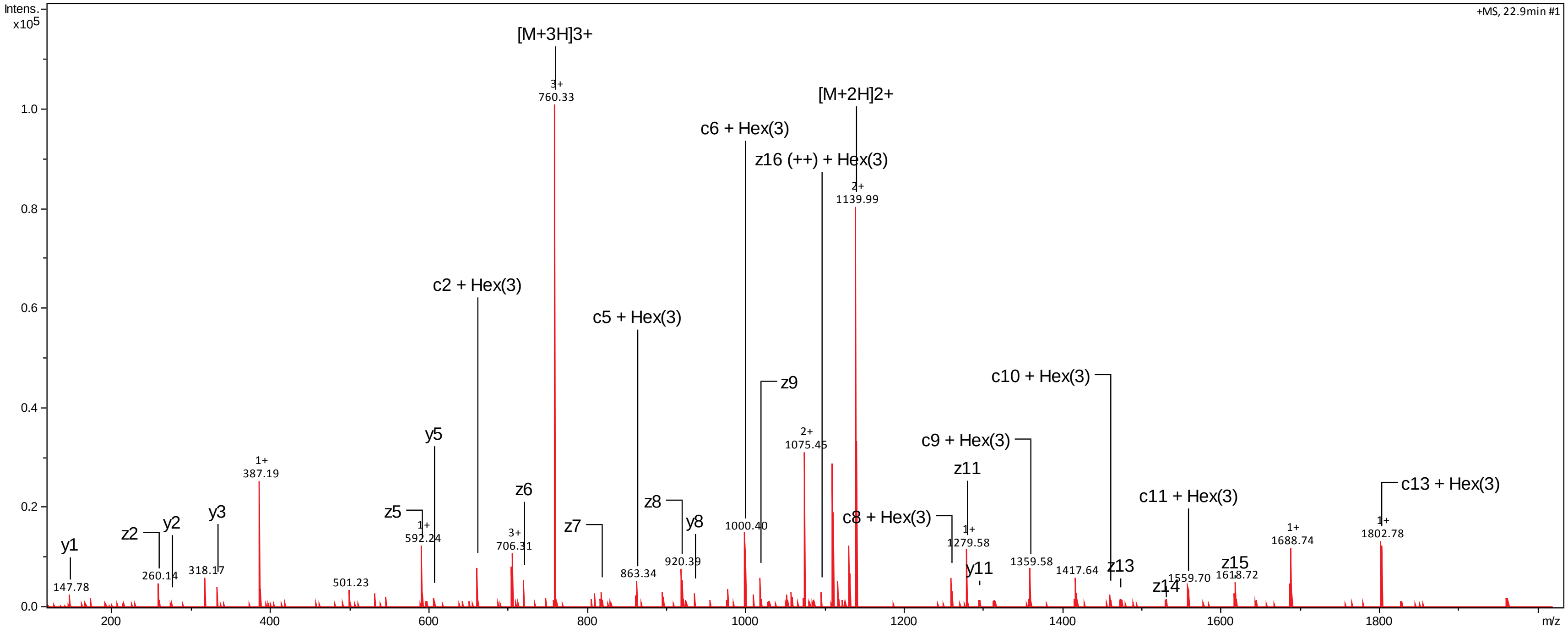

SCO Number	SCO7218
Precursor m/z	724.830
Charge	4
Retention time	49.6
Scan number	11845
Hex on peptide	3
e-value	0.0047
Site allocated?	N
Method	ETD_IT, ETD_OT

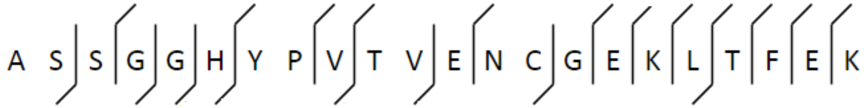

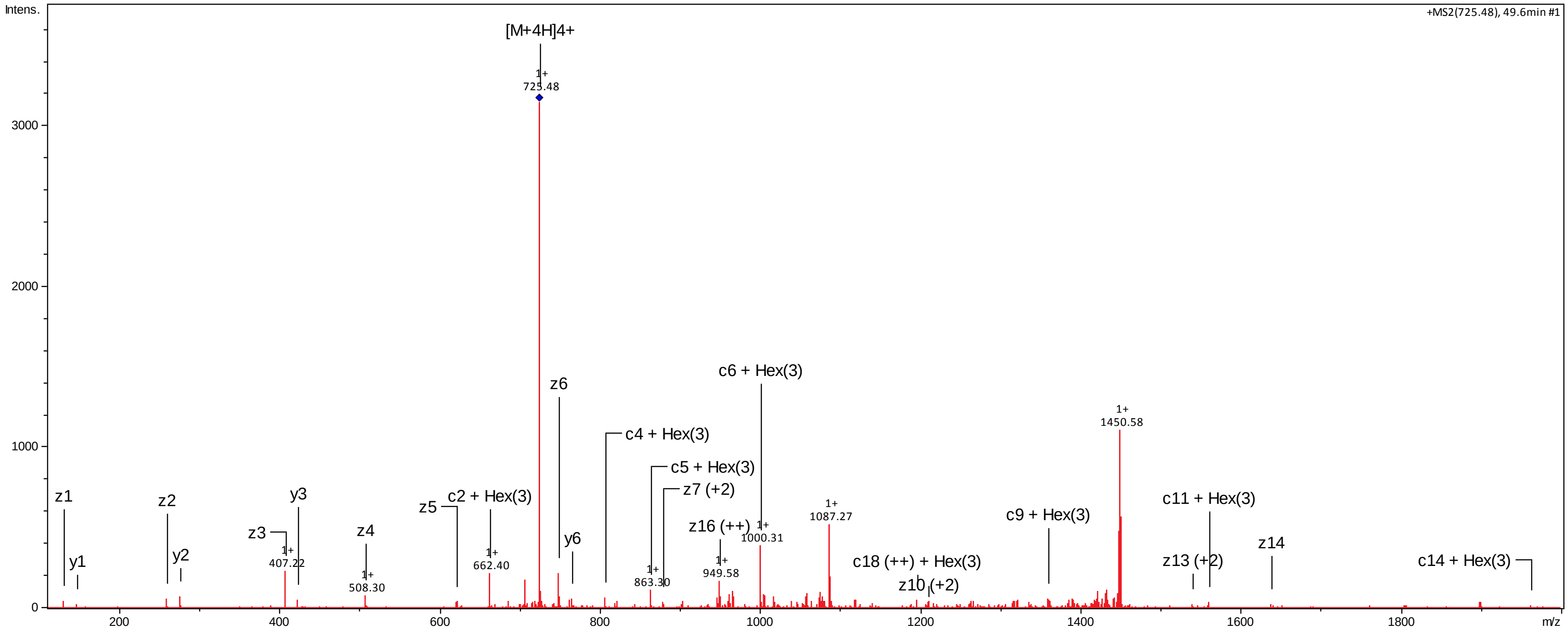

SCO Number	SCO3357
Precursor m/z	814.373
Charge	3
Retention time	17
Scan number	2155
Hex on peptide	6
e-value	0.029
Site allocated?	N
Method	ETD_IT

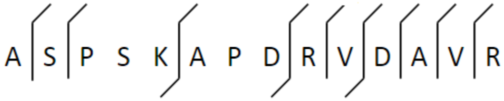

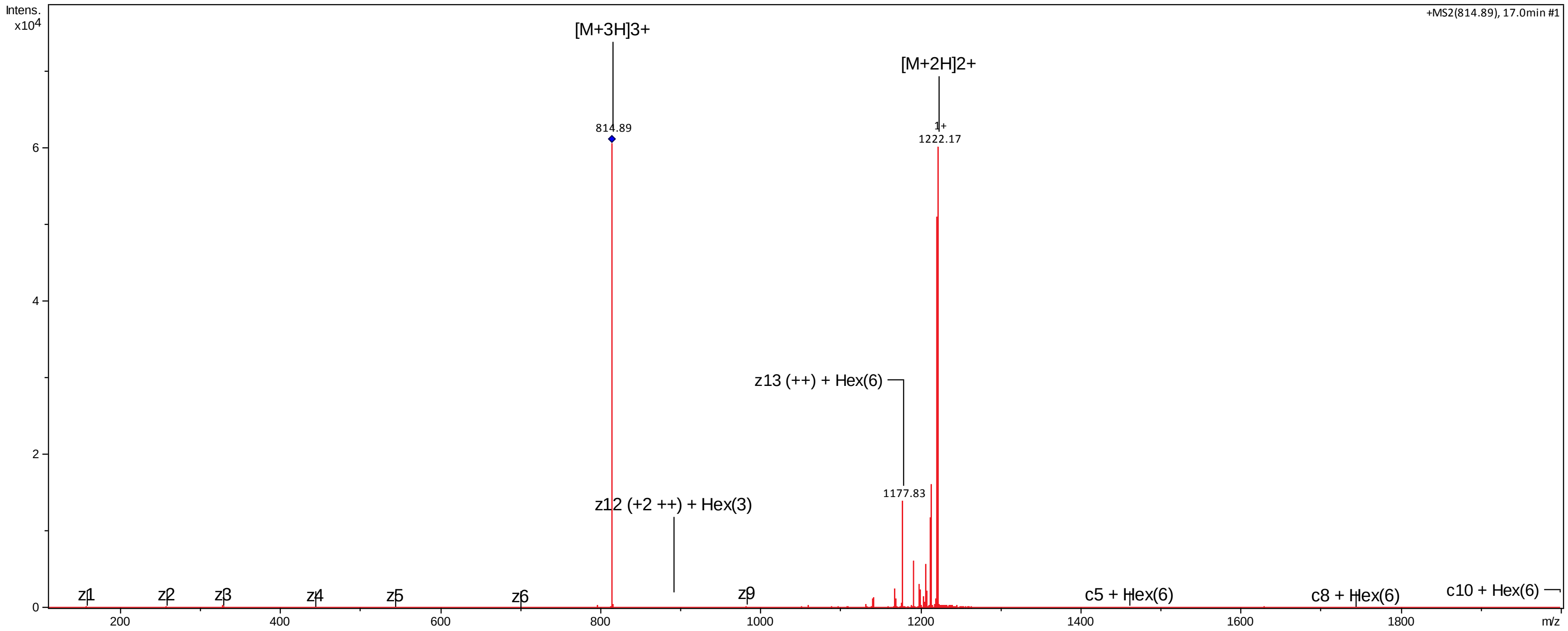

SCO Number	SCO2963
Precursor m/z	541.738
Charge	2
Retention time	39.5
Scan number	8834
Hex on peptide	1
e-value	0.0065
Site allocated?	N
Method	ETD_IT

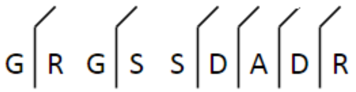

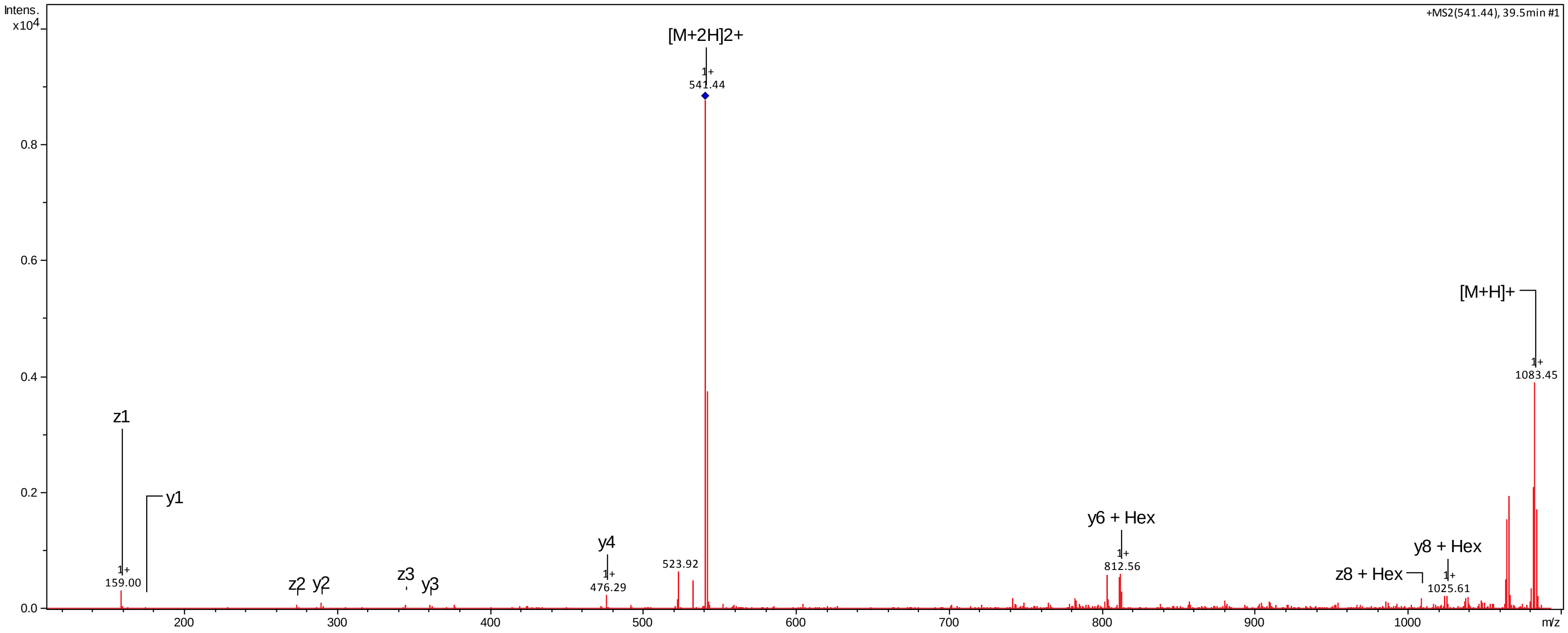

SCO Number	SCO4142
Precursor m/z	739.725
Charge	3
Retention time	71.6
Scan number	32536
Hex on peptide	1
e-value	0.0019
Site allocated?	N
Method	ETD_IT

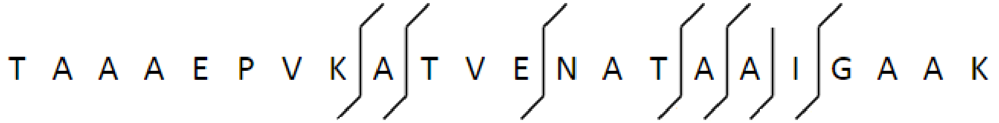

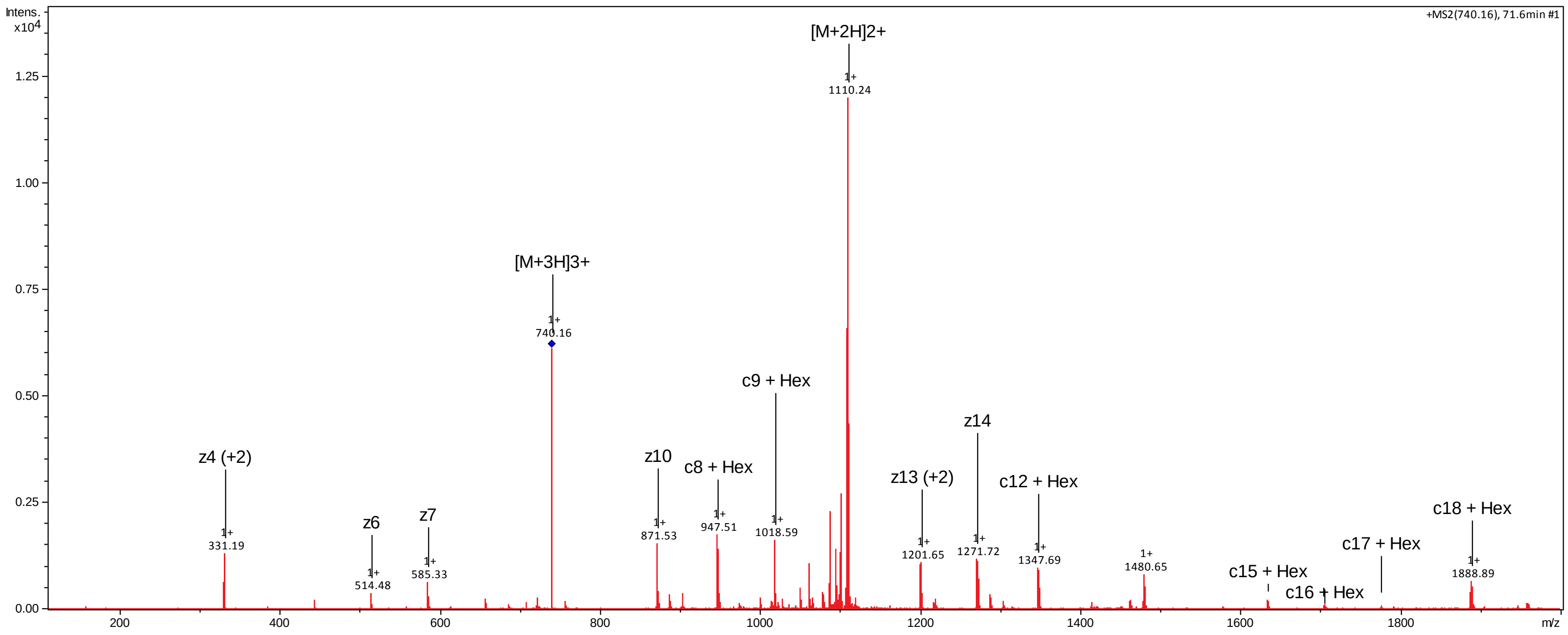

SCO Number	SCO3184
Precursor m/z	694.342
Charge	3
Retention time	23.9
Scan number	46.3
Hex on peptide	1
e-value	0.000011
Site allocated?	N
Method	ETD_IT, ETD_OT

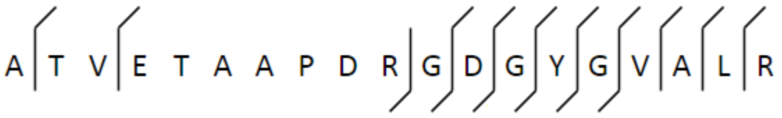

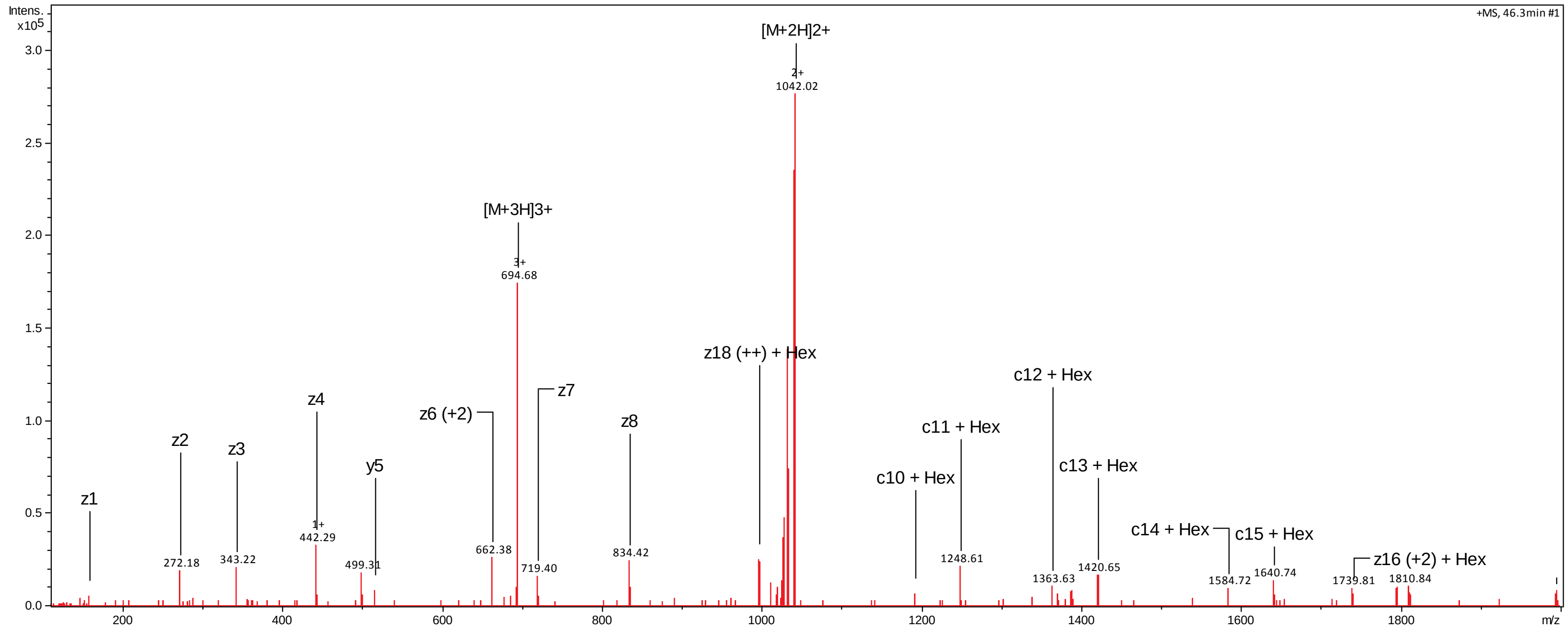

SCO Number	SCO4142
Precursor m/z	956.931
Charge	2
Retention time	17.4
Scan number	4208
Hex on peptide	1
e-value	0.048
Site allocated?	N
Method	HCD_IT

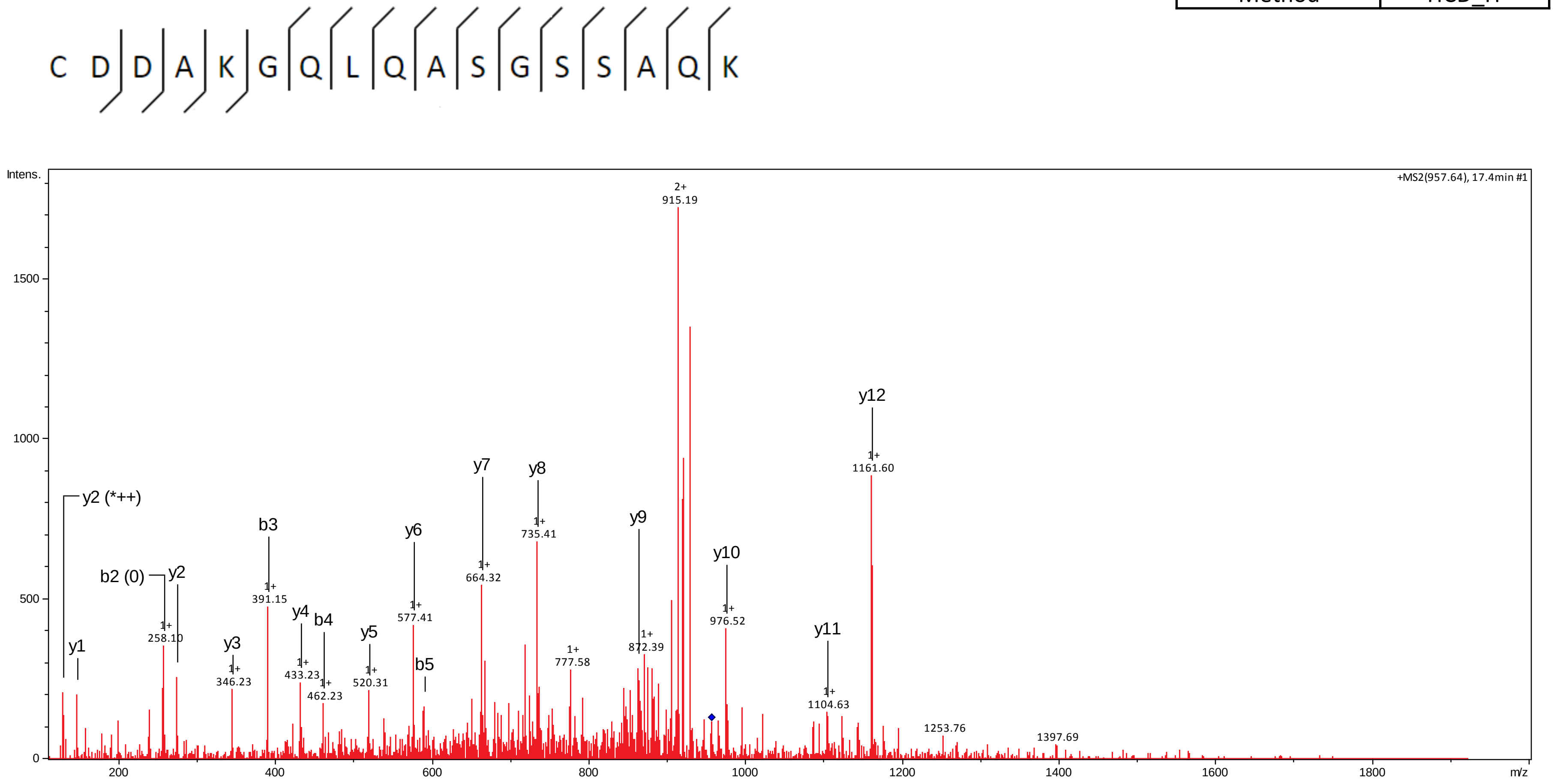

SCO Number	SCO4142
Precursor m/z	1345.343
Charge	3
Retention time	136.9
Scan number	92043
Hex on peptide	1
Other variable mod	Oxidation of M12
e-value	0.014
Site allocated?	N
Method	HCD_IT

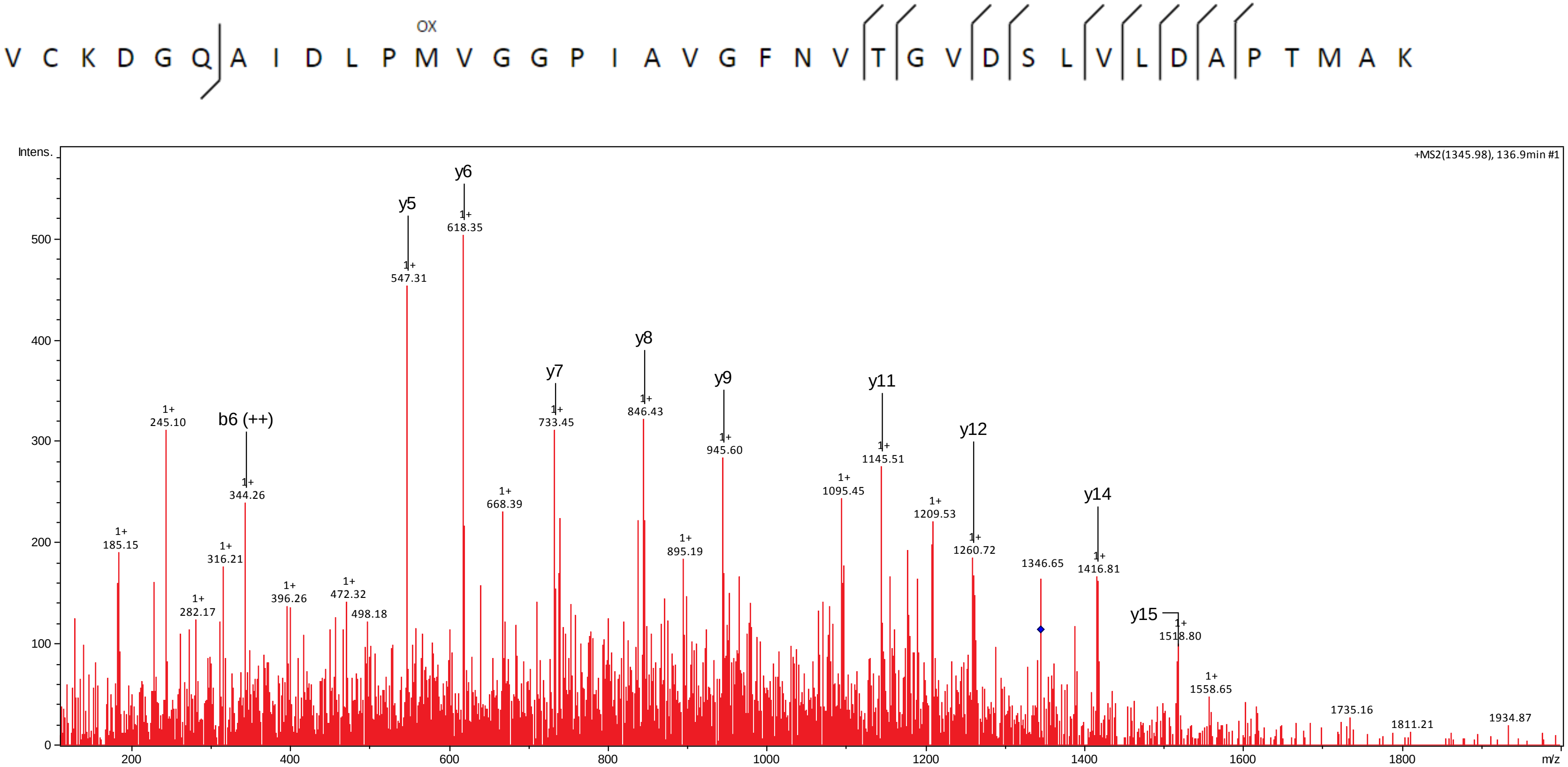

SCO Number	SCO4142
Precursor m/z	1083.868
Charge	3
Retention time	92
Scan number	58627
Hex on peptide	1
e-value	0.000036
Site allocated?	N
Method	HCD_IT

G G Q S A Q G S S G L A G Q V K Q T P G A I S Y F E L S Y A K

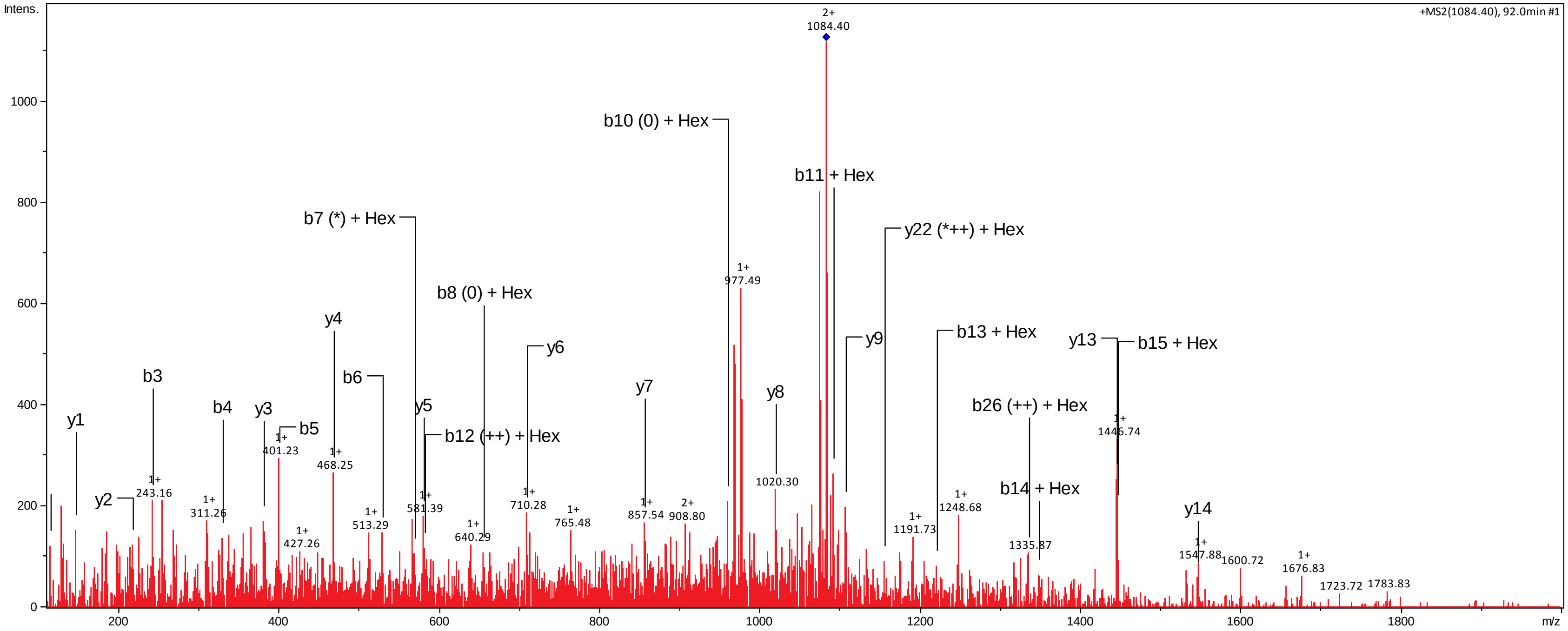

SCO Number	SCO4142
Precursor m/z	1388.343
Charge	3
Retention time	136
Scan number	91188
Hex on peptide	1
e-value	0.00078
Site allocated?	N
Method	HCD_IT

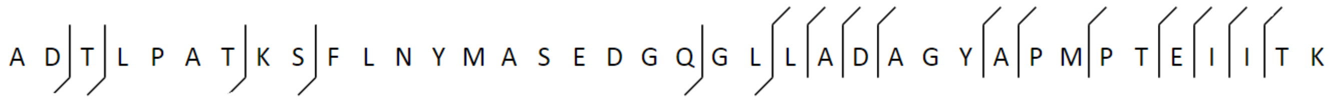

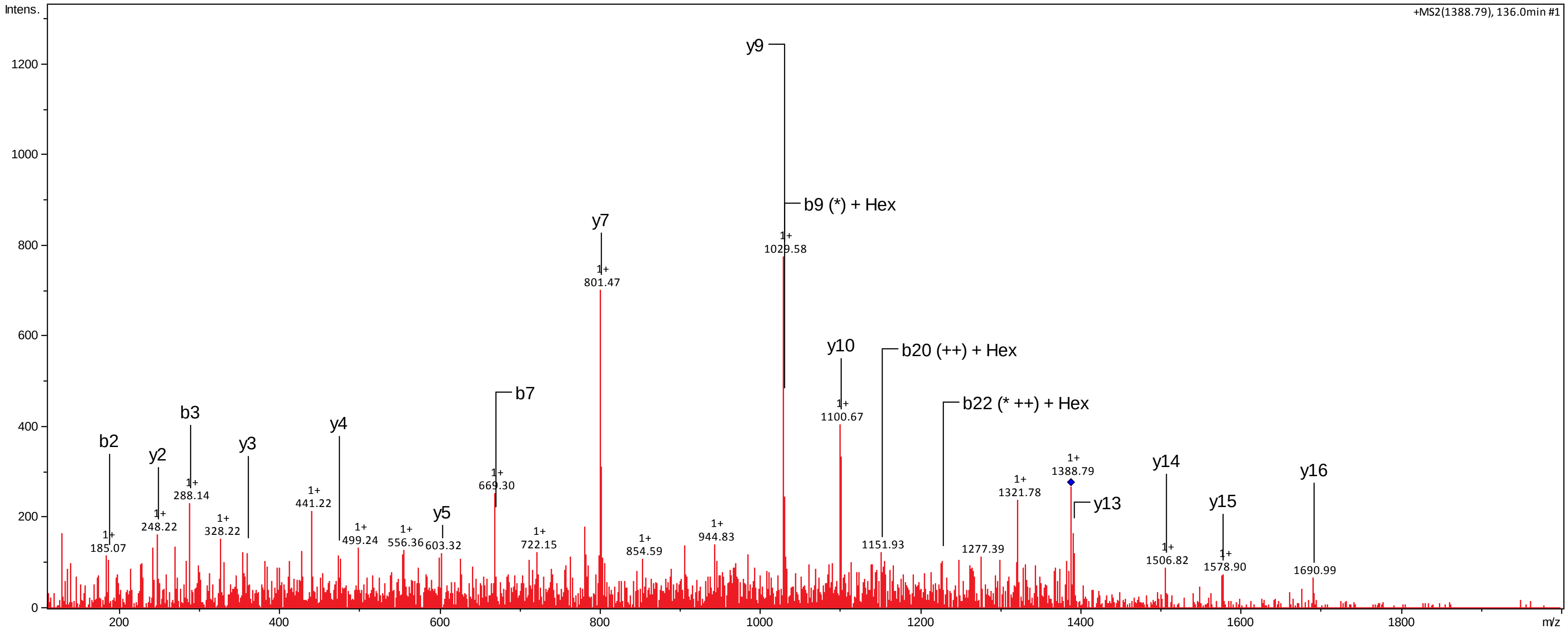

SCO Number	SCO2156
Precursor m/z	733.330
Charge	3
Retention time	48.7
Scan number	25242
Hex on peptide	1
e-value	0.0015
Site allocated?	N
Method	HCD_IT

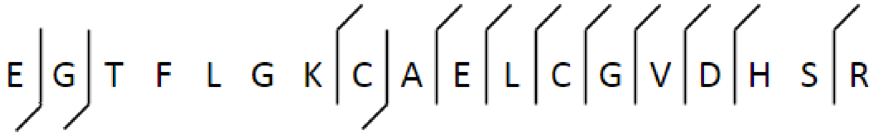

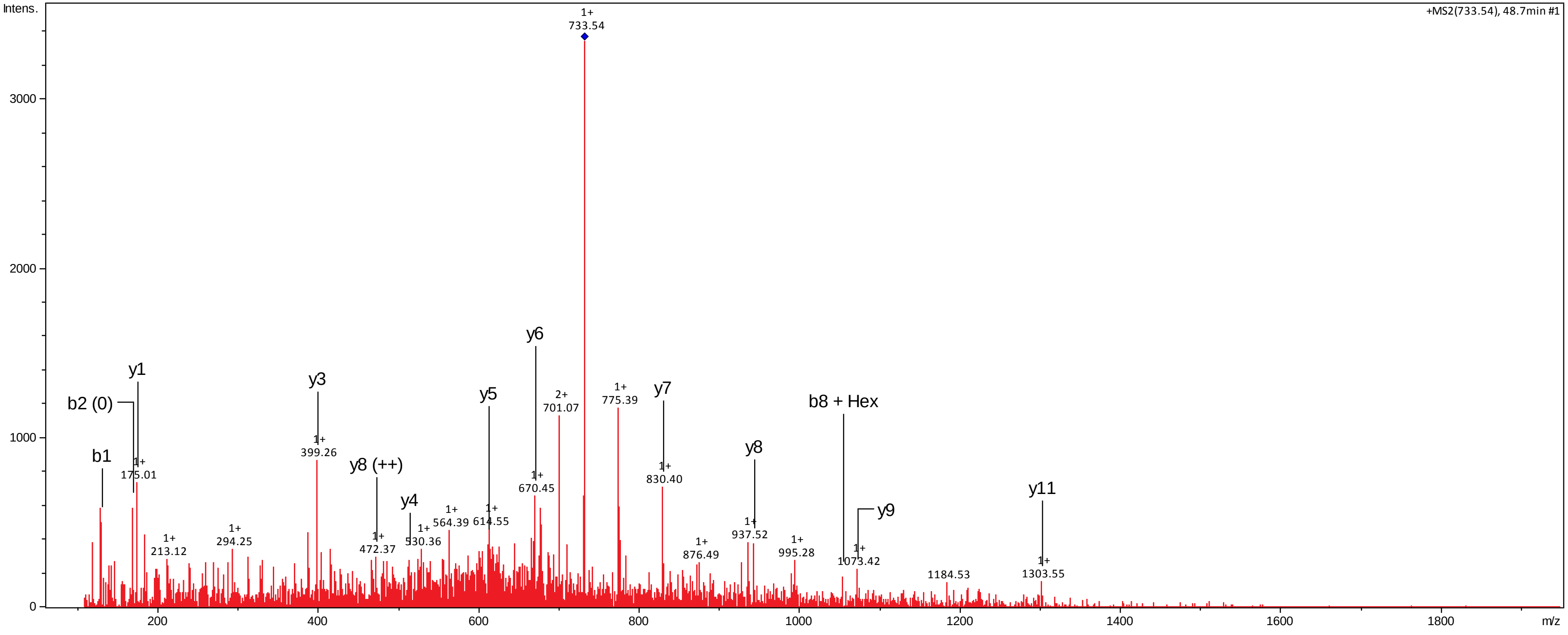

SCO Number	SCO5646
Precursor m/z	894.792
Charge	3
Retention time	109.8
Scan number	72287
Hex on peptide	1
e-value	0.0059
Site allocated?	N
Method	HCD_IT

A I L T K D N P Q G D V F F G V D N T L L S R

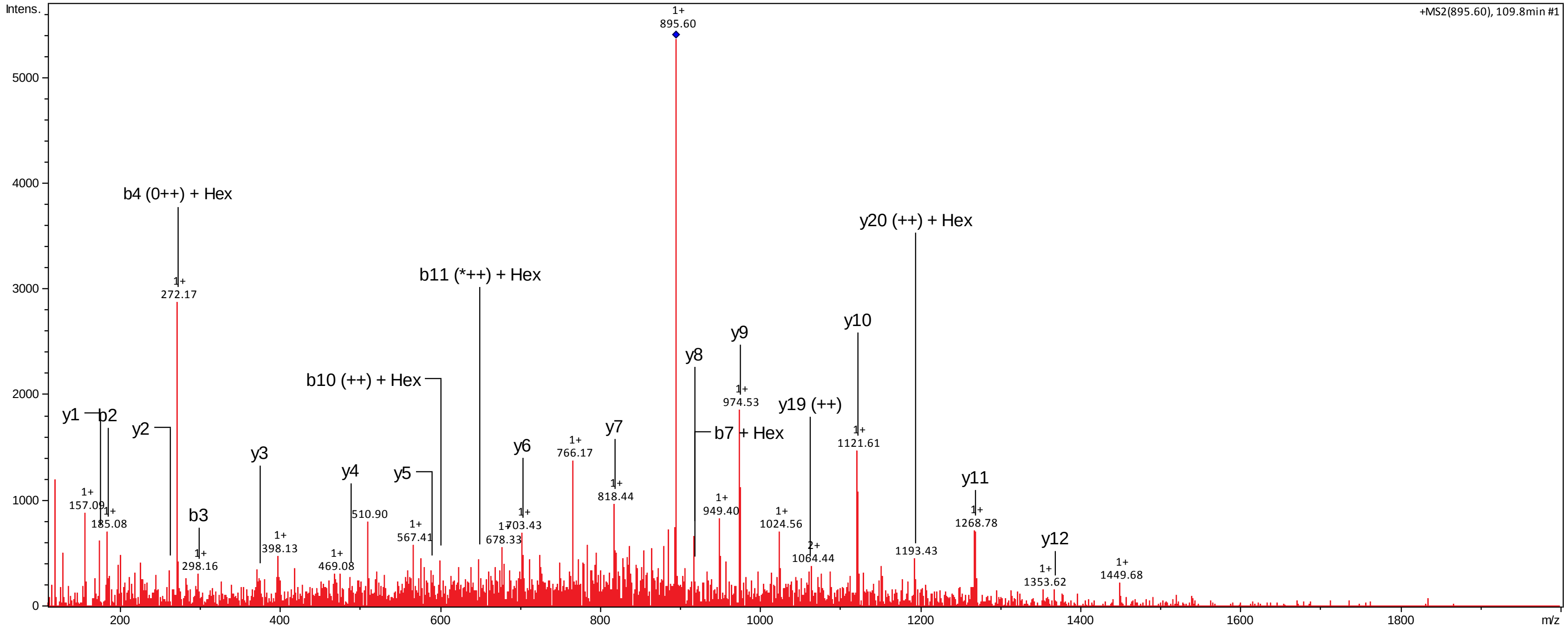

SCO Number	SCO0996
Precursor m/z	753.352
Charge	3
Retention time	76.4
Scan number	46383
Hex on peptide	2
e-value	0.00082
Site allocated?	N
Method	HCD_IT

A T A P S A E G F P V T I D N C G V K

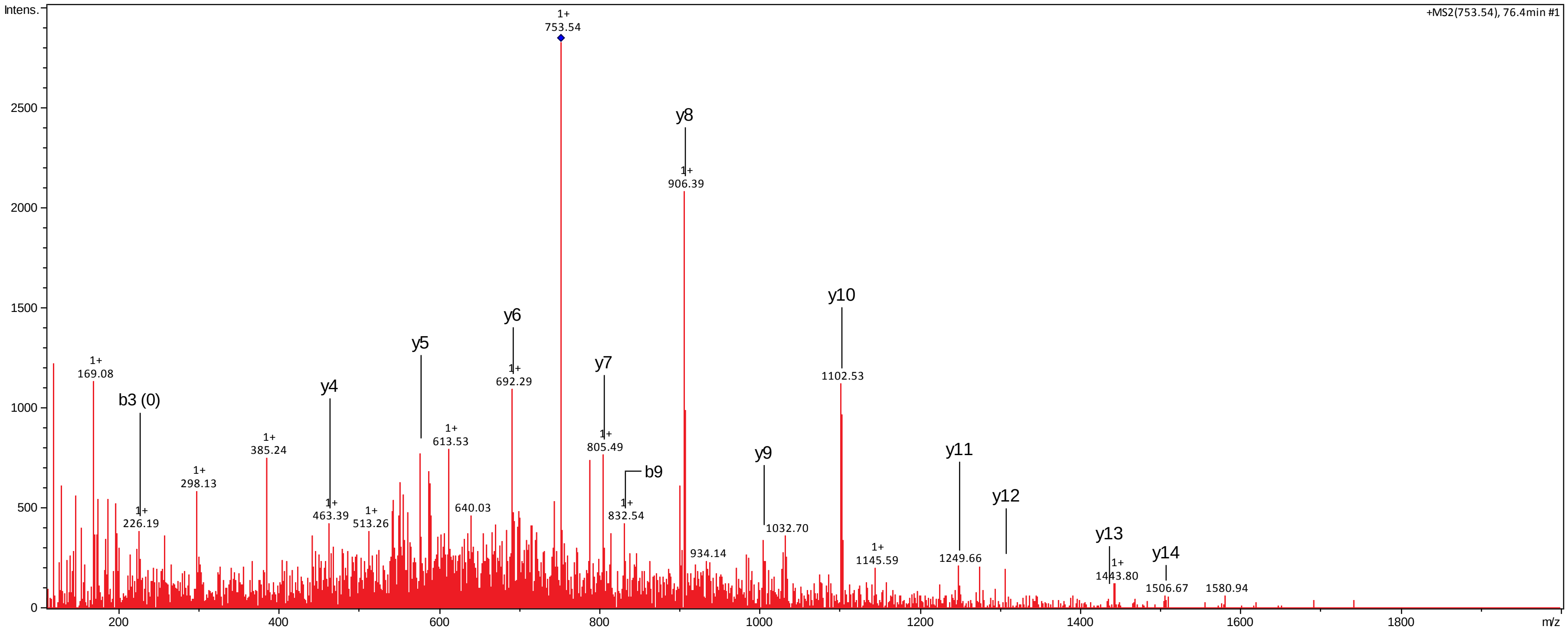

SCO Number	SCO0996
Precursor m/z	1210.550
Charge	2
Retention time	74.7
Scan number	45042
Hex on peptide	3
e-value	0.021
Site allocated?	N
Method	HCD_IT

A T A | P S A | E | G F | P V | T | I | D | N | C G V | K

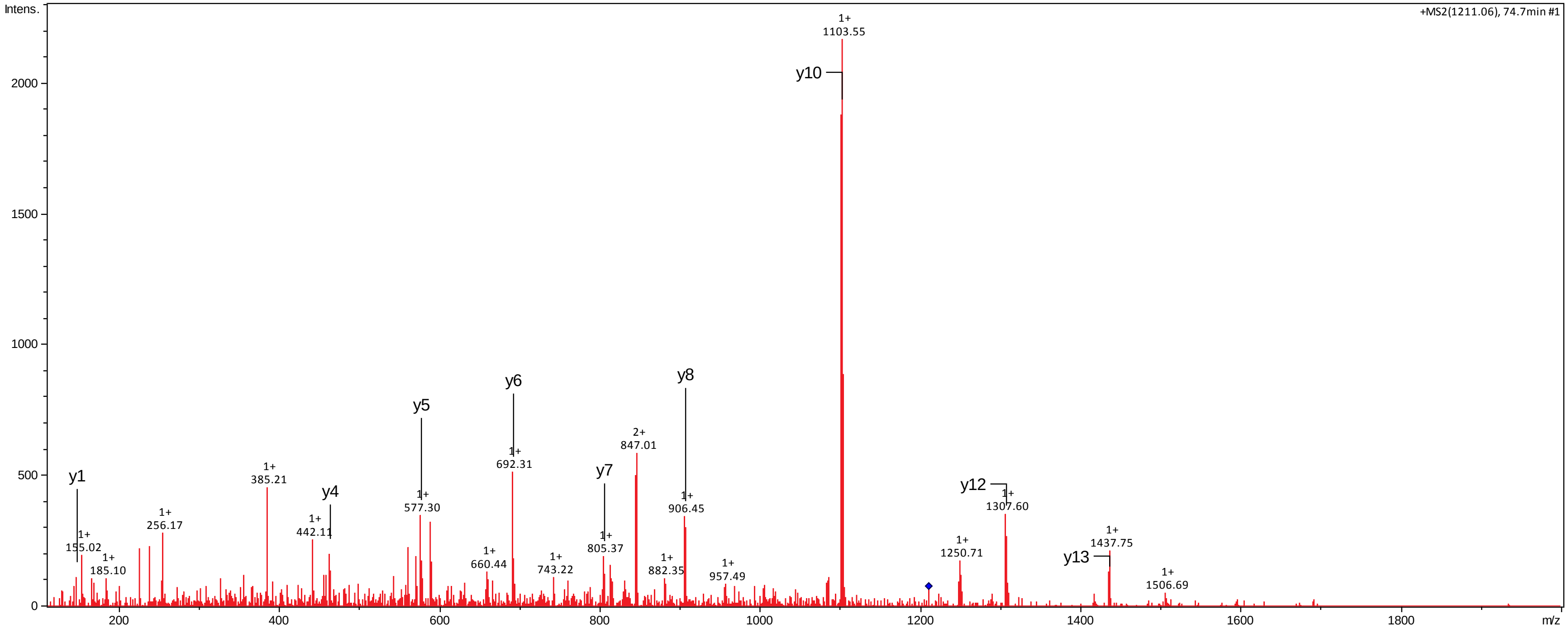

SCO Number	SCO3184
Precursor m/z	737.041
Charge	3
Retention time	35.6
Scan number	16323
Hex on peptide	1
e-value	0.0014
Site allocated?	N
Method	HCD_IT

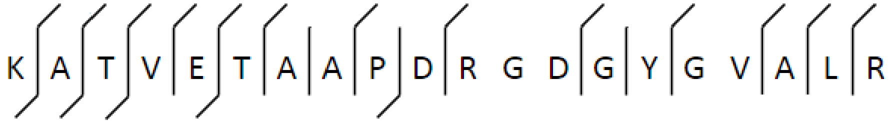

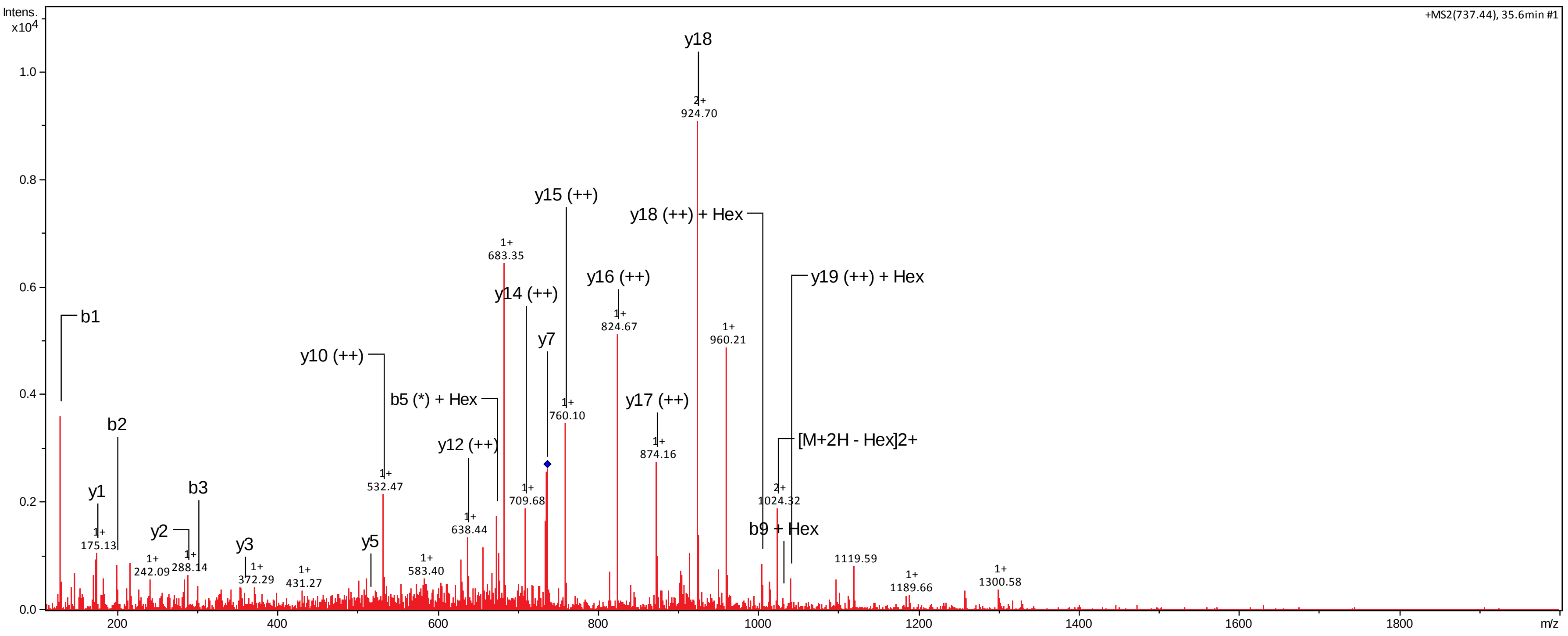

SCO Number	SCO4013
Precursor m/z	890.437
Charge	2
Retention time	54.1
Scan number	29320
Hex on peptide	1
e-value	0.023
Site allocated?	N
Method	HCD_IT

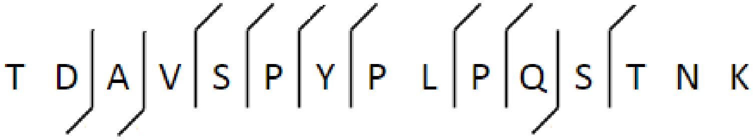

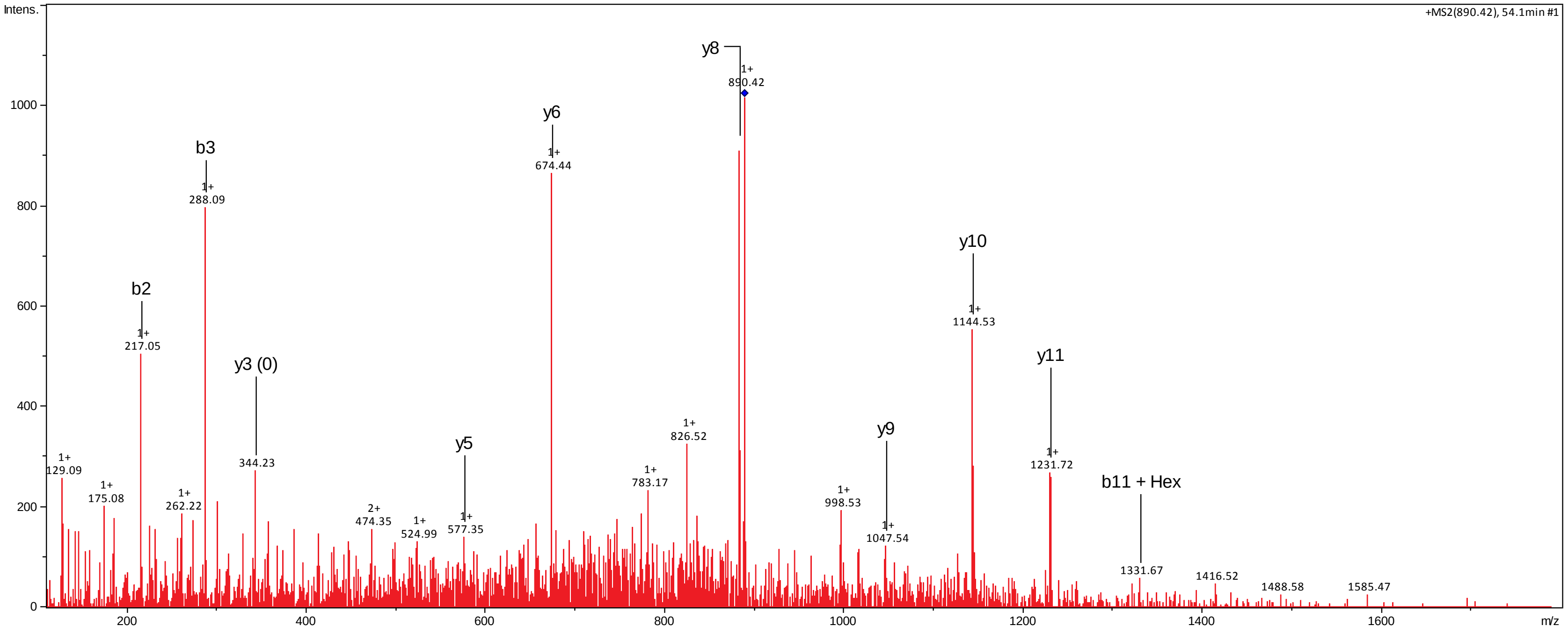

SCO Number	SCO4885
Precursor m/z	1180.552
Charge	2
Retention time	109.3
Scan number	71923
Hex on peptide	1
e-value	0.00000081
Site allocated?	N
Method	HCD_IT

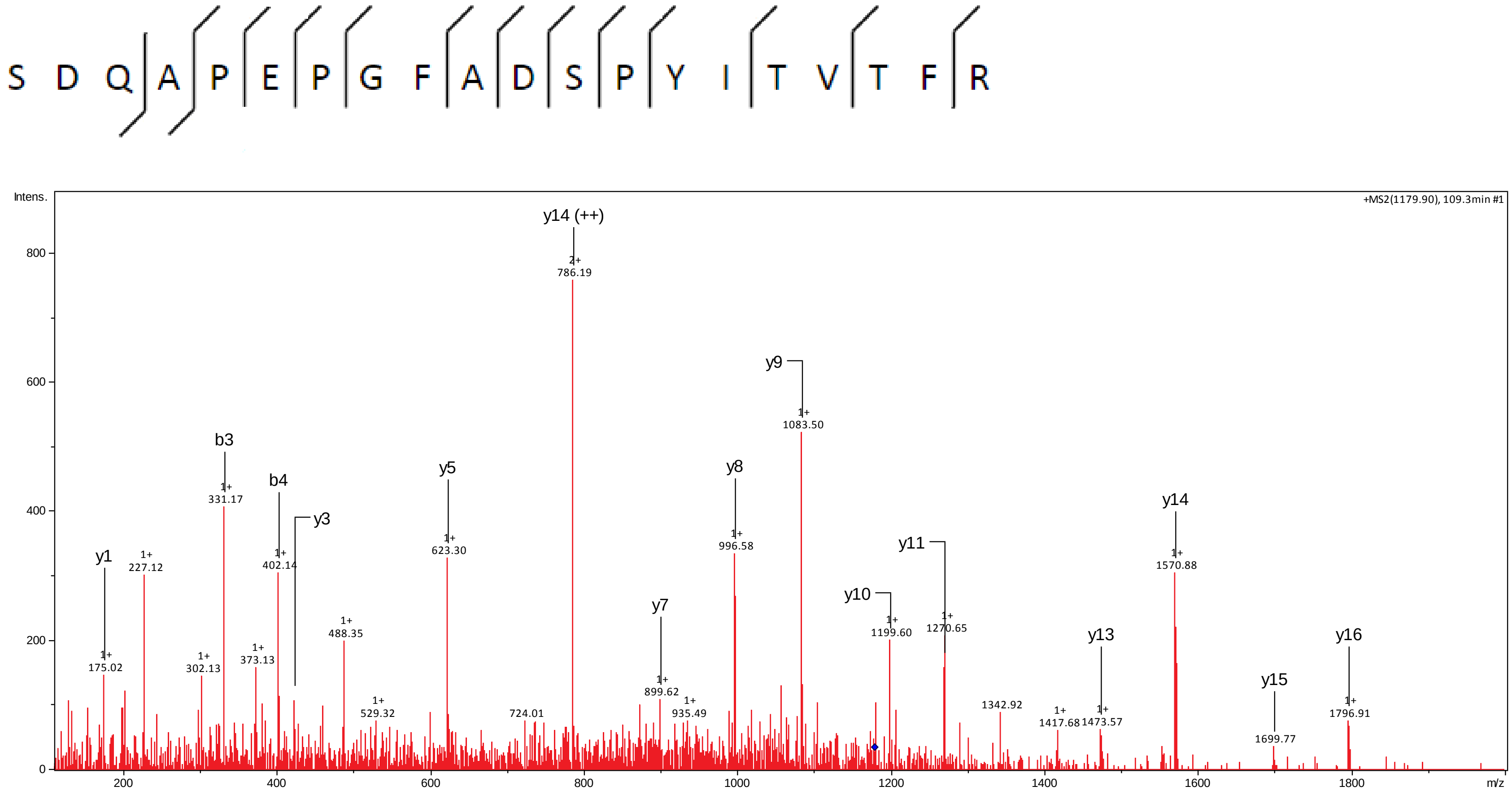

SCO Number	SCO3044
Precursor m/z	995.119
Charge	3
Retention time	98.4
Scan number	63503
Hex on peptide	3
e-value	0.000032
Site allocated?	N
Method	HCD_IT

G D A G Q P S D E P A A D S E I G V L V Q N A T R

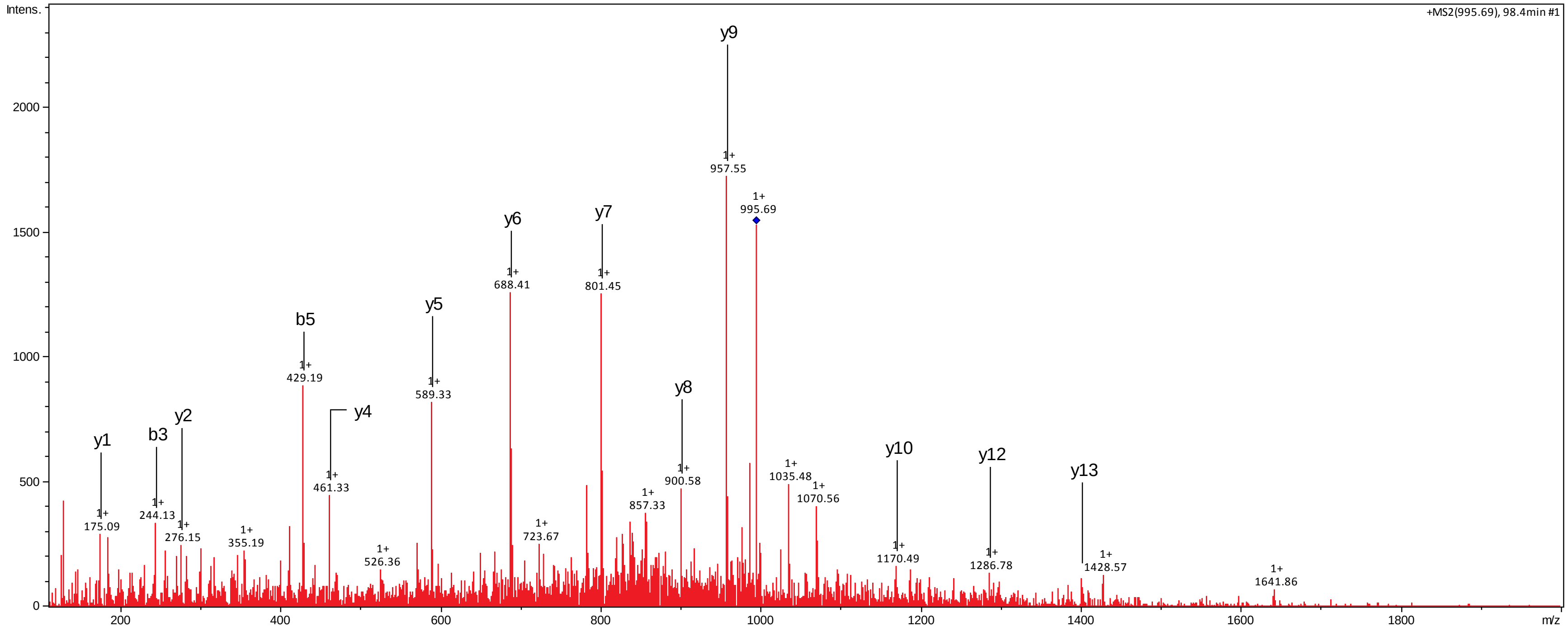

SCO Number	SCO3540
Precursor m/z	954.489
Charge	2
Retention time	57.4
Scan number	31744
Hex on peptide	2
e-value	0.0002
Site allocated?	N
Method	HCD_IT

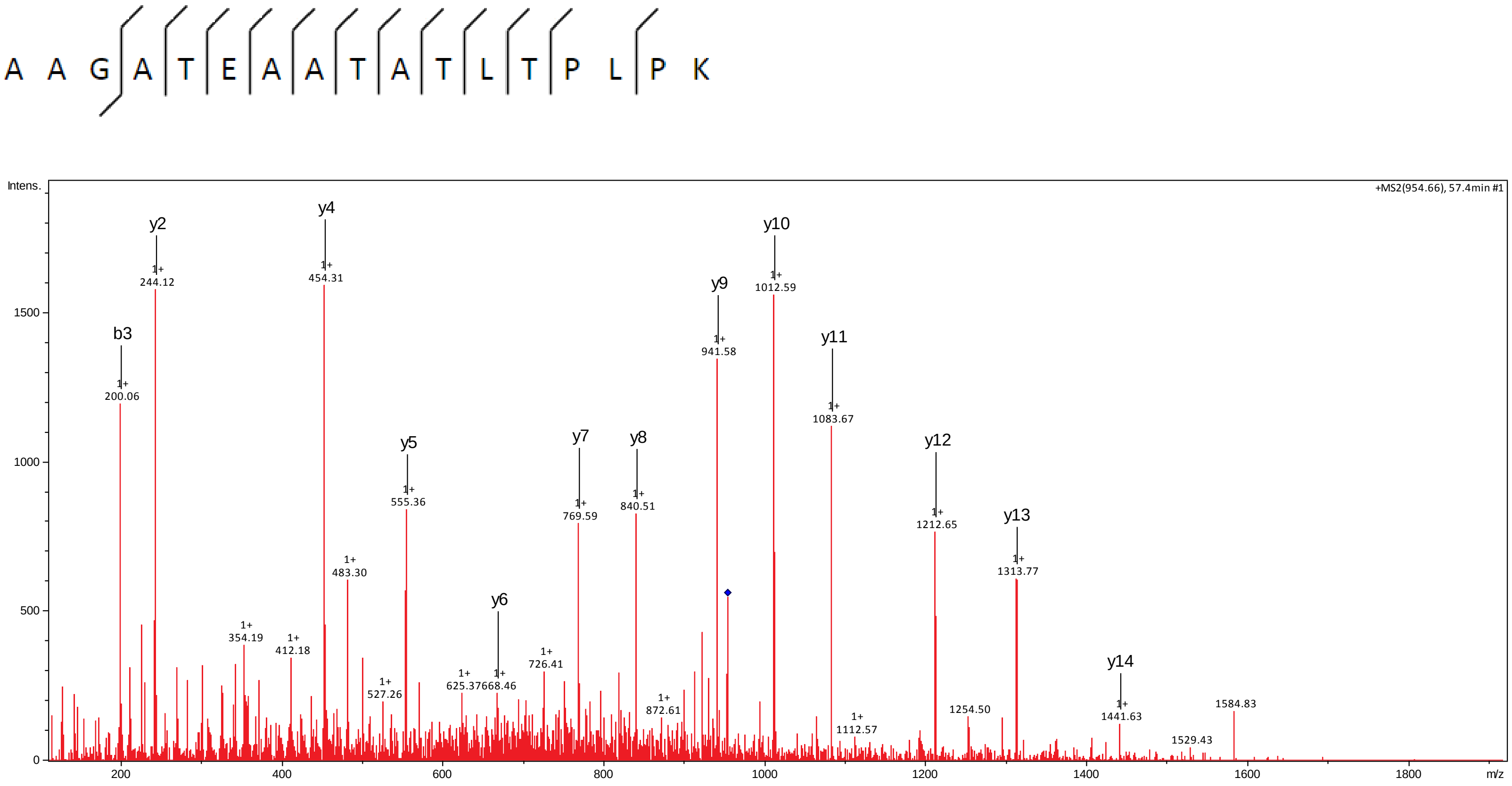

SCO Number	SCO3540
Precursor m/z	1035.515
Charge	2
Retention time	50.7
Scan number	26747
Hex on peptide	3
e-value	0.000022
Site allocated?	N
Method	HCD_IT

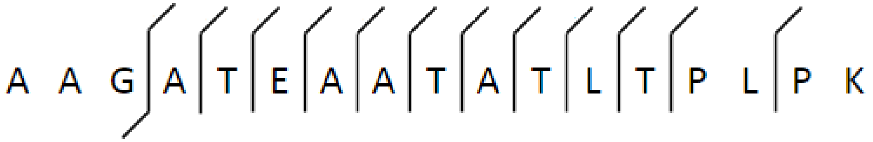

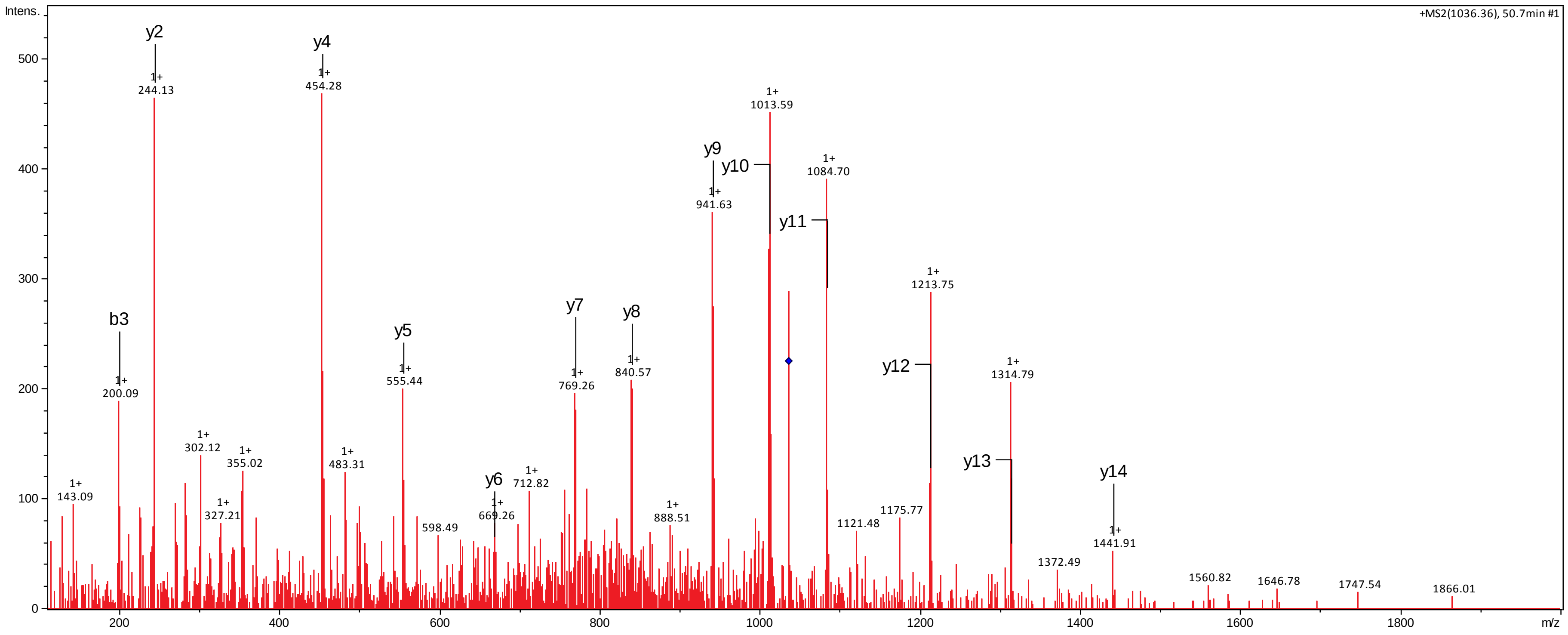

SCO Number	SCO3540
Precursor m/z	910.920
Charge	2
Retention time	46.3
Scan number	23511
Hex on peptide	2
e-value	0.00039
Site allocated?	N
Method	HCD_IT

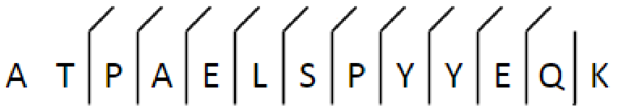

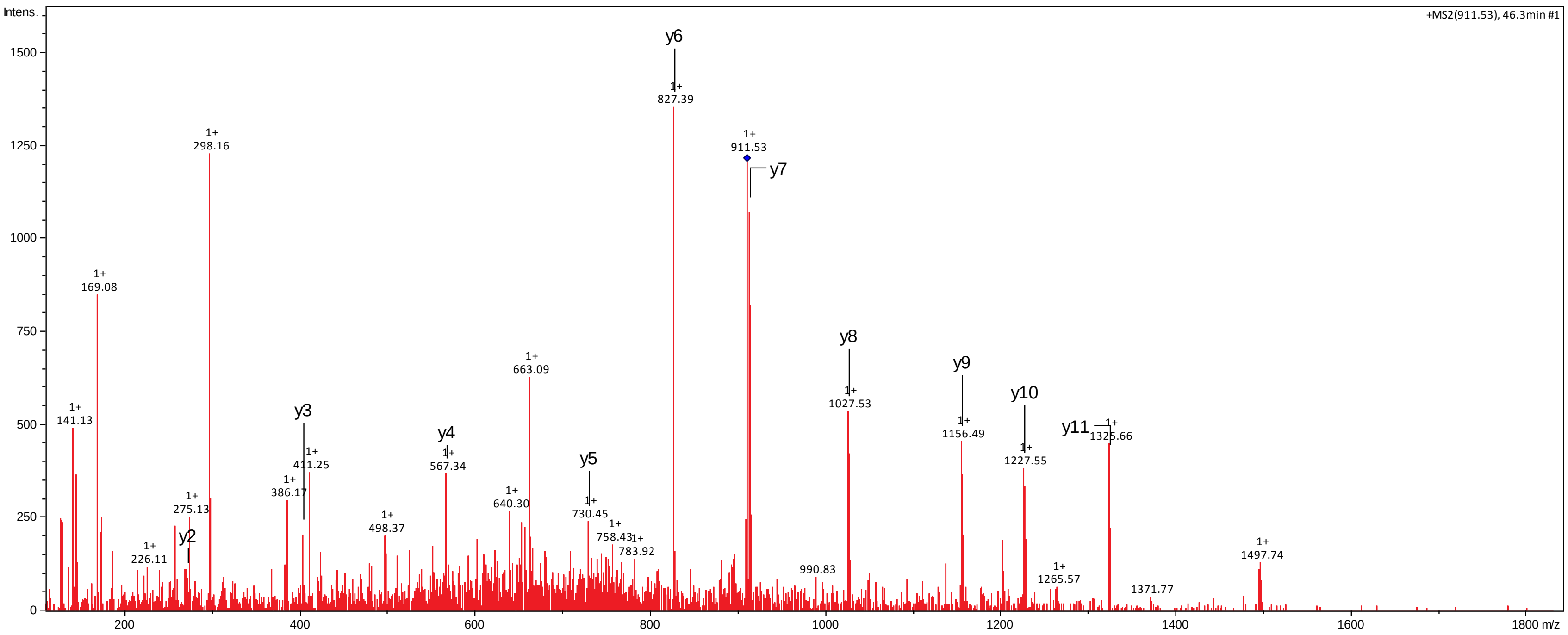

SCO Number	SCO2096
Precursor m/z	909.421
Charge	3
Retention time	51
Scan number	26945
Hex on peptide	3
e-value	0.0059
Site allocated?	N
Method	HCD_IT

K L D A C P N E S A V A V P V T G D D G P K

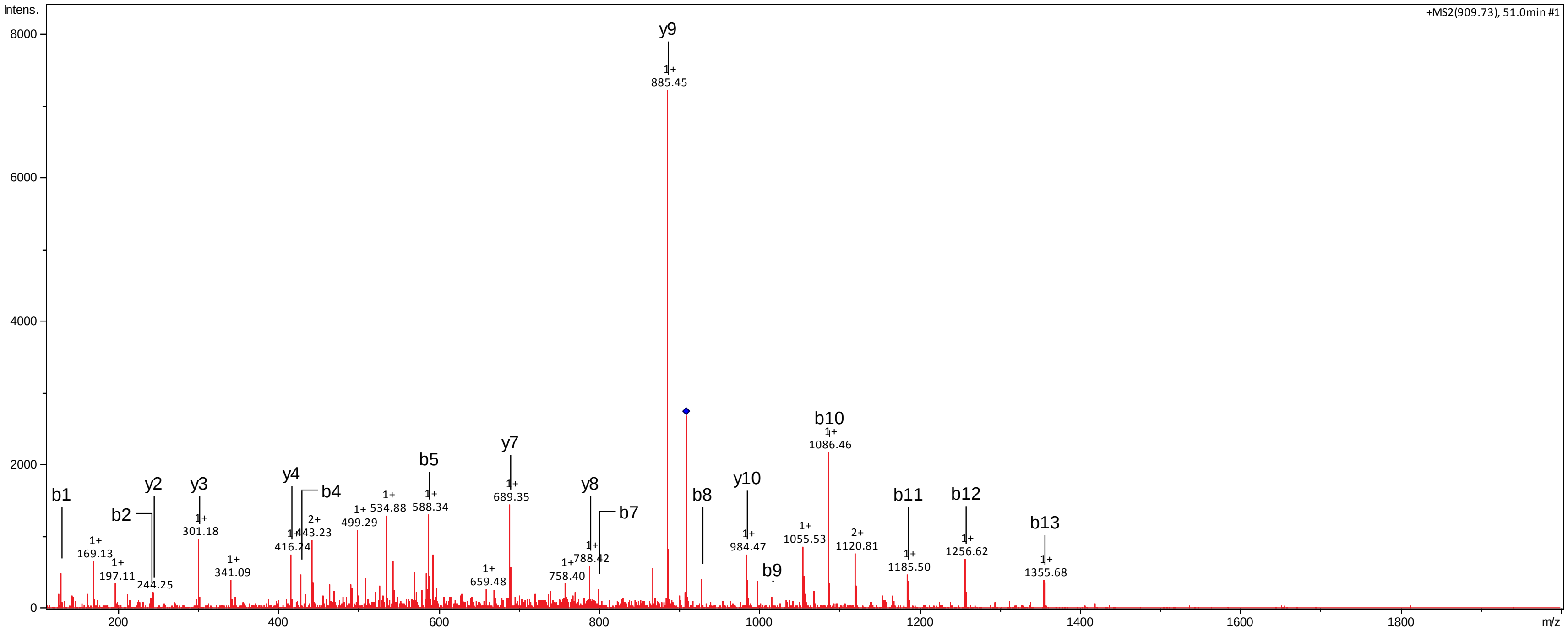

SCO Number	SCO2035
Precursor m/z	1026.959
Charge	2
Retention time	32.4
Scan number	14239
Hex on peptide	2
e-value	0.0066
Site allocated?	N
Method	HCD_IT

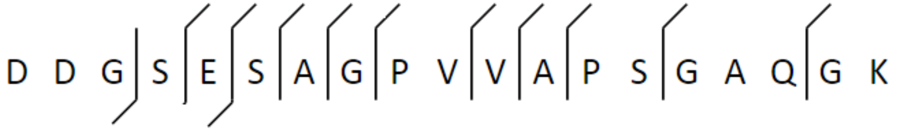

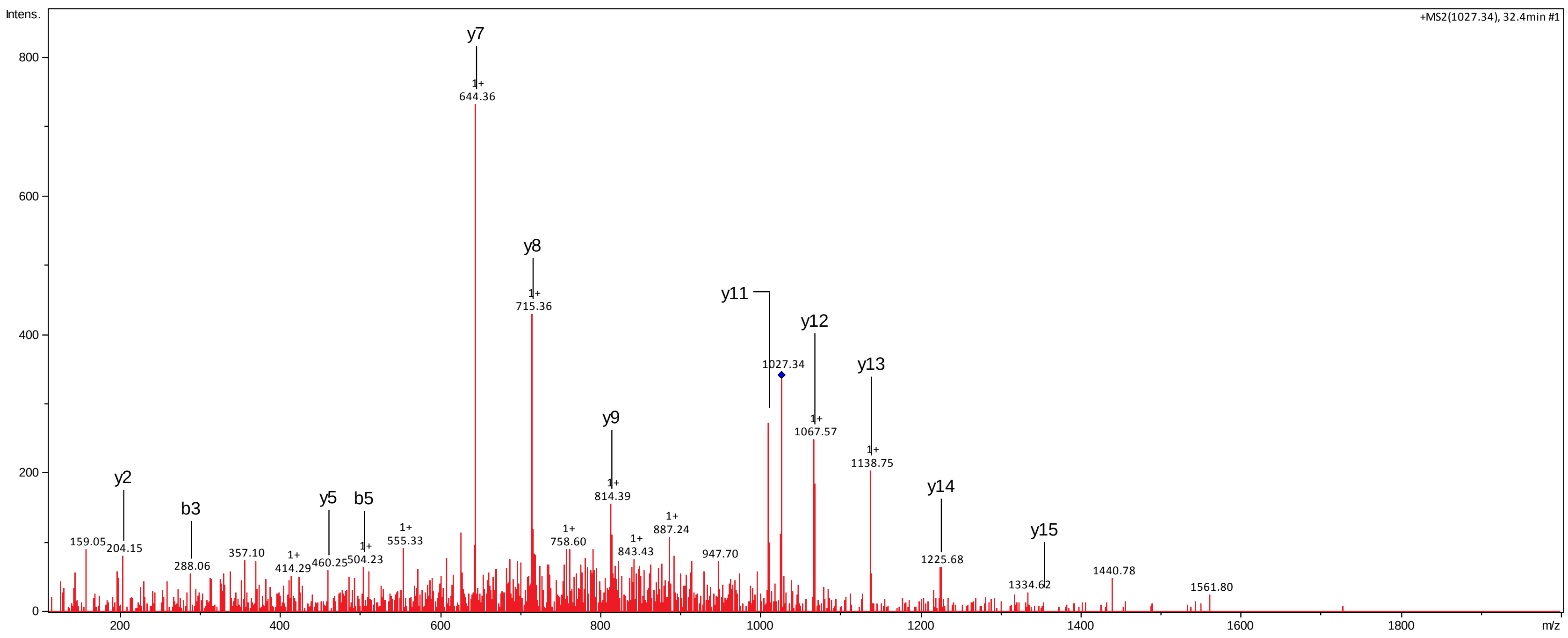

SCO Number	SCO3848
Precursor m/z	769.725
Charge	3
Retention time	53.3
Scan number	28662
Hex on peptide	1
e-value	0.00047
Site allocated?	N
Method	HCD_IT

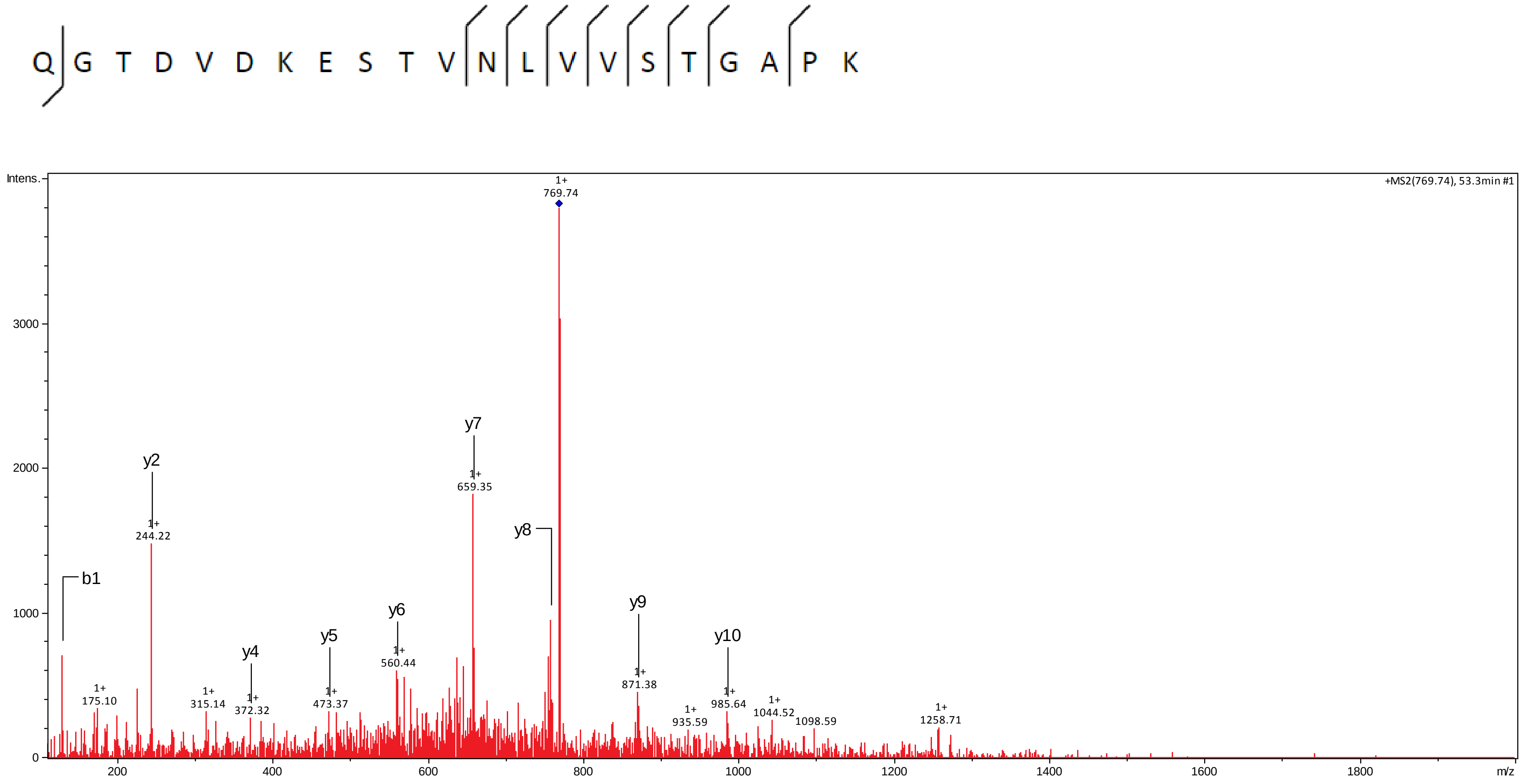

SCO Number	SCO3848
Precursor m/z	823.741
Charge	3
Retention time	52.2
Scan number	27876
Hex on peptide	2
e-value	0.00053
Site allocated?	N
Method	HCD_IT

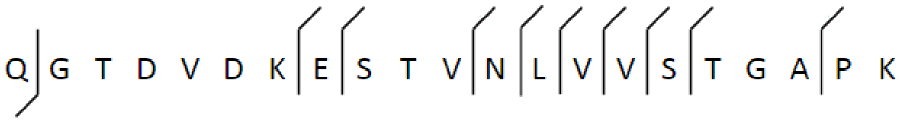

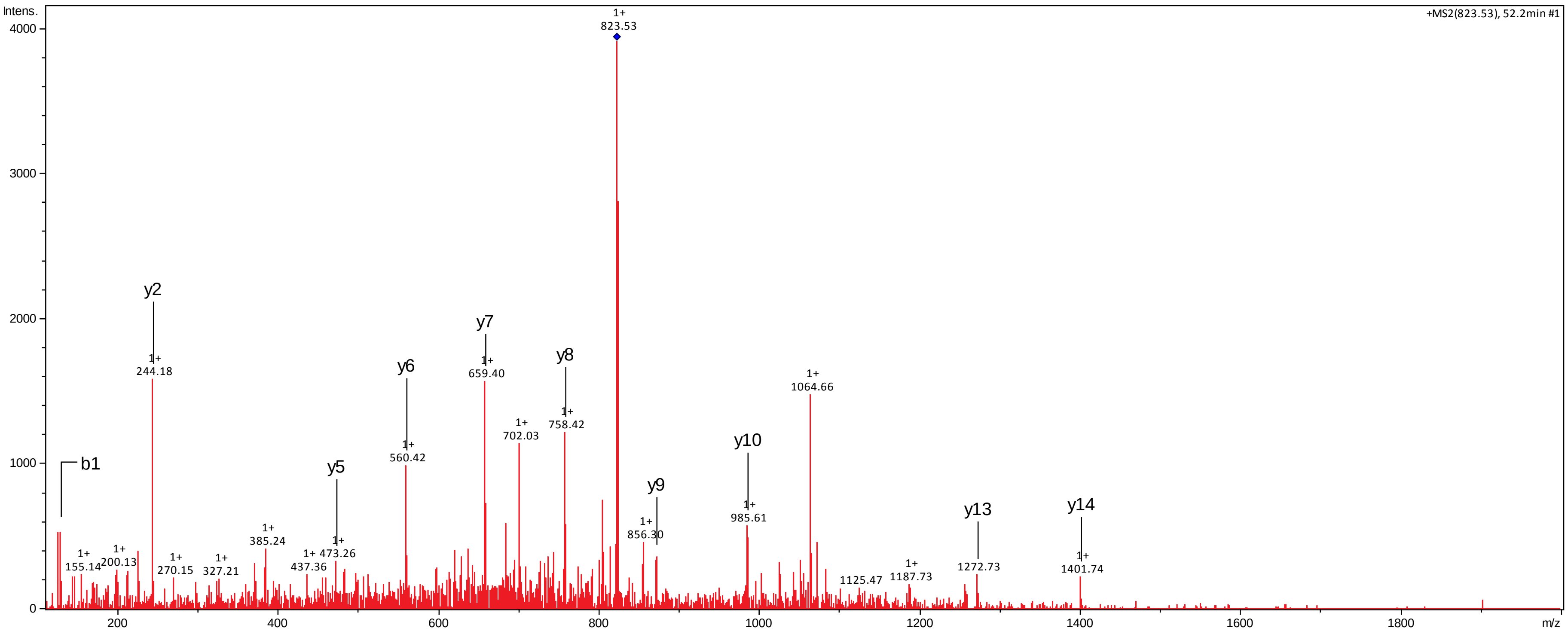

SCO Number	SCO4130
Precursor m/z	1109.514
Charge	4
Retention time	91.9
Scan number	58511
Hex on peptide	2
e-value	0.00036
Site allocated?	N
Method	HCD_IT

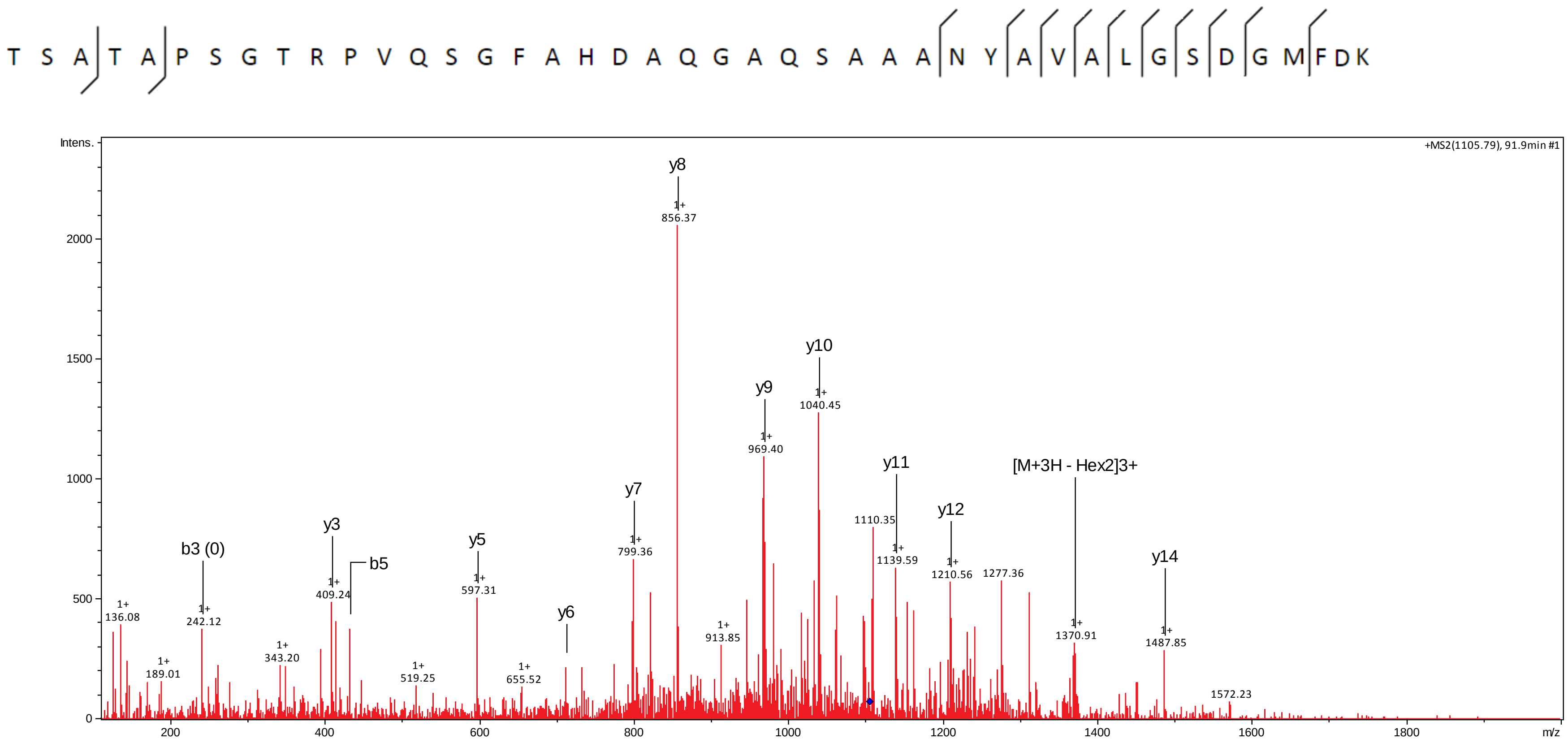

SCO Number	SCO4905
Precursor m/z	919.795
Charge	3
Retention time	132.6
Scan number	4335
Hex on peptide	3
e-value	0.024
Site allocated?	N
Method	HCD_IT

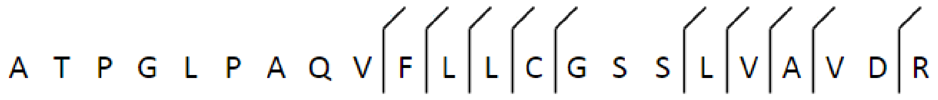

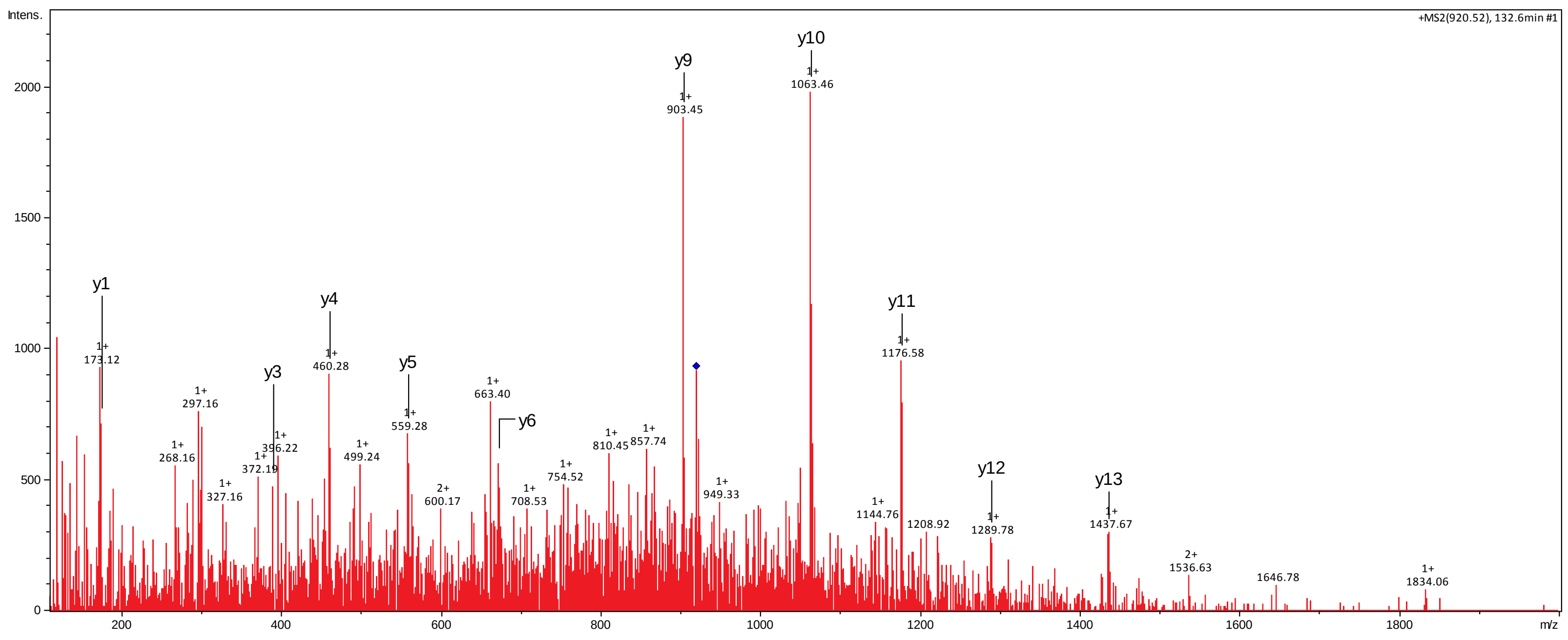

SCO Number	SCO4548
Precursor m/z	877.408
Charge	2
Retention time	20.4
Scan number	6204
Hex on peptide	3
e-value	0.00042
Site allocated?	N
Method	HCD_IT

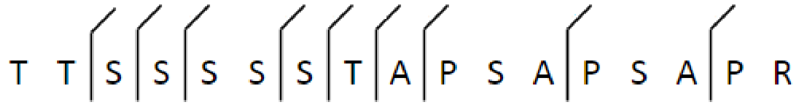

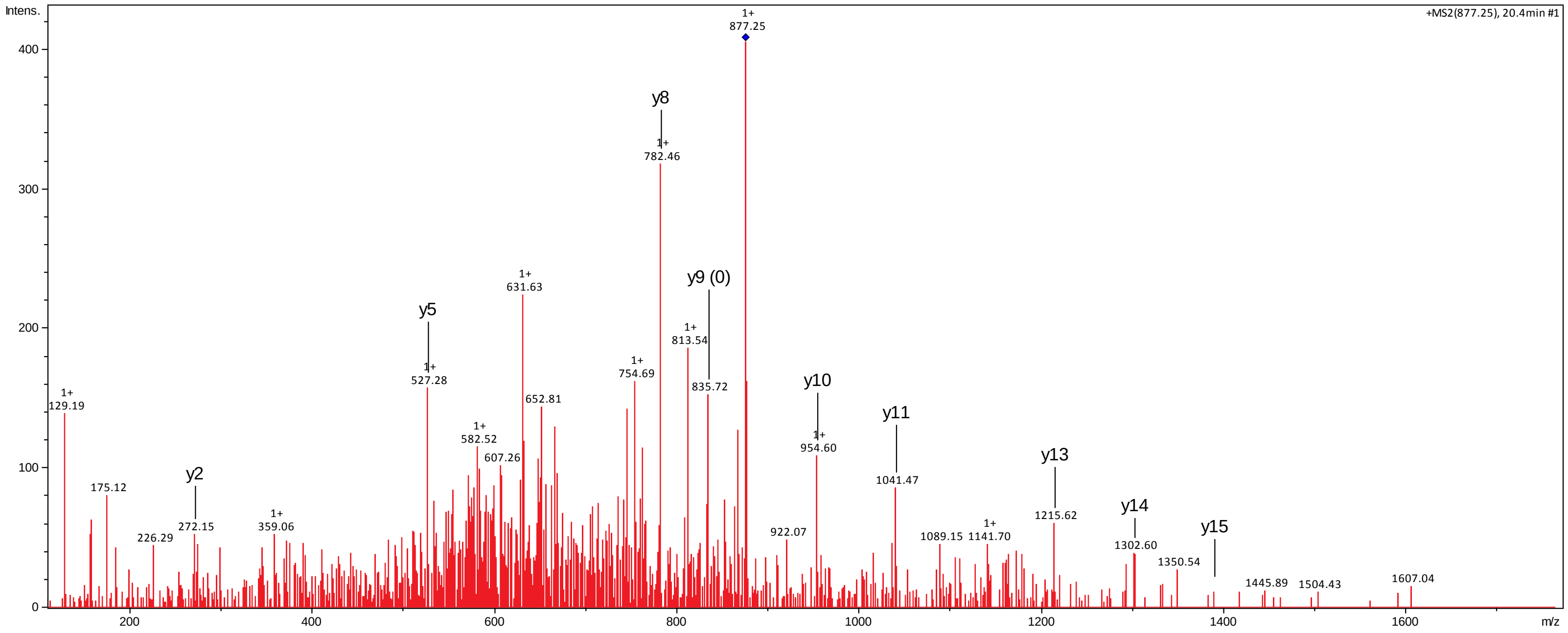

SCO Number	SCO3357
Precursor m/z	933.099
Charge	3
Retention time	50.1
Scan number	26320
Hex on peptide	3
e-value	0.0023
Site allocated?	N
Method	HCD_IT

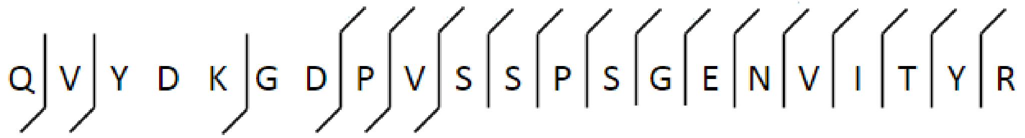

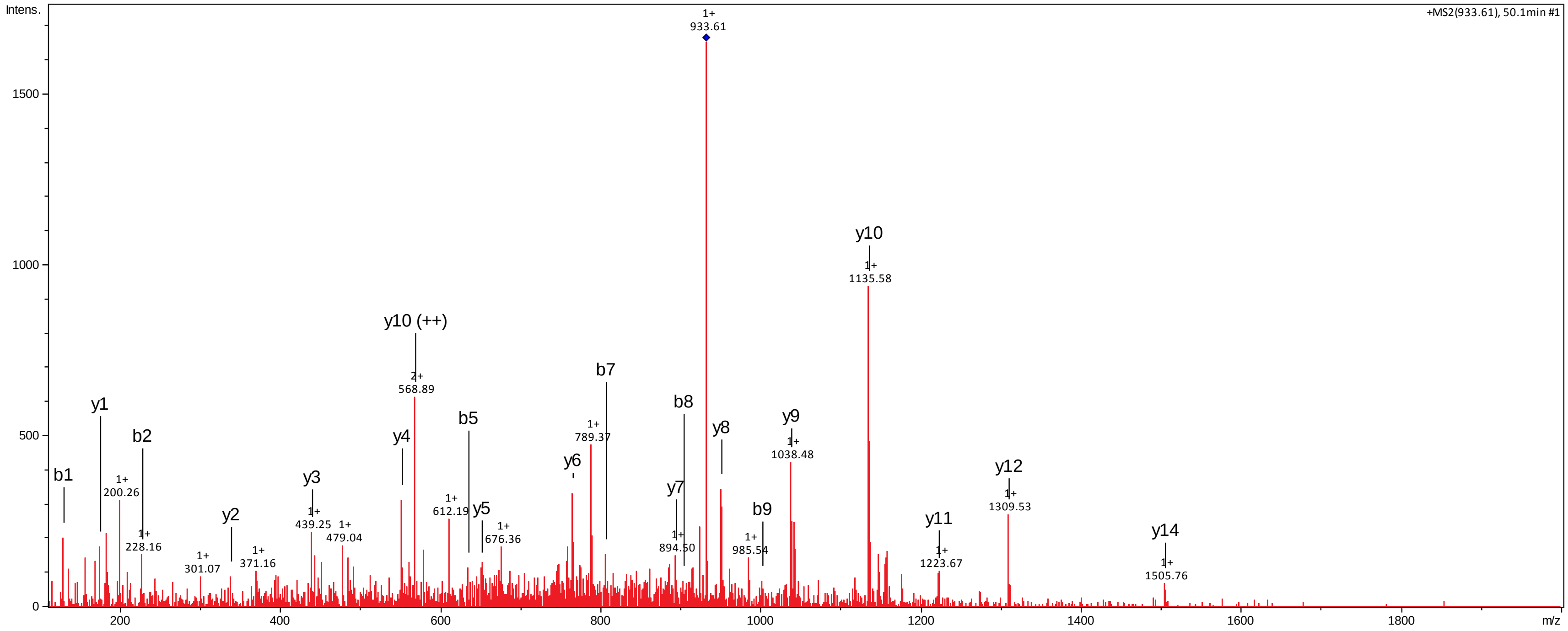

SCO Number	SCO3891
Precursor m/z	938.921
Charge	2
Retention time	45.6
Scan number	22941
Hex on peptide	2
e-value	0.00091
Site allocated?	N
Method	HCD_IT

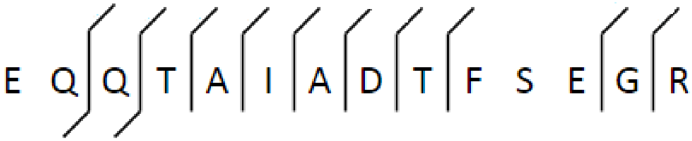

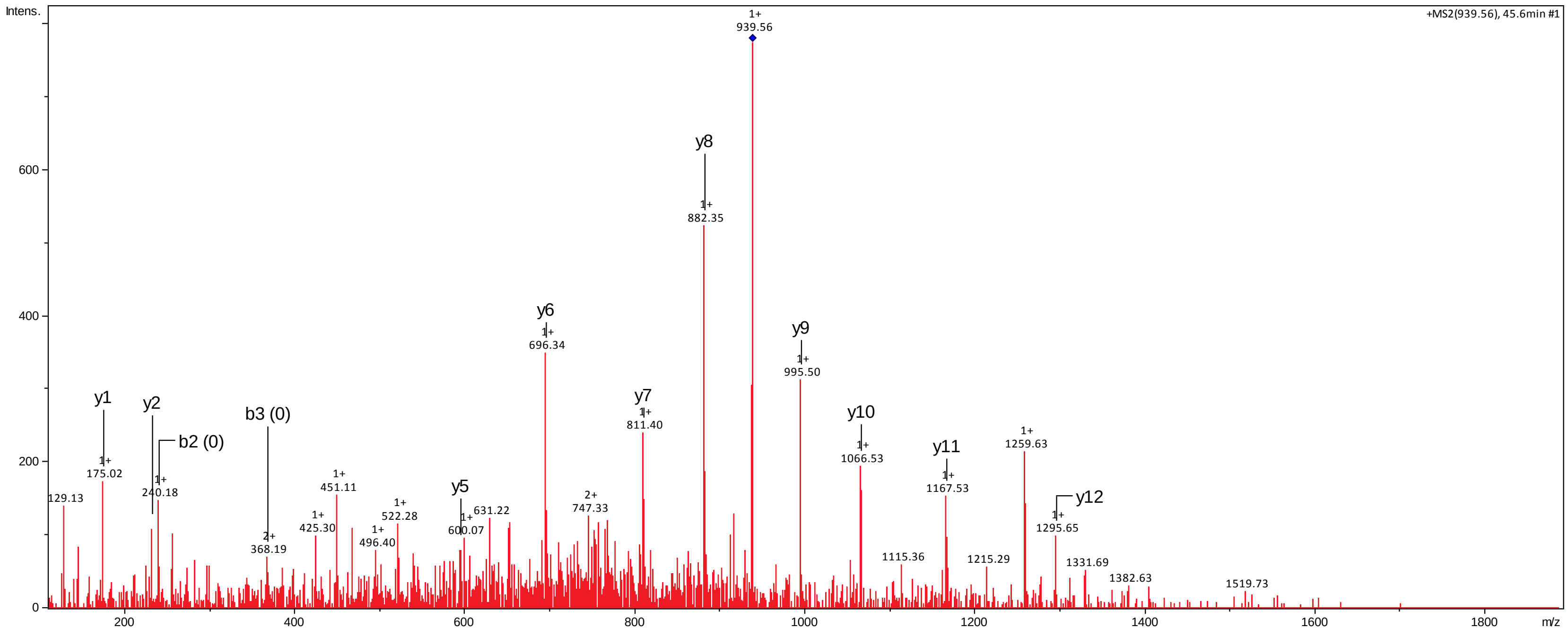

SCO Number	SCO4905
Precursor m/z	865.779
Charge	3
Retention time	133.2
Scan number	4335
Hex on peptide	2
e-value	0.000072
Site allocated?	N
Method	HCD_IT

A T P G L P A Q V F L L C G S S L V A V D R

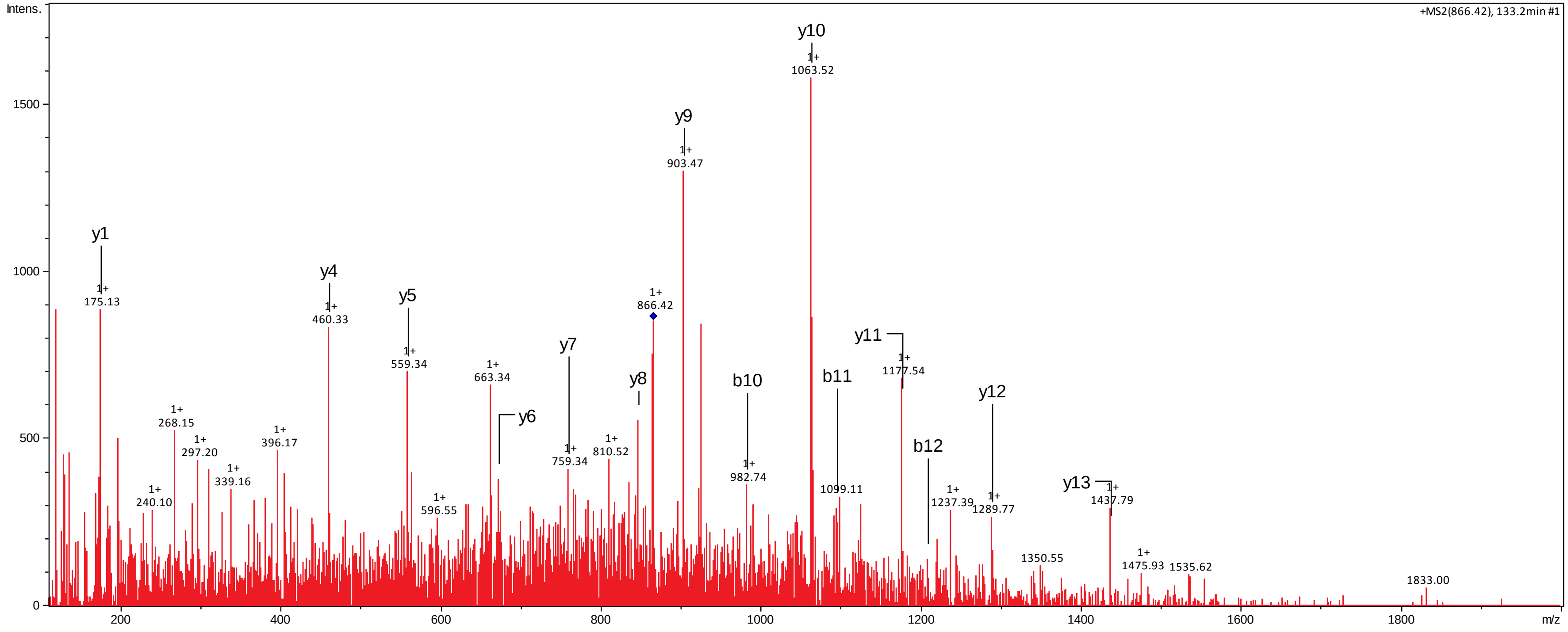

Supplement: DATA SET S2 [file mBio.01092-19-sd002.pdf]
