# Supplementary material for: Characterization of the Streptomyces coelicolor Glycoproteome Reveals Glycoproteins Important for Cell Wall Biogenesis
Source: mBio. 2019 Jun 25;10(3):e01092-19. doi: 10.1128/mBio.01092-19 (PMC6593405; doi:10.1128/mBio.01092-19)
Supplement: FIG S3 [file mBio.01092-19-sf003.docx]

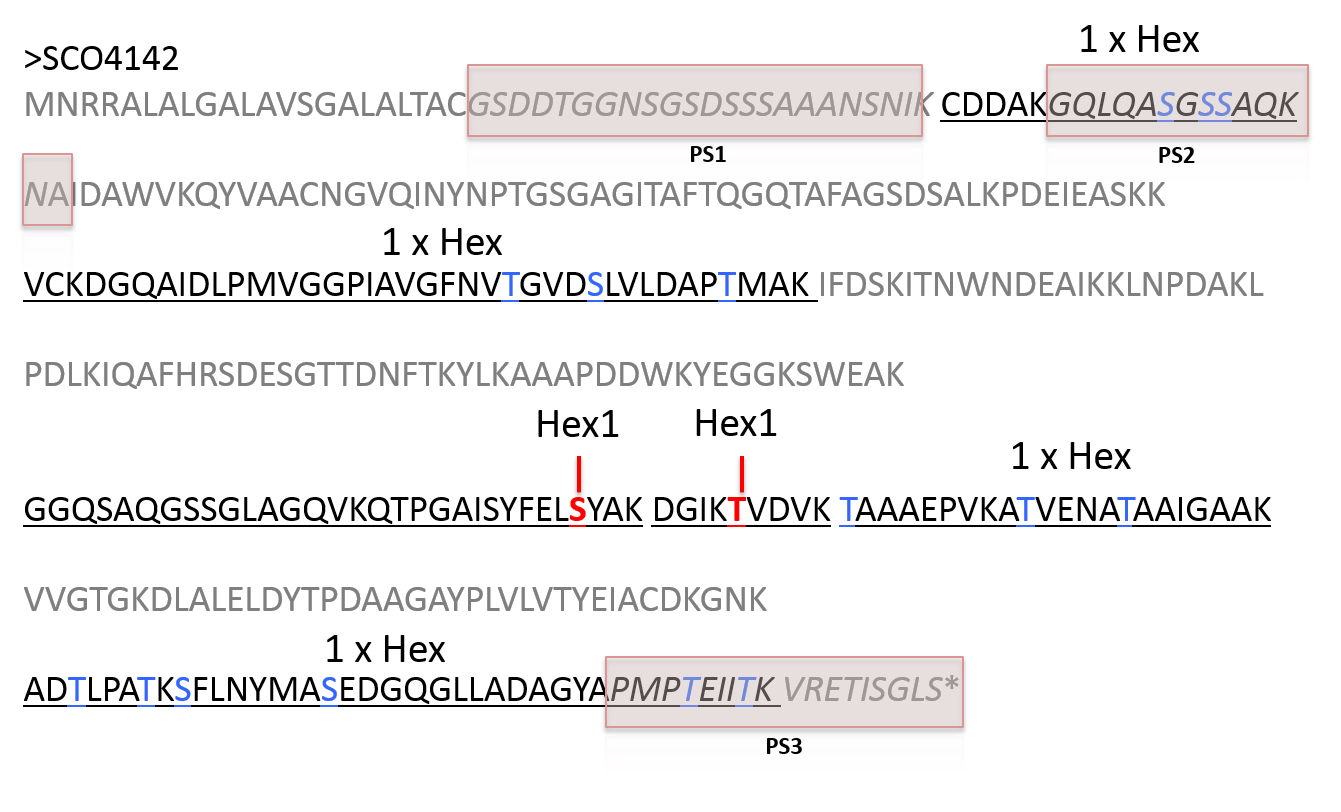


**Fig S3.** PstS glycopeptides overlap with synthetic peptides previously shown to be glycosylated in a cell free assay. Synthetic peptides PS1, PS2 and PS3 (shown in pink box) were previously tested in a cell free assay glycosylation assay. PS2 and PS3 were shown to be glycosylated. Glycopeptides identified by mass spectrometry are underlined, with validated glycosylation sites in shown in red. In glycopeptides where the glycosylation site was not validated, potential glycosylation sites are shown in blue.
