# Supplementary material for: Characterization of the Streptomyces coelicolor Glycoproteome Reveals Glycoproteins Important for Cell Wall Biogenesis
Source: mBio. 2019 Jun 25;10(3):e01092-19. doi: 10.1128/mBio.01092-19 (PMC6593405; doi:10.1128/mBio.01092-19)
Supplement: FIG S4 [file mBio.01092-19-sf004.docx]

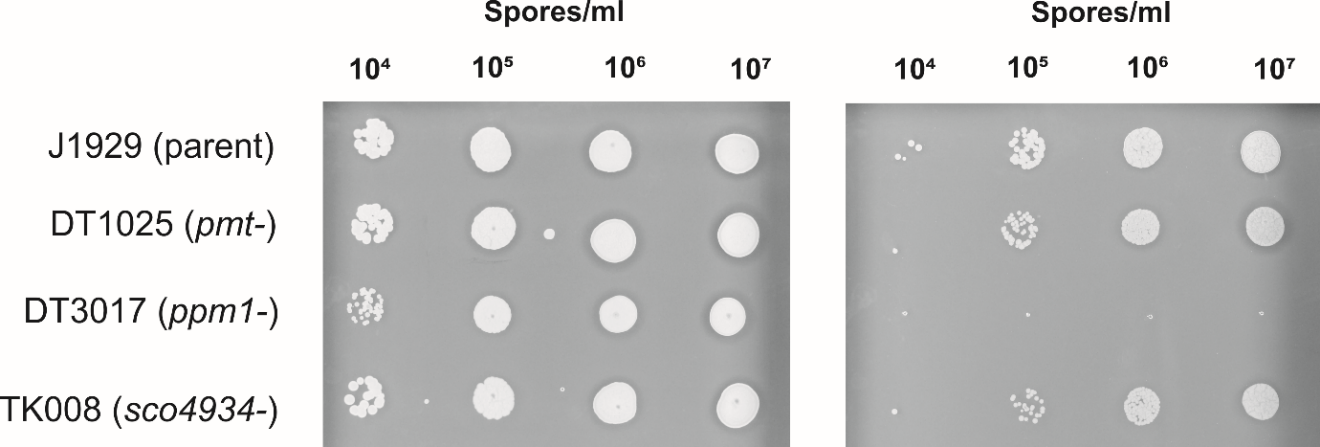


Fig S4. **Lysozyme sensitivity of TK008 (sco4934¯) compared to the parent strain J1929, DT1025 (pmt¯) and DT3017 (ppm1¯).** Spores were adjusted to 10⁸ spores/mL and a ten-fold serial dilution was carried out to get 10⁴ spores/mL. 5 µL of each spore stock was plated onto DNA without lysozyme (left-hand panel) and with 0.25 mg/mL of lysozyme (right-hand panel). Images are representative of three biological replicates.
