# Supplementary material for: Characterization of the Streptomyces coelicolor Glycoproteome Reveals Glycoproteins Important for Cell Wall Biogenesis
Source: mBio. 2019 Jun 25;10(3):e01092-19. doi: 10.1128/mBio.01092-19 (PMC6593405; doi:10.1128/mBio.01092-19)
Supplement: TABLE S1 [file mBio.01092-19-st001.docx]

**Supplemental Material Table S1. Bacterial strains, cosmids, plasmids and primers used in this work**

| ***Streptomyces* Strain** | **Genotype** | **Source** |
| --- | --- | --- |
| *S. coelicolor* J1929 | *pglY* mutant | (11) |
| *S. coelicolor* DT1025 | *pmt* mutant | (12) |
| *S. coelicolor* DT3017 | *ppm1* mutant | (13) |
| *S. coelicolor* TK006 | *sco4847* mutant | This work |
| *S. coelicolor* TK008 | *sco4934* mutant | This work |
| *S. coelicolor* TK010 | TK008: pTAK32 | This work |
| *S. coelicolor* TK013 | TK006: pTAK30 | This work |

| ***E.coli* Strain** | **Genotype** | **Source** |
| --- | --- | --- |
| DH5α | *F– Φ80lacZΔM15 (Δ(lacZYA-argF) U169 recA1 endA1 hsdR17 (rK–, mK+) phoA supE44 λ– thi-1 gyrA96 relA* | Invitrogen |
| ET12567 [pUZ8002] | *ET12567 - dam-13::Tn9, dcm-6, hsdM, hsdS; pUZ8002 – tra, neo, RP4* | (14) |

| **Cosmid** | **Description** | **Source** |
| --- | --- | --- |
| 2SCK31.2.F11 | *sco4909-sco4945*, Tn*5062* in *sco4934* at nt 5369107 | (15) |
| 5G8.1.A11 | *sco4820-sco4860*, Tn*5062* in *sco4847* at nt 5279744 | (15) |
|  |  |  |
|  |  |  |
| **Plasmid name** | **Description** | **Source** |
| pIJ10257 | attP-int-derived integration vector for the conjugal transfer of DNA from *E. coli* to *Streptomyces spp.* Contains Hygᴿ, oriT and *ermE*p* promoter. | (16) |
| pGEM7 | Cloning vector; fi oriC, SP6 and T7 RNA polymerase promoters , multiple cloning site, Ampᴿ, *lacZ* for blue/white screening | Promega |
| pTAK30 | *sco4847* in pIJ10257 | This work |
| pTAK32 | *sco4934* in pIJ10257 | This work |

| **Primer** | **Sequence** | **Description** |
| --- | --- | --- |
| TK97 | ACAGGAGGCCCCATATGGTGCCCGCTCCCAAGAAG | Forward primer - cloning *sco4847* into pIJ10257 |
| TK98 | ACTCGAGATCTCATATGGGCAGCAAGGCGCAGGAA | Reverse primer - cloning *sco4847* into pIJ10257 |
| TK101 | ACAGGAGGCCCCATATGATGACGGACGGTAAGCGG | Forward primer - cloning *sco4934* into pIJ10257 |
| TK102 | ACTCGAGATCTCATATGTCAGACCGCCGAACCCGC | Reverse primer - cloning *sco4934* into pIJ10257 |
