# Supplementary material for: Characterization of the Streptomyces coelicolor Glycoproteome Reveals Glycoproteins Important for Cell Wall Biogenesis
Source: mBio. 2019 Jun 25;10(3):e01092-19. doi: 10.1128/mBio.01092-19 (PMC6593405; doi:10.1128/mBio.01092-19)
Supplement: TEXT S1 [file mBio.01092-19-s0001.docx]

**Supplemental methods**

**Mass spectrometry analysis of glycoproteins.**

**In-gel digestion of glycoproteins.** After the separation of glycoproteins for 7 min in NuPAGE™ 10 % Bis-Tris precast gels, the gels were stained with InstantBlue Protein Stain and the protein stained regions were cut into ~1 mm pieces for processing. Gel pieces were destained by washing with 200 µL of 50 % (v/v) aqueous acetonitrile containing 25 mM (NH₄)HCO₃(2 x 20 min), then once with 200 µL of acetonitrile (5 min) and dried in a vacuum concentrator (20 min). The samples were reduced by adding 200 µL of 10 mM dithioerythritol (DTE) in 100 mM (NH₄)HCO₃ aq. and incubating 56 °C (1 h). The supernatant was discarded and the gel pieces were cooled to RT. The samples were alkylated by adding 200 µL of 50 mM iodoacetamide in 100 mM (NH₄)HCO₃ aq. and incubating in the dark (RT, 30 min). The supernatant was discarded and the gel pieces were washed in 200 µL of 100 mM (NH₄)HCO aq. (15 min). After the supernatant was discarded, the gel pieces were washed in 50 % (v/v) aqueous acetonitrile containing 25 mM (NH₄)HCO₃ (15 min). The supernatant was discarded and the gel pieces were dehydrated in 200 µL of acetonitrile (5 min). The supernatant was removed and the gel pieces were dried in a vacuum concentrator (20 min). Sequencing-grade, modified porcine trypsin (Promega) 0.2 µg in 25 mM (NH₄)HCO₃ was added to the gel pieces, and the digest was incubated at 37°C overnight. The supernatant containing digested peptides was retained. The peptides from the residual gel were extracted by adding 200 µL of 50 % (v/v) aqueous acetonitrile for 15 min. The extracts were added to the retained supernatant and the extraction was repeated twice. The combined supernatant was dried in a vacuum concentrator and the peptides were reconstituted in 20 µL of 0.1 % TFA in ddH₂O.

**LC-ESI-CID-MS/MS analysis.** Samples were loaded onto a nanoAcquity UPLC system (Waters) equipped with a nanoAcquity Symmetry C_18_, 5 µm trap (180 µm x 20 mm Waters) and a nanoAcquity HSS T3 1.8 µm C_18_ capillary column (75 μm x 250 mm, Waters). The trap wash solvent was 0.1 % (v/v) aqueous formic acid and the trapping flow rate was 10 µL/min. The trap was washed for 5 min before switching flow to the capillary column. The separation used a gradient elution of two solvents (solvent A: 0.1 % (v/v) formic acid; solvent B: acetonitrile containing 0.1% (v/v) formic acid). The flow rate for the capillary column was 300 nL/min. Column temperature was 60 °C and the gradient profile was liner 2 – 30 % B over 125 mins then linear 30-50 %B over 5 mins. All runs then proceeded to wash with 95 % solvent B for 2.5 min. The column was returned to initial conditions and re-equilibrated for 25 min before subsequent injections. The nanoLC system was interfaced with a maXis HD LC-MS/MS system (Bruker Daltonics) with a CaptiveSpray ionisation source (Bruker Daltonics). Positive ESI- MS & MS/MS spectra were acquired using AutoMSMS mode. Instrument control, data acquisition and processing were performed using Compass 1.7 software (microTOF control, Hystar and DataAnalysis, Bruker Daltonics). Instrument settings were: ion spray voltage: 1,450 V, dry gas: 3 L/min, dry gas temperature 150 °C, ion acquisition range: *m/z* 150-2,000, quadrupole low mass: 300 *m/*z, transfer time: 120 ms, collision RF: 1,400 Vpp, MS spectra rate: 5 Hz, cycle time: 3 s, and MS/MS spectra rate: 5 Hz at 2,500 cts to 20 Hz at 250,000 Hz. The collision energy and isolation width settings were automatically calculated using the AutoMSMS fragmentation table, absolute threshold 200 counts, preferred charge states: 2 – 4, singly charged ions excluded. A single MS/MS spectrum was acquired for each precursor and former target ions were excluded for 0.8 min unless the precursor intensity increased fourfold. Tandem mass spectral data were searched against a subset of the NCBInr database containing only *Streptomyces coelicolor* entries (8,578 sequences; 2,791,553 residues) using a locally-running copy of the Mascot program (Matrix Science Ltd., version 2.5), through the Bruker ProteinScape interface (version 2.1). Search criteria specified: Enzyme, trypsin; Peptide tolerance, 10 ppm; MS/MS tolerance, 0.1 Da; Instrument, ESI-QUAD-TOF; Fixed modifications, carbamidomethyl (C); Variable modifications, oxidation (M) and deamidated (NQ). Samples included the variable modifications Hex_1_ to Hex_5_ (ST). Results were filtered to accept only peptides with an expect score of 0.05 or lower.

**HCD/ETD mass spectrometry analysis.** Samples were loaded onto an UltiMate 3000 RSLCnano HPLC system (Thermo) equipped with a PepMap 100 Å C_18_, 5 µm trap column (300 µm x 5 mm Thermo) and an Acclaim PepMap RSLC, 2 µm, 100 Å, C_18_ RSLC nanocapillary column (75 μm x 150 mm, Thermo). The trap wash solvent was 0.05% (v/v) aqueous trifluoroacetic acid and the trapping flow rate was 15 µL/min. The trap was washed for 3 min before switching flow to the capillary column. The separation used gradient elution of two solvents (solvent A: aqueous 1% (v/v) formic acid; solvent B: aqueous 80% (v/v) acetonitrile containing 1% (v/v) formic acid). The flow rate for the capillary column was 300 nL/min and the column temperature was 50°C. The linear multi-step gradient profile was: 3-10% B over 8 mins, 10-35% B over 125 mins, 35-65% B over 50 mins, 65-99% B over 7 mins and then proceeded to wash with 99% solvent B for 4 min. The column was returned to initial conditions and re-equilibrated for 15 min before subsequent injections. The nanoLC system was interfaced with an Orbitrap Fusion hybrid mass spectrometer (Thermo) with a Nanospray Flex ionisation source (Thermo). Positive ESI-MS and MS^2^ spectra were acquired using Xcalibur software (version 4.0, Thermo). Instrument source settings were: ion spray voltage, 2,200 V; sweep gas, 0 Arb; ion transfer tube temperature; 275°C. MS^1^ spectra were acquired in the Orbitrap with: 120,000 resolution, scan range: *m/z* 375-1,500; AGC target, 4e^5^; max fill time, 100 ms; data type, profile. Four distinct MS^2^ strategies were employed as detailed below:

ETD_IT. MS^2^ spectra were acquired in the linear ion trap specifying: quadrupole isolation, isolation window, *m/z* 1.6; activation type, ETD; reaction time, 50 ms; reagent target, 1e6; maximum ETD reagent inject time, 200 ms; scan range, normal; scan rate, rapid; first mass, *m/z* 110; AGC target, 5e^3^; max injection time, 100 ms; data type, centroid. Data dependent acquisition was performed in top speed mode using a 1 s cycle, selecting the most intense precursors with charge states 3-8. Dynamic exclusion was performed for 50 s post precursor selection and a minimum threshold for fragmentation was set at 5e^4^.

EDT_OT. MS^2^ spectra were acquired in the Orbitrap specifying: quadrupole isolation, isolation window, *m/z* 1.6; activation type, ETD; reaction time, 50 ms; reagent target, 1e6; maximum ETD reagent inject time, 200 ms; scan range, normal; Orbitrap resolution, 30,000; first mass, *m/z* 110; AGC target, 5e^3^; max injection time, 100 ms; data type, centroid. Data dependent acquisition was performed in top speed mode using a 3 s cycle, selecting most the intense precursors. Dynamic exclusion was performed for 50 s post precursor selection and a minimum threshold for fragmentation was set at 5e^4^.

HCD_IT. MS^2^ spectra were acquired in the linear ion trap specifying: quadrupole isolation, isolation window, *m/z* 1.6; activation type, HCD; collision energy, 32%; scan range, normal; scan rate, rapid; first mass, *m/z* 110; AGC target, 5e^3^; max injection time, 100 ms; data type, centroid. Data dependent acquisition was performed in top speed mode using a 3 s cycle, with most intense precursors selected. Dynamic exclusion was performed for 50 s post precursor selection and a minimum threshold for fragmentation was set at 5e^3^.

HCD/ETD_IC. Precursors were sequentially selected and fragmented by both HCD and ETD. HCD spectra were acquired in the linear ion trap specifying quadrupole isolation, isolation window, *m/z* 1.6; activation type, HCD; collision energy, 30%; scan range, normal; scan rate, rapid; first mass, *m/z* 110; AGC target, 1e^4^; max injection time, 60 ms; data type, centroid. ETD spectra were acquired in the Orbitrap specifying: quadrupole isolation, isolation window, *m/z* 1.6; activation type, ETD; EThdD SA collision energy (15%), maximum ETD reagent inject time, 120 ms; scan range, normal; Orbitrap resolution, 60,000; first mass, *m/z* 120; AGC target, 5e^4^; max injection time, 200 ms; data type, centroid. Data dependent acquisition was performed in top N mode using a 20 precursor cycle for charge states 3-8. Highest charge state then most intense were set as selection priorities. Dynamic exclusion was performed for 50 s post precursor selection and a minimum threshold for fragmentation was set at 5e^3^.

Peak lists were generated in MGF format using Mascot Distiller (version 5, Matrix Science), stipulating a minimum signal to noise ratio of 2 and correlation (Rho) of 0.6. MGF files were searched against the *Streptomyces coelicolor* subset of the NCBInr database (8,578 sequences; 2,791,553 residues) using a locally-running copy of the Mascot search program (Matrix Science Ltd., version 2.5.1). Search criteria specified: Enzyme, trypsin; Fixed modifications, carbamidomethyl (C); Variable modifications, Hex (S,T), Hex_2_ (S,T), Hex_3_(S,T) and oxidation (M); Peptide tolerance, 10 ppm. MS/MS tolerance was set to 0.5 Da for linear ion trap data and 0.05 Da for Orbitrap data. Instrument type was set at ESI-TRAP, ETD-TRAP or CID + ETD as appropriate. Results were filtered to accept only peptides with expect scores of 0.05 or lower.

**Bioinformatic tools for the prediction of subcellular localisation of glycoproteins**. Predicted transmembrane domains were identified using TMHMM server 2.0 (1) . Predicted lipoproteins were identified using the LipoP 1.0 server (2). Signal peptides were predicted using SignalP 4.1 Server and the TatP 1.0 server (3, 4). **References for supplemental methods:**

1. Krogh A, Larsson B, Von Heijne G, & Sonnhammer EL (2001) Predicting transmembrane protein topology with a hidden Markov model: application to complete genomes. *Journal of molecular biology* 305(3):567-580.

2. Juncker AS*, et al.* (2003) Prediction of lipoprotein signal peptides in Gram‐negative bacteria. *Protein Science* 12(8):1652-1662.

3. Bendtsen JD, Nielsen H, Widdick D, Palmer T, & Brunak S (2005) Prediction of twin-arginine signal peptides. *BMC bioinformatics* 6(1):167.

4. Petersen TN, Brunak S, von Heijne G, & Nielsen H (2011) SignalP 4.0: discriminating signal peptides from transmembrane regions. *Nature methods* 8(10):785.
